# Clinical Efficacy and Safety of Alirocumab After Acute Coronary Syndrome According to Achieved Level of Low-Density Lipoprotein Cholesterol: A Propensity Score–Matched Analysis of the ODYSSEY OUTCOMES Trial

**DOI:** 10.1161/CIRCULATIONAHA.120.049447

**Published:** 2021-01-13

**Authors:** Gregory G. Schwartz, Philippe Gabriel Steg, Deepak L. Bhatt, Vera A. Bittner, Rafael Diaz, Shaun G. Goodman, J. Wouter Jukema, Yong-Un Kim, Qian H. Li, Garen Manvelian, Robert Pordy, Timothée Sourdille, Harvey D. White, Michael Szarek

**Affiliations:** 1 Division of Cardiology, School of Medicine, University of Colorado, Aurora (G.G.S., M.S.).; 2 Université de Paris, Assistance Publique-Hôpitaux de Paris, Hôpital Bichat, INSERM U1148, France (P.G.S.).; 3 Imperial College, Royal Brompton Hospital, London, UK (P.G.S.).; 4 Brigham and Women’s Hospital Heart & Vascular Center and Harvard Medical School, Boston, MA (D.L.B.).; 5 Division of Cardiovascular Disease, University of Alabama at Birmingham (V.A.B.).; 6 Estudios Cardiológicos Latinoamérica, Instituto Cardiovascular de Rosario, Argentina (R.D.).; 7 Canadian VIGOUR Centre, University of Alberta, Edmonton, and St. Michael’s Hospital, University of Toronto, Ontario, Canada (S.G.G.).; 8 Department of Cardiology, Leiden University Medical Center, The Netherlands (J.W.J.).; 9 Sanofi, Paris, France (Y.-U.K.).; 10 Regeneron Pharmaceuticals Inc, Tarrytown, NY (Q.H.L., G.M., R.P.).; 11 Sanofi, Bridgewater, NJ (T.S.).; 12 Green Lane Cardiovascular Services, Auckland City Hospital, New Zealand (H.D.W.).; 13 CPC Clinical Research, University of Colorado School of Medicine, Aurora (M.S.).; 14 State University of New York, Downstate School of Public Health, Brooklyn (M.S.).

**Keywords:** acute coronary syndrome, hydroxymethylglutaryl-CoA reductase inhibitors, lipoproteins, LDL, lipoprotein(a), PCSK9 protein, human

## Abstract

Recent international guidelines have lowered recommended target levels of low-density lipoprotein cholesterol (LDL-C) for patients at very high risk for major adverse cardiovascular events (MACE). However, uncertainty persists whether additional benefit results from achieved LDL-C levels below the conventional targets. Inferences from previous analyses are limited because patients who achieve lower versus higher LDL-C on lipid-lowering therapy differ in other characteristics prognostic for MACE and because few achieved very low LDL-C levels. To overcome these limitations, we performed a propensity score–matching analysis of the ODYSSEY OUTCOMES trial (Evaluation of Cardiovascular Outcomes After an Acute Coronary Syndrome During Treatment With Alirocumab) which compared alirocumab with placebo in 18 924 patients with recent acute coronary syndrome receiving intensive or maximum-tolerated statin treatment.

**Methods:**

Patients on alirocumab were classified in prespecified strata of LDL-C achieved at 4 months of treatment: <25 (n=3357), 25 to 50 (n=3692), or >50 mg/dL (n=2197). For each stratum, MACE (coronary heart disease death, nonfatal myocardial infarction, ischemic stroke, or hospitalization for unstable angina) after month 4 was compared in patients receiving placebo with similar baseline characteristics and adherence by using 1:1 propensity score matching.

**Results:**

Across achieved LDL-C strata of the alirocumab group, patients differed by baseline LDL-C, lipoprotein(a), use of intensive statin therapy, study medication adherence, and other demographic, medical history, biometric, and laboratory criteria. After propensity score matching, characteristics were similar in corresponding patients of the alirocumab and placebo groups. Treatment hazard ratio, 95% CI, and absolute risk reduction (number per 100 patient-years) for MACE were similar in those with achieved LDL-C <25 mg/dL (hazard ratio, 0.74 [95% CI, 0.62–0.89]; absolute risk reduction, 0.92) or 25 to 50 mg/dL (hazard ratio, 0.74 [95% CI, 0.64–0.87]; absolute risk reduction, 1.05). Patients with achieved LDL-C >50 mg/dL had poorer adherence and derived less benefit (hazard ratio, 0.87 [95% CI, 0.73–1.04]; absolute risk reduction, 0.62). No safety concerns were associated with a limited period of LDL-C levels <15 mg/dL.

**Conclusions:**

After accounting for differences in baseline characteristics and adherence, patients treated with alirocumab who achieved LDL-C levels <25 mg/dL had a reduction in the risk of MACE that was similar to that of patients who achieved LDL-C levels of 25 to 50 mg/dL.

**Registration:**

URL: https://www.clinicaltrials.gov; Unique identifier: NCT01663402.

## Abstract 2

Supplemental Digital Content is available in the text.

**Clinical Perspective**

**What Is New?**

- In patients with recent acute coronary syndrome receiving optimized statin treatment, risk of major adverse cardiovascular events was evaluated in 3 strata of low-density lipoprotein cholesterol (LDL-C) achieved with the PCSK9 (proprotein convertase subtilisin/kexin type 9) inhibitor alirocumab. In each stratum, propensity score matching was used to compare the risk of major adverse cardiovascular events with that of patients on placebo with similar baseline characteristics and study medication adherence.
- Absolute and relative treatment benefit of alirocumab was similar in patients with achieved LDL-C <25 or 25 to 50 mg/dL. Patients with achieved LDL-C >50 mg/dL had poorer adherence and derived less benefit.

**What Are the Clinical Implications?**

- Recent international guidelines have lowered LDL-C goals for patients at very high risk for major adverse cardiovascular events to levels <55 mg/dL and, in some cases, <40 mg/dL. However, the benefits of achieving LDL-C levels significantly below these goals remain uncertain.
- This study indicates that patients who achieved an LDL-C level <25 mg/dL with alirocumab had a reduction in major adverse cardiovascular events similar to those who achieved levels of 25 to 50 mg/dL.

In their 1985 Nobel Prize lecture, Brown and Goldstein suggested that a physiological concentration of low-density lipoprotein cholesterol (LDL-C) for humans may be in the range of 25 to 60 mg/dL.^1^ In fact, levels in that range are present at birth or in adulthood in hunter-gatherer populations with a very low prevalence of atherosclerotic cardiovascular disease.^2–4^ In contrast, the mean LDL-C concentration among US adults who are untreated with cholesterol-lowering medication is ≈120 mg/dL,^5^ accompanied by a high population risk of major adverse cardiovascular events (MACE). Lipid lowering with statins, ezetimibe, or PCSK9 (proprotein convertase subtilisin/kexin type 9) inhibitors reduces LDL-C concentration and MACE across a broad range of populations at risk.^6–10^

An important unresolved question is what concentration of LDL-C achieved with lipid-lowering therapy is optimal to reduce MACE with acceptable safety. Analysis of statin trials suggests that lower achieved LDL-C is associated with a lower risk of MACE, down to LDL-C levels of 30 to 50 mg/dL.^11,12^ PCSK9 inhibitors often reduce LDL-C to even lower levels.^8,9^ In the FOURIER

trial (Further Cardiovascular Outcomes Research With PCSK9 Inhibition in Patients With Elevated Risk), which compared evolocumab with placebo in 27 564 statin-treated patients,^8^ progressively lower levels of LDL-C on assigned treatment were associated with lower risk of MACE, down to a category of patients with levels <20 mg/dL.^13^ In part on the basis of these analyses, the European Society of Cardiology and European Atherosclerosis Society issued guidelines^14^ recommending lipid-lowering therapy that achieves an LDL-C level <55 mg/dL in patients at very high risk for MACE and <40 mg/dL in those who have had recurrent MACE within the previous 2 years.

The interpretation of previous analyses relating achieved LDL-C to MACE is subject to limitations. First, baseline LDL-C and other characteristics prognostic for MACE may differ between patients who achieve higher versus lower levels of LDL-C on lipid-lowering therapy^13^ and could confound relationships between achieved LDL-C and MACE. Second, calculated or directly measured LDL-C includes cholesterol contained in lipoprotein(a). Consequently, higher achieved LDL-C levels may reflect higher lipoprotein(a) concentrations that are not lowered by statins or ezetimibe.^15^ Third, lower achieved LDL-C levels may reflect better adherence with lipid-lowering therapy; such patients may also be more adherent with other treatments or lifestyle modifications that reduce MACE. Last, clinical treatment decisions are based on achieving a target range of LDL-C. Regression models relating achieved LDL-C to MACE derived across a broad, continuous range of achieved LDL-C may not accurately reflect risk in a specific target range. Moreover, in regression analyses that include patients from both active treatment and placebo groups,^13^ an apparent relationship between achieved LDL-C and MACE may be a surrogate for randomized treatment effects that extend beyond LDL-C.

To overcome these limitations, we performed a prespecified analysis of the ODYSSEY OUTCOMES trial (Evaluation of Cardiovascular Outcomes After an Acute Coronary Syndrome During Treatment With Alirocumab) that compared the PCSK9 inhibitor alirocumab with placebo in patients with recent acute coronary syndrome (ACS).^9^ We used propensity score matching to compare patients in 3 strata of achieved LDL-C with alirocumab to patients in the placebo group with similar baseline characteristics and study medication adherence. With this approach, we assessed MACE and the safety of alirocumab according to strata of achieved LDL-C.

## Methods

Data that support the findings of this study are available from the corresponding author on reasonable request.

### Study Design

The ODYSSEY OUTCOMES trial was a multinational, double-blind, placebo-controlled comparison of alirocumab with placebo in 18 924 patients hospitalized 1 to 12 months before randomization with an ACS.^9,16^ At each participating site, the study was approved by the responsible institutional review committee and subjects gave informed consent. On treatment with atorvastatin 40 to 80 mg daily, rosuvastatin 20 to 40 mg daily, or the maximum-tolerated dose of 1 of these statins, qualifying patients fulfilled at least 1 of the following criteria: LDL-C ≥70 mg/dL, non−high-density lipoprotein cholesterol ≥100 mg/dL, or apolipoprotein B ≥80 mg/dL. Key exclusion criteria included advanced heart failure, previous hemorrhagic stroke, and estimated glomerular filtration rate <30 mL·min^–1^·1.73 m^–2^. Qualifying patients were randomly assigned to receive alirocumab 75 mg or matching placebo given by subcutaneous injection every 2 weeks. To assess adherence, patients provided a diary with the dates of injection of study medication. Plasma lipids were measured at randomization and specified times thereafter. LDL-C was calculated with the Friedewald formula unless the calculated value was <15 mg/dL or the concurrent triglyceride level was ≥400 mg/dL. In those cases, LDL-C was measured directly by preparative ultracentrifugation and β-quantitation.

The dose of alirocumab was adjusted under blinded conditions to target an LDL-C level between 25 and 50 mg/dL. If LDL-C was >50 mg/dL at month 1 on the 75-mg dose, the dose of alirocumab was blindly increased to 150 mg at month 2. On the 150-mg dose, if 2 consecutive measurements of LDL-C were <25 mg/dL, the dose was blindly decreased back to 75 mg. On the 75-mg dose, if 2 consecutive measurements of LDL-C were <15 mg/dL, placebo was blindly substituted for alirocumab for the remainder of the study.

The primary outcome of MACE was the composite of coronary heart disease death, nonfatal myocardial infarction, hospitalization for unstable angina, or fatal or nonfatal ischemic stroke. Major coronary events (coronary heart disease death or nonfatal myocardial infarction) and all-cause death were secondary outcomes.

### MACE and Death by Strata of Achieved LDL-C on Alirocumab and in Propensity Score–Matched Patients on Placebo

Patients randomly assigned to alirocumab (n=9246) or placebo (n=9244) who did not have a primary outcome before their month 4 LDL-C measurement were eligible for inclusion in the analysis, corresponding to 97.7% of the full intention-to-treat population. Alirocumab-treated patients were classified in 3 prespecified strata of LDL-C measured at month 4 (122±28 days after randomization): >50 mg/dL (n=2197), 25 to 50 mg/dL (n=3692), or <25 mg/dL (n=3357). The month 4 time point was chosen because it was the nadir for LDL-C levels on alirocumab.

We considered the possibility that baseline characteristics or adherence with study medication, which were expected to be prognostic for MACE, differed according to stratum of achieved LDL-C in the alirocumab group. To account for potential confounding attributable to such differences, a propensity score was used to match each patient in the alirocumab group with a patient in the placebo group with similar baseline characteristics and adherence. To improve the quality of the match, the analysis used matching with replacement,^17^ so that a given placebo patient was eligible to be matched to patients treated with alirocumab in different achieved LDL-C categories, but not to multiple patients treated with alirocumab within an achieved LDL-C category. A threshold of *P*<0.1 with forward selection in a logistic regression model was used to determine which characteristics differed between patients in each month 4 LDL-C stratum of the alirocumab group and eligible patients receiving placebo. Baseline characteristics considered for matching were age, sex, geographic region; history of diabetes, current smoking, previous coronary artery bypass grafting, previous percutaneous coronary intervention, peripheral artery disease, cerebrovascular disease, heart failure, chronic obstructive pulmonary disease, and malignancy; type of index ACS (non−ST-segment–elevation myocardial infarction, ST-segment–elevation myocardial infarction, or unstable angina), revascularization for the index ACS, intensity of statin therapy at randomization (high intensity versus other); body mass index, systolic blood pressure, estimated glomerular filtration rate dichotomized at 60 mL·min^–1^·1.73 m^–^2; and baseline concentrations LDL-C and lipoprotein(a). Adherence was assessed by the number of doses of study medication injected during the 61 days preceding the month 4 LDL-C measurement, as reported in patient diaries. There was no imputation of missing adherence data. Greedy matching on propensity scores was performed with caliper of 0.25.

Baseline characteristics, incidence rates for MACE (events per 100 patient-years of observation) with 95% CI, treatment hazard ratio (HR) with 95% CI, and absolute risk reduction (ARR) after month 4 were summarized for all analysis-eligible patients for each stratum of month 4 LDL-C in the alirocumab group and for corresponding propensity score–matched patients from the placebo group. Similar procedures were used to assess major coronary events and all-cause death. Waterfall plots were created to show the percentage change in LDL-C from baseline to month 4.

### Additional Analyses

We considered the possibility that the treatment HR for MACE according to month 4 LDL-C was influenced by patients who had protocol-specified, blinded changes in alirocumab dose. To evaluate this possibility, we performed a sensitivity analysis with propensity score matching that excluded patients in the alirocumab group with any protocol-specified dose adjustment after month 4.

We also considered the possibility that the treatment HR for MACE according to achieved LDL-C was influenced by the duration of follow-up. To evaluate this, we performed a sensitivity analysis limited to patients who were randomly assigned at least 3 years before the common study end date and therefore potentially eligible for ≥3 years of follow-up.

To account for all LDL-C assessments in the study and for cumulative effects of LDL-C reduction over time, the relationship between continuous time-weighted moving average (TWMA) LDL-C and MACE was analyzed within the alirocumab group. TWMA LDL-C was calculated using baseline and postrandomization LDL-C values before a MACE event or right censoring for MACE at last follow-up and specified as a time-varying covariate in a Cox regression model. The results were used to create a spline plot with 50 mg/dL used as the reference point. This analysis consequently considers the effects of all protocol-specified blinded alirocumab dose adjustments, discontinuations of study medication, and any changes to background statin treatment.

### Safety of Alirocumab With Very Low Achieved LDL-C

We also used propensity score matching to evaluate whether achieving very low LDL-C on alirocumab, defined as 2 consecutive measurements <15 mg/dL, was associated with the risk of selected adverse events (neurocognitive events, hemorrhagic stroke, and new-onset diabetes). These patients were compared with patients from the placebo group with similar baseline characteristics, identified by 1:3 propensity score matching. Neurocognitive events were investigator reported. Hemorrhagic stroke was adjudicated by a blinded clinical events committee.^16^ New-onset diabetes was assessed among patients without diabetes at baseline. It was defined by a postrandomization diabetes-related adverse event, new use of diabetes medication, hemoglobin A_1c_ ≥6.5%, or 2 measurements of fasting plasma glucose ≥126 mg/dL (7 mmol/L). Possible cases of new-onset diabetes were reviewed and adjudicated by a panel of blinded expert physicians.^18^ Alirocumab had no overall effect on the risk of any of these adverse events compared with placebo^9^; therefore, the current analysis focused on whether risk was increased in patients with very low levels of LDL-C on alirocumab.

## Results

A total of 18 924 patients were randomly assigned at 1315 sites in 57 countries (Figure I in the Data Supplement). The risk of MACE and death was reduced with alirocumab versus placebo (MACE, HR, 0.85 [95% CI, 0.78−0.93]; *P*<0.001; death, HR, 0.85 [95% CI, 0.73−0.98]; *P*=0.03) over a median follow-up of 2.8 years.^9,10^ Follow-up after month 4 assessment was 2.4 years (Q1−Q3, 1.9−3.0).

### Patient Characteristics

Table 1 shows baseline characteristics, medication adherence, median LDL-C concentration achieved at month 4, and median follow-up after month 4 for the full trial cohort, analysis-eligible cohort, patients in each of the 3 strata of LDL-C achieved at month 4 in the alirocumab group, and corresponding patients from the placebo group identified by propensity score matching. Baseline characteristics were similar in the full trial and analysis cohorts. In the alirocumab group of the analysis cohort, there were significant differences in baseline characteristics among the 3 strata of achieved LDL-C at month 4. As expected, there was a gradient of baseline LDL-C, with median levels of 99, 88, and 79 mg/dL in the >50, 25 to 50, and <25 mg/dL strata, respectively. Baseline lipoprotein(a) was higher in patients in the >50 and 25 to 50 mg/dL achieved LDL-C strata than in the <25 mg/dL stratum. Nonlipid baseline characteristics also differed across achieved LDL-C strata. Each stratum differed in the representation of geographic regions. The >50 mg/dL stratum was characterized by younger age, female sex, and greater use of revascularization procedures for the index ACS. The 25 to 50 mg/dL stratum was characterized by less intensive statin therapy and lower prevalence of diabetes and heart failure. The <25 mg/dL stratum was characterized by more intensive statin therapy, male sex, higher systolic blood pressure, and lower prevalence of heart failure. Adherence with study medication was significantly poorer in the >50 mg/dL stratum (77.2% with ≥4 doses) than in the 25 to 50 mg/dL (95.3%) or <25 mg/dL (97.3%) strata (latter 2 strata each *P*<0.001 versus >50 mg/dL stratum).

**Table 1.**
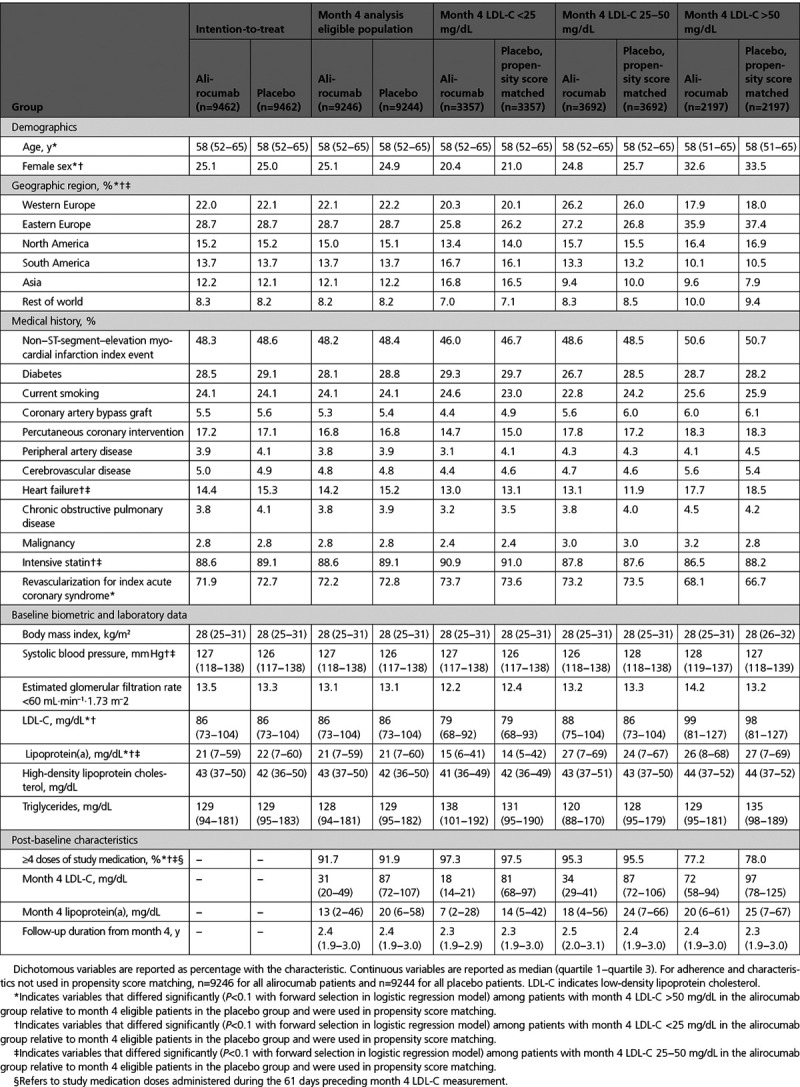
**Characteristics of Patients in the Alirocumab Group According to Levels of LDL-C Achieved at Month 4 and in Propensity Score–Matched Patients From the Placebo Group**

Of 9244 patients eligible for analysis in the placebo group, 4449 and 2292 were matched to a patient from the alirocumab group in a single achieved LDL-C category and in >1 achieved LDL-C category, respectively. After propensity score matching, each subgroup of the alirocumab group based on month 4 achieved LDL-C was well-matched to an equal number of patients from the placebo group with all standardized differences <0.06 (Table I in the Data Supplement). Median LDL-C levels at month 4 in the entire analysis-eligible placebo and alirocumab groups were 87 and 31 mg/dL, respectively. Among patients in the alirocumab group, median LDL-C levels at month 4 were 72, 34, and 18 mg/dL in the >50, 25 to 50, and <25 mg/dL strata, corresponding to median absolute decreases from baseline of 27, 54, and 61 mg/dL and median percentage decreases of 23%, 62%, and 78%, respectively. Median time-weighted LDL-C values after month 4 in the 3 strata were 71, 39, and 30 mg/dL, indicating an upward drift after month 4 in the 2 lower strata.

**Figure 1.**
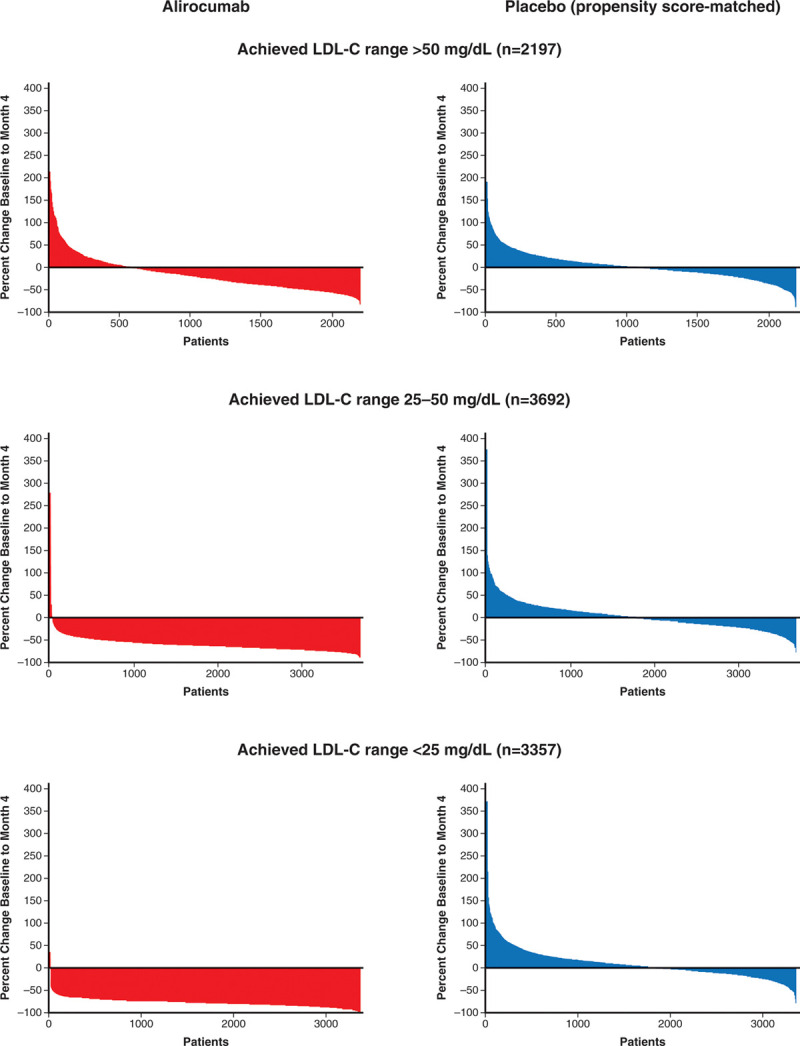
**Waterfall plots of percent change in LDL-C from baseline to month 4.** LDL-C indicates low-density lipoprotein cholesterol.

Figure 1 shows waterfall plots of the percentage change in LDL-C from baseline to month 4 according to stratum of month 4 LDL-C in the alirocumab group (Left), and in the corresponding propensity score–matched patients in the placebo group (Right). Among patients in the alirocumab group with achieved LDL-C 25 to 50 or <25 mg/dL, nearly all demonstrated a reduction in LDL-C from baseline, and 81.3% and 99.3% of those 2 subgroups demonstrated reductions of at least 50%. In contrast, among patients in the alirocumab group with achieved LDL-C >50 mg/dL, 26.4% showed an increase of LDL-C from baseline, 5.6% showed an increase from baseline of at least 50%, and only 18.2% showed a decrease from baseline of at least 50%. These differences were accompanied by differences in patient-reported adherence with study medication. At least 4 doses of study medication were expected during the 61-day period before the month 4 LDL-C measurement. In the 25 to 50 and <25 mg/dL strata, 95.3% and 97.3% of patients reported administering at least 4 doses. In contrast, in the >50 mg/dL stratum, 77.2% reported such adherence (*P*<0.001 for >50 mg/dL versus other strata; *P*<0.001 for 25−50 versus <25 mg/dL). When the >50 mg/dL achieved LDL-C stratum of the alirocumab group was dichotomized at the median percentage change from baseline, 35.5% of those with a percentage LDL-C reduction less than the median or with an increase from baseline reported self-administering fewer than 4 doses, compared with 10.2% of those with percentage LDL-C reductions larger than the median. Thus, an achieved LDL-C >50 mg/dL was associated with poorer adherence with alirocumab treatment, particularly among those with small percentage decreases or increases in LDL-C from baseline, and provides an explanation for the smaller than expected contrast in median LDL-C levels with matched patients in the placebo group (Table 1).

Waterfall plots for the placebo group showed that similar numbers of patients exhibited increases and decreases from baseline in LDL-C, with median changes from baseline of 1%, 0%, and 2% in the subgroups matched to patients in the alirocumab group with achieved LDL-C >50, 25 to 50, or <25 mg/dL, respectively. The variability of LDL-C change from baseline to month 4 in the placebo subgroups reflects intraindividual variation in LDL-C and possibly fluctuating adherence with background statin treatment.

### MACE and Death According to Achieved LDL-C

**Figure 2.**
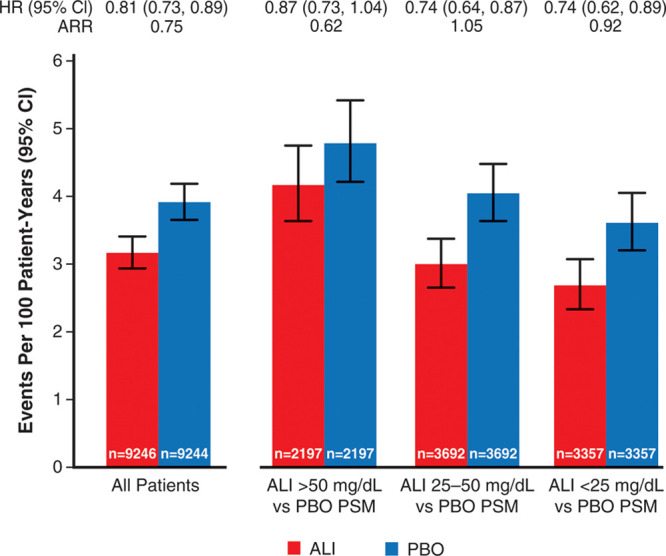
**Incidence rates for major adverse cardiovascular events after month 4 according to ranges of low-density lipoprotein cholesterol achieved at month 4 in the alirocumab group and in propensity score–matched patients from the placebo group.** ALI indicates alirocumab; ARR, absolute risk reduction; PBO, placebo; and PSM, propensity score-matched.

Table 2 shows the incidence of MACE and death after month 4 in the analysis-eligible alirocumab group according to strata of LDL-C at month 4, and in the analysis-eligible placebo group in aggregate and after propensity score matching. Figure 2 shows incidence rates for MACE after month 4 in all eligible patients, in each achieved LDL-C stratum of the alirocumab group, and in corresponding propensity score–matched subgroups of the placebo group. The 1577 events after month 4 among all eligible patients in the alirocumab and placebo groups represent 80.7% of the intention-to-treat population events from randomization. Overall, MACE (95% CI) after month 4 occurred at rates of 3.92 (3.66−4.19) and 3.16 (2.94−3.41) per 100 patient-years of observation in the placebo and alirocumab groups (HR, 0.81 [95% CI, 0.73−0.89]; ARR, 0.75 per 100 patient-years). In the achieved LDL-C strata of >50, 25 to 50, and <25 mg/dL at month 4 in the alirocumab group, the incidence rates (95% CI) for MACE after month 4 decreased progressively, from 4.17 (3.64−4.76) to 3.00 (2.65−3.37) and to 2.69 (2.34−3.07) per 100 patient-years. In the corresponding propensity score–matched patients from the placebo group, incidence rates for MACE also decreased progressively, from 4.80 (4.22−5.43) to 4.04 (3.64−4.48) and to 3.61 (3.21−4.05) per 100 patient-years. Based on these incidence rates, risk reduction with alirocumab was least in the achieved LDL-C stratum >50 mg/dL (HR, 0.87 [95% CI, 0.73−1.04]; ARR, 0.62 per 100 patient-years). However, similar relative and absolute risk reductions with alirocumab were observed in the strata with achieved LDL-C 25 to 50 mg/dL (HR, 0.74 [95% CI, 0.64−0.87]; ARR, 1.05 per 100 patient-years) or <25 mg/dL (HR, 0.74 [95% CI, 0.62−0.89]; ARR, 0.92 per 100 patient-years). A similar pattern was observed for all-cause death, with treatment HR (95% CI) of 0.98 (0.74−1.29), 0.82 (0.64−1.06), and 0.84 (0.64−1.10) in achieved LDL-C strata >50, 25 to 50, and <25 mg/dL, respectively. Figure II in the Data Supplement shows Kaplan-Meier plots of cumulative MACE in each stratum of achieved LDL-C in the alirocumab group and corresponding propensity score–matched patients from the placebo group.

**Table 2.**
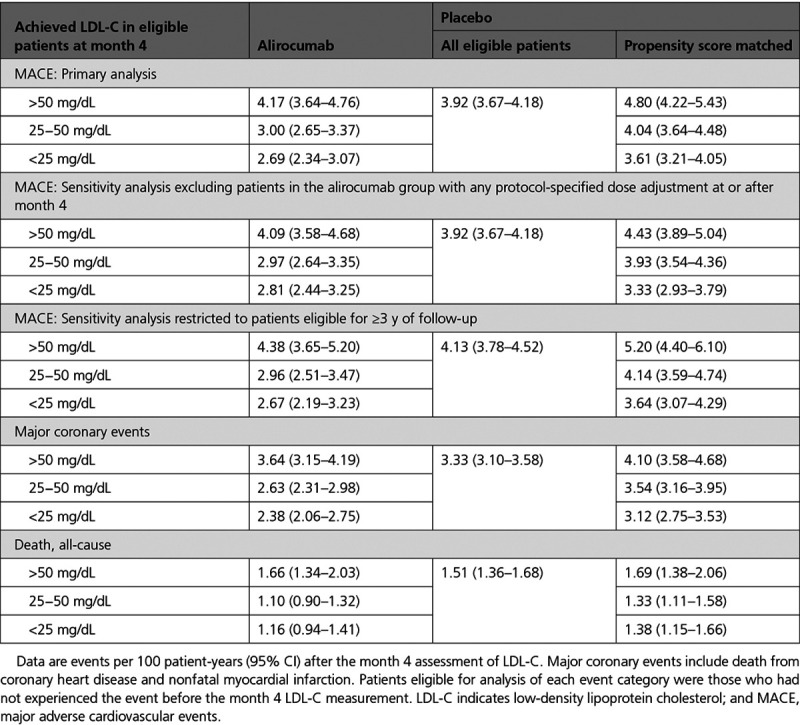
**Primary Outcome Events (MACE), Major Coronary Events, and All-Cause Death After Month 4, According to Strata of Achieved LDL-C at Month 4 in the Alirocumab Group, and in All Eligible Patients and Propensity Score–Matched Patients From the Placebo Group**

### Additional Analyses

**Figure 3.**
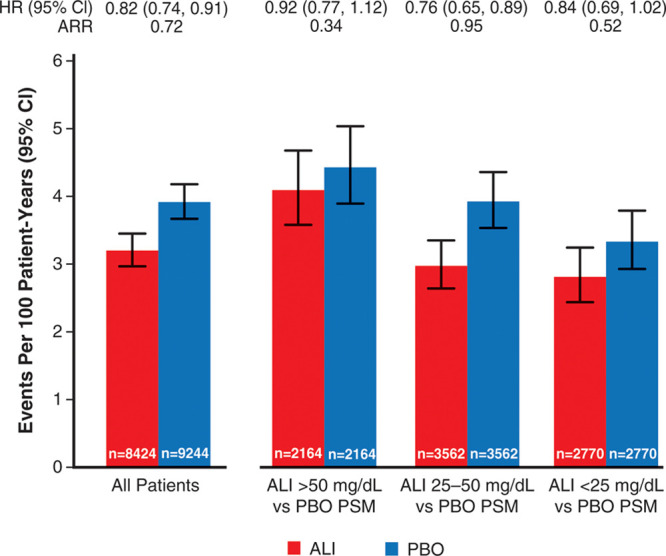
**Incidence rates for major adverse cardiovascular events after month 4 according to ranges of low-density lipoprotein cholesterol achieved at month 4 in the alirocumab group and in propensity score–matched patients from the placebo group, excluding patients in the alirocumab group with any protocol-defined dose adjustment at or after month 4.** ALI indicates alirocumab; ARR, absolute risk reduction; PBO, placebo; and PSM, propensity score-matched.

Of 9462 patients in the alirocumab group, 822 (8.7%) had protocol-specified dose adjustment after month 4, of whom 105, 130, and 587 were in month 4 LDL-C strata >50, 25 to 50, or <25 mg/dL, respectively. Table II in the Data Supplement summarizes the types of dose adjustments. In a sensitivity analysis that excluded these 822 patients, median time-weighted LDL-C after month 4 was 71, 38, and 28 mg/dL in month 4 LDL-C strata >50, 25 to 50, and <25 mg/dL of the alirocumab group. Incidence rates (95% CI) for MACE in these strata were similar to those in the base-case analysis (Table 2 and Figure 3) at 4.09 (3.58−4.68), 2.97 (2.64−3.35), and 2.81 (2.44−3.25) per 100 patient-years. Incidence rates in corresponding propensity score–matched patients from the placebo group were 4.43 (3.89−5.04), 3.93 (3.54−4.36), and 3.33 (2.93−3.79) per 100 patient-years. Resulting treatment HRs (95% CI) were 0.92 (0.77−1.12), 0.76 (0.65−0.89), and 0.84 (0.69−1.02) with ARRs 0.34, 0.95, and 0.52 per 100 patient-years in the >50, 25 to 50, and <25 mg/dL strata, respectively.

The sensitivity analysis restricted to patients eligible for at least 3 years of follow-up comprised 8011 patients. Median total follow-up was 3.4 (Q1−Q3, 3.1−3.8) years. Results were similar to the base-case analysis (Table 2). Treatment HRs (95% CI) in achieved LDL-C strata of >50, 25 to 50, and <25 mg/dL of the alirocumab group were 0.84 (0.67−1.07), 0.72 (0.58−0.88), and 0.73 (0.57−0.94), indicating nearly identical relative treatment benefit in the latter 2 strata.

**Figure 4.**
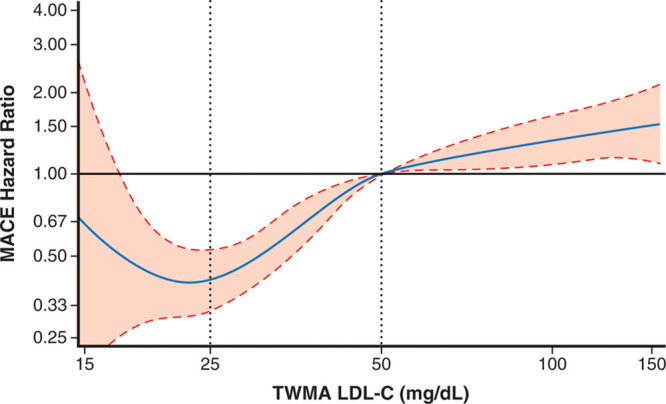
**Spline analyses of continuous TWMA LDL-C in the alirocumab group.** Hazard ratio (dashed lines represent upper and lower bounds of 95% CI) is relative to 50 mg/dL. Degree = 3, 3 knots located at LDL-C quartiles (62.5, 42.8, 31.8). *P*<0.0001 for spline effects. The risk of MACE decreased with decreasing TWMA LDL-C to a nadir at ≈23 mg/dL, without evidence of a further decrease in risk of MACE below that LDL-C level. Note that this analysis is limited to the alirocumab group and is unadjusted, and patients with lower achieved LDL-C were at lower risk for MACE for reasons in addition to their achieved LDL-C. Therefore, a nadir of MACE at ≈23 mg/dL should be interpreted cautiously and does not necessarily imply the optimal achieved LDL-C level in all patients. LDL-C indicates low-density lipoprotein cholesterol; MACE, major adverse cardiovascular events; and TWMA, time-weighted moving average.

Figure 4 shows the unadjusted relationship between continuous TWMA LDL-C and risk of MACE in the alirocumab group, with 50 mg/dL set as the reference point. The data comprise 9580, 9513, and 6473 patient-years of exposure to LDL-C levels >50, 25 to 50, and <25 mg/dL, respectively. The spline plot shows that the log HR for MACE was relatively linear at LDL-C levels >50 mg/dL and between 25 and 50 mg/dL, with lower LDL-C associated with lower risk. However, at LDL-C levels below ≈23 mg/dL, there was no evidence of lower risk of MACE, albeit with broad confidence boundaries <20 mg/dL. This finding is consistent with the propensity score–matched analyses that showed similar treatment HRs for patients achieving LDL-C levels 25 to 50 mg/dL or <25 mg/dL with alirocumab.

### Safety of Alirocumab With Very Low Achieved LDL-C

Among 730 patients with consecutive LDL-C levels <15 mg/dL on alirocumab, an average of 6.8 months were spent below this level before blinded substitution of placebo at a median 8.3 months from randomization. Among these patients, median LDL-C at month 4 (before substitution of placebo in all but 8) was 16 (Q1−Q3, 10−24) mg/dL.

Baseline characteristics of these 730 patients differed from those of the full trial cohort (Table 3 versus Table 1). As expected, the former group had lower baseline LDL-C and lipoprotein(a). In addition, they had lower body mass index, were more likely to be male, to have diabetes, to receive intensive statin treatment, and to be enrolled in Asia or South America, but less likely to be enrolled in Europe. After propensity score matching, characteristics of the 730 patients from the alirocumab group who achieved very low LDL-C were well-balanced with the 2152 patients from the placebo group. The incidence of neurocognitive events and hemorrhagic stroke among the 730 patients in the alirocumab group who achieved very low LDL-C levels did not differ from the aggregate placebo group or from 2152 propensity score–matched patients from the placebo group (Table 4).

**Table 3.**
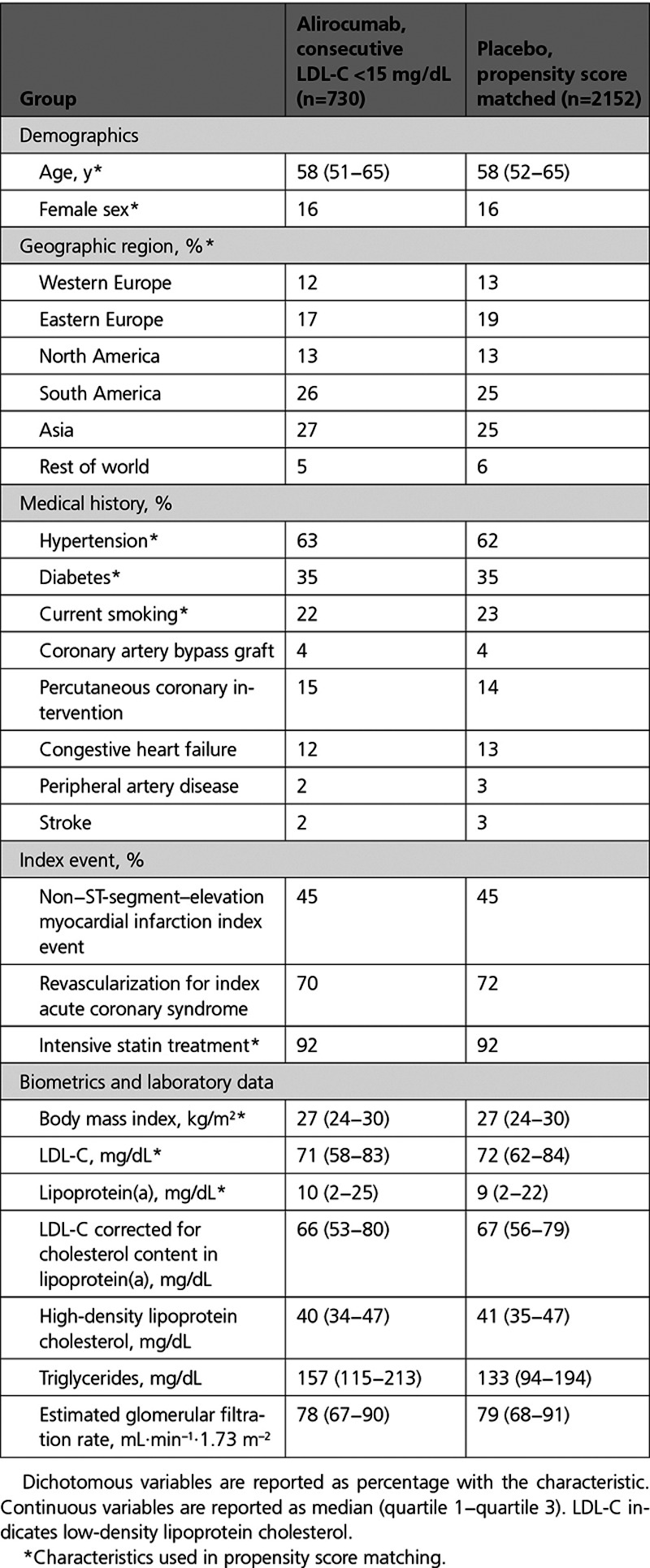
**Characteristics of Patients in the Alirocumab Group Who Achieved Consecutive LDL-C Levels <15 mg/dL and Propensity Score–Matched Patients From the Placebo Group**

**Table 4.**
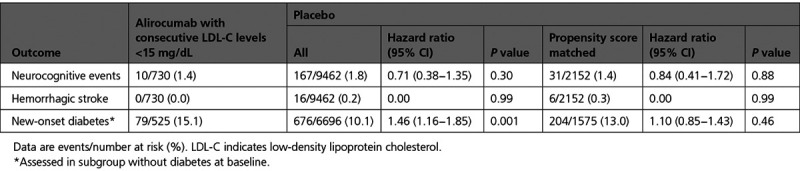
**Incidence of Selected Adverse Events in Patients in the Alirocumab Group With Consecutive Achieved LDL-C <15 mg/dL, Compared With the Aggregate Placebo Group and a Propensity Score–Matched Subgroup of the Placebo Group**

Of 6769 patients in the alirocumab group without diabetes at baseline, 525 (7.8%) achieved consecutive LDL-C levels <15 mg/dL. These patients had a greater risk of new-onset diabetes than the aggregate placebo group (15.1% versus 10.1%; HR, 1.46 [95% CI, 1.16–1.85]; *P*=0.001). However, a difference in risk of new-onset diabetes was attenuated and no longer statistically significant in comparison with 1675 propensity score–matched patients from the placebo group without diabetes at baseline (15.1% versus 13.0%; HR, 1.10 [95% CI, 0.85–1.43]; *P*=0.46; Table 4).

## Discussion

### Key Findings

This analysis of the ODYSSEY OUTCOMES trial in patients with recent ACS receiving optimized statin therapy examined the risk of MACE in 3 predefined strata of LDL-C achieved with the PCSK9 inhibitor alirocumab (>50, 25−50, or <25 mg/dL) and in propensity score–matched patients from the placebo group sharing similar baseline characteristics and adherence with study medication. As expected, patients who achieved lower LDL-C levels on alirocumab had lower baseline LDL-C. In addition, other characteristics differed among the 3 achieved LDL-C strata, including baseline levels of lipoprotein(a) and self-reported adherence with study medication. After propensity score matching, these differences were mitigated.

The propensity score–matched analysis yielded the following key observations. The incidence of MACE declined monotonically from highest to lowest achieved LDL-C stratum of the alirocumab group and in corresponding propensity score–matched patients from the placebo group. Patients in the alirocumab group with achieved LDL-C <25 mg/dL had relative and absolute reductions in the risk of MACE similar to patients with achieved LDL-C 25 to 50 mg/dL. Findings were consistent in sensitivity analyses that excluded patients with protocol-specified changes in alirocumab dose or were limited to patients eligible for at least 3 years of follow-up. Similar patterns were observed for major coronary events and all-cause death according to achieved LDL-C stratum.

A spline analysis in the alirocumab group based on TWMA LDL-C suggested a nadir in the risk of MACE at ≈23 mg/dL, without evidence of lower risk below that LDL-C level. A qualification of this analysis is that it is limited to the alirocumab group and does not compare risk with similar patients from the placebo group.

Patients with achieved LDL-C >50 mg/dL with alirocumab had poorer adherence with assigned treatment and a smaller reduction in LDL-C than expected from previous studies where adherence was confirmed by plasma drug concentrations.^19^ Consequently, there was less reduction in risk of MACE with alirocumab than in the 2 lower strata of achieved LDL-C. Failure to achieve expected LDL-C reduction with a PCSK9 inhibitor or other lipid-lowering therapy should alert treating physicians to the possibility of poor adherence.

There was no apparent excess risk of hemorrhagic stroke, neurocognitive disorders, or new-onset diabetes in patients who achieved LDL-C levels <15 mg/dL (albeit for a limited time; median 6.8 months) before being switched blindly to placebo. In fact, there were no cases of hemorrhagic stroke in that group.

### Findings in the Context of Other Evidence

Epidemiological and genetic data have provided conclusive evidence that lower levels of LDL-C within a normal physiological range are associated with lower risk of incident coronary heart disease.^20^ Analyses of pooled data from statin trials provide strong evidence that lowering LDL-C from elevated baseline levels reduces MACE in patients with or without established cardiovascular disease.^7^ However, defining an optimal range of achieved LDL-C on lipid-lowering therapy to reduce cardiovascular risk has been an elusive goal. Before the advent of PCSK9 inhibitors, few patients treated with lipid-lowering drugs achieved levels <25 mg/dL. Although some have offered the opinion that lower is better without limit on achieved LDL-C,^21^ most biological relationships have a physiological operating range beyond which beneficial effects may not be expected. Also, the association of lower achieved LDL-C with lower cardiovascular risk may be confounded by other characteristics that are associated with both variables, including lower baseline and lifetime levels of LDL-C and lipoprotein(a) that might result in a lower burden of atherosclerosis. A lower achieved LDL-C level on lipid-lowering therapies may identify patients who are more adherent with those therapies, and, by extension, possibly more adherent with other treatments and lifestyle modifications that reduce cardiovascular risk.

One potential way to overcome such confounding is to use linear regression models with adjustment for baseline characteristics. That approach was used in an analysis of the FOURIER trial that compared the PCSK9 inhibitor evolocumab with placebo.^13^ It showed a monotonic relationship of achieved LDL-C to risk of MACE that extended below an LDL-C level of 0.5 mmol/L (≈20 mg/dL). However, the analysis of FOURIER included patients from both treatment groups, was adjusted only for variables that were associated with an achieved LDL-C <0.5 mmol/L, and did not consider lipoprotein(a) or adherence measures. In addition, regression coefficients derived over a broad range of LDL-C may not portray actual risk in a specific range of achieved LDL-C. The approach used in the present analysis differs in several respects. The propensity score–matching procedure considered multiple variables associated with each of 3 strata of LDL-C achieved with alirocumab and predictive of MACE, including lipoprotein(a) and adherence, and compared the risk of MACE with that of similar patients from the placebo group. In that regard, propensity score matching provides a graphical comparison of outcomes in matched patients from both treatment groups.

The Cholesterol Treatment Trialists analyzed data from 170 000 participants in randomized, controlled trials of statins and found that the risk of MACE declined with an HR of 0.78 for each 1 mmol/L reduction in LDL-C,^7^ suggesting a log-linear relationship between reductions in LDL-C and MACE. Based on the observed changes in median LDL-C from baseline to the time-weighted level after month 4, the Cholesterol Treatment Trialists relationship predicts treatment HRs for alirocumab of 0.84, 0.73, and 0.73 in achieved LDL-C strata of >50, 25 to 50, and <25 mg/dL, respectively. These correspond very closely to the observed treatment HRs of 0.87, 0.74, and 0.74 in propensity score–matched comparisons. The findings are qualitatively consistent with a log-linear relationship between MACE and LDL-C reduction that may flatten at very low achieved LDL-C levels attributable to smaller absolute LDL-C reductions. Some investigators have proposed that the relationship between MACE and achieved LDL-C in statin trials is better fit with a sigmoidal than with a log-linear model, the former predicting a diminished slope at LDL-C levels <50 mg/dL.^22^

Although the strata of achieved LDL-C prespecified in the design of the ODYSSEY OUTCOMES trial do not correspond precisely with those in subsequently published guidelines of the European Society of Cardiology and European Atherosclerosis Society,^14^ the current findings are consistent, in general, with guidance to achieve an LDL-C level <55 mg/dL in very-high-risk patients or a level <40 mg/dL in those with recurrent cardiovascular events. In showing no clear advantage to an achieved LDL-C <25 mg/dL versus a level 25 to 50 mg/dL, the current findings are also consistent with guidance of the American Heart Association and American College of Cardiology to take a cautious approach when achieved levels of LDL-C are <25 mg/dL.^23^

### Limitations

Despite the use of propensity score matching, analyses based on postrandomization data such as the achieved level of LDL-C on assigned treatment may be subject to residual confounding. Our data indicate that patients who reached the lowest of 3 strata of LDL-C with alirocumab derived no greater benefit than those who reached the middle stratum. However, it is uncertain whether further lowering of LDL-C among patients in the middle stratum would have resulted in further reduction in risk of MACE. In the alirocumab group the relationship between TWMA LDL-C and MACE had a broad confidence interval at TWMA LDL-C values <20 mg/dL; accordingly, there is greater uncertainty regarding the risk of MACE in this range. Achieved LDL-C in the ODYSSEY OUTCOMES trial was influenced by protocol-specified blinded adjustment of the alirocumab dose or substitution of placebo in some patients. However, findings were not substantially affected by excluding such patients from the analysis. Findings over a median observation of 2.4 years (3.4 years in sensitivity analysis) may not necessarily presage efficacy over a longer period. Likewise, the safety of relatively brief exposure to very low LDL-C levels may not inform the long-term safety of such levels. Notwithstanding these limitations, the current findings may provide a practical framework to use PCSK9 inhibitors to the best advantage.

### Conclusions

Among patients with recent ACS and LDL-C ≥70 mg/dL on optimized statin therapy, a significant number achieved LDL-C levels <25 mg/dL after additional treatment with the PCSK9 inhibitor alirocumab. Patients who achieved LDL-C <25 mg/dL on alirocumab had absolute and relative reductions in the risk of MACE (compared with matched patients treated with statin and placebo) similar to patients who achieved LDL-C 25 to 50 mg/dL, whereas patients who achieved LDL-C >50 mg/dL on alirocumab had smaller reduction in MACE. A limited duration of exposure to LDL-C <15 mg/dL was not associated with identifiable safety concerns. These findings support recent American and European guidelines for cholesterol lowering in high-risk patients^14,23^ that recommend treatment additional to statins when LDL-C remains >70 mg/dL and LDL-C goals <55 mg/d, possibly <40 mg/dL, but not necessarily <25 mg/dL.

## Acknowledgments

We thank the patients, study coordinators, and investigators who participated in this trial. Sophie Rushton-Smith, PhD (MedLink Healthcare Communications, London) provided editorial assistance in the preparation of the manuscript (limited to editing for style, referencing, and figure and table editing) and was funded by Sanofi. Author Contributions: Concept and design: G.G. Schwartz, P.G. Steg, V.A. Bittner, and M. Szarek. Acquisition of the data: Drs Schwartz, Steg, Bhatt, Bittner, Diaz, Goodman, Jukema, White, and Szarek. Analysis or interpretation of the data: Drs Schwartz, Steg, and Szarek. Drafting of the manuscript: Dr Schwartz. Critical revision of the manuscript: all. Statistical analysis: Drs Szarek and Li. Obtained funding: Drs Steg and Schwartz. Administrative, technical or material support: Drs Kim, Li, Manvelian, Pordy, and Sourdille. Supervision: Drs Steg and Schwartz. Drs Schwartz and Szarek had full access to the data in the study, take responsibility for the integrity of the data and the accuracy of the data analysis, and had final responsibility for the decision to submit for publication.

## Sources of Funding

The trial was funded by Sanofi and Regeneron Pharmaceuticals, Inc. Role of the Funder/Sponsor: The protocol and statistical analysis plan were conceived by Drs Schwartz, Steg, and Szarek, developed in conjunction with the other members of the Executive Steering Committee and sponsors, and approved by responsible regulatory authorities and ethics committees. The sponsors participated in study site selection, monitoring, and supervision of data collection. Duke Clinical Research Institute led blinded end point adjudication. An independent data-monitoring committee monitored safety and efficacy data. Analyses were performed by the independent academic statistician (Dr Szarek). The manuscript was drafted by the first author with input from all authors. The Executive Steering Committee decided to publish the paper and takes responsibility for the completeness and accuracy of the data and the fidelity of the trial to the protocol.

## Disclosures

Dr Schwartz reports research support to the University of Colorado from AstraZeneca, Resverlogix, Roche, Sanofi, and The Medicines Company; he is coinventor of pending US patent 62/806,313 (“Methods for Reducing Cardiovascular Risk”) assigned in full to the University of Colorado. Dr Steg reports grants and nonfinancial support (cochair of the ODYSSEY OUTCOMES trial (Evaluation of Cardiovascular Outcomes After an Acute Coronary Syndrome During Treatment With Alirocumab); as such, he received no personal fees, but his institution has received funding for the time he has devoted to trial coordination, and he has received support for travel related to trial meetings) from Sanofi; research grants and personal fees from Bayer (Steering Committee MARINER [A Study of Rivaroxaban {JNJ-39039039} on the Venous Thromboembolic Risk of Post-Hospital Discharge Patients], grant for epidemiological study), Merck (speaker fees, grant for epidemiological studies), Sanofi (cochair of the ODYSSEY OUTCOMES trial; cochair of the SCORED trial (Effect of Sotagliflozin on Cardiovascular and Renal Events in Patients With Type 2 Diabetes and Moderate Renal Impairment Who Are at Cardiovascular Risk); consulting, speaking), Servier (Chair of the CLARIFY registry [Prospective Observational Longitudinal Registry of Patients with Stable Coronary Artery Disease]; grant for epidemiological research), and Amarin (executive steering committee for the REDUCE-IT trial [Disease Reduction of Cardiovascular Events With Icosapent Ethyl–Intervention Trial]; consulting); and personal fees from Amgen, Bristol-Myers Squibb, Boehringer Ingelheim, Pfizer, Novartis, Regeneron Pharmaceuticals, Lilly, and AstraZeneca. Dr Steg also has a European application number/patent number, issued on October 26, 2016 (No. 15712241.7), for a method for reducing cardiovascular risk. Dr Bhatt discloses the following relationships: Advisory Board: Cardax, CellProthera, Cereno Scientific, Elsevier Practice Update Cardiology, Level Ex, Medscape Cardiology, MyoKardia, PhaseBio, PLx Pharma, Regado Biosciences; Board of Directors: Boston VA Research Institute, Society of Cardiovascular Patient Care, TobeSoft; Chair: American Heart Association Quality Oversight Committee; Data Monitoring Committees: Baim Institute for Clinical Research (formerly Harvard Clinical Research Institute, for the PORTICO trial [Portico Re-sheathable Transcatheter Aortic Valve System US IDE Trial], funded by St. Jude Medical, now Abbott), Cleveland Clinic (including for the ExCEED trial [Efficacy of Secukinumab Compared to Adalimumab in Patients With Psoriatic Arthritis], funded by Edwards), Contego Medical (Chair, PERFORMANCE 2 trial [Protection Against Emboli During Carotid Artery Stenting Using the Neuroguard IEP System]), Duke Clinical Research Institute, Mayo Clinic, Mount Sinai School of Medicine (for the ENVISAGE trial [Edoxaban Compared to Standard Care After Heart Valve Replacement Using a Catheter in Patients With Atrial Fibrillation], funded by Daiichi Sankyo), Population Health Research Institute; Honoraria: American College of Cardiology (Senior Associate Editor, *Clinical Trials and News*, ACC.org; Vice-Chair, ACC Accreditation Committee), Baim Institute for Clinical Research (formerly Harvard Clinical Research Institute; RE-DUAL PCI clinical trial [Evaluation of Dual Therapy With Dabigatran vs. Triple Therapy With Warfarin in Patients With AF That Undergo a PCI With Stenting] steering committee funded by Boehringer Ingelheim; AEGIS-II [Study to Investigate CSL112 in Subjects With Acute Coronary Syndrome] executive committee funded by CSL Behring), Belvoir Publications (Editor in Chief, *Harvard Heart Letter*), Canadian Medical and Surgical Knowledge Translation Research Group (clinical trial steering committees), Duke Clinical Research Institute (clinical trial steering committees, including for the PRONOUNCE trial [A Trial Comparing Cardiovascular Safety of Degarelix Versu Leuprolide in Patients With Advanced Prostate Cancer and Cardiovascular Disease], funded by Ferring Pharmaceuticals), HMP Global (Editor in Chief, *Journal of Invasive Cardiology*), *Journal of the American College of Cardiology* (Guest Editor; Associate Editor), K2P (Co-Chair, interdisciplinary curriculum), Level Ex, Medtelligence/ReachMD (CME steering committees), MJH Life Sciences, Population Health Research Institute (for the COMPASS [Rivaroxaban for the Prevention of Major Cardiovascular Events in Coronary or Peripheral Artery Disease] operations committee, publications committee, steering committee, and USA national coleader, funded by Bayer), Slack Publications (Chief Medical Editor, *Cardiology Today’s Intervention*), Society of Cardiovascular Patient Care (Secretary/Treasurer), WebMD (CME steering committees); Other: *Clinical Cardiology* (Deputy Editor), NCDR-ACTION Registry Steering Committee (Chair), VA CART Research and Publications Committee (Chair); Research Funding: Abbott, Afimmune, Amarin, Amgen, AstraZeneca, Bayer, Boehringer Ingelheim, Bristol-Myers Squibb, Cardax, Chiesi, CSL Behring, Eisai, Ethicon, Ferring Pharmaceuticals, Forest Laboratories, Fractyl, Idorsia, Ironwood, Ischemix, Lexicon, Lilly, Medtronic, MyoKardia, Pfizer, PhaseBio, PLx Pharma, Regeneron, Roche, Sanofi, Synaptic, The Medicines Company; Royalties: Elsevier (Editor, *Cardiovascular Intervention: A Companion to Braunwald’s Heart Disease*); Site Co-Investigator: Biotronik, Boston Scientific, CSI, St. Jude Medical (now Abbott), Svelte; Trustee: American College of Cardiology; Unfunded Research: FlowCo, Merck, Novo Nordisk, Takeda. Dr Bittner reports grant support from Sanofi, Astra Zeneca, DalCor, Esperion, Bayer, The Medicines Company, and Amgen, all paid direct to her institution; and personal fees from Sanofi. Dr Diaz reports research grants from Sanofi, DalCor Pharmaceuticals, Population Health Research Institute, Duke Clinical Research Institute, the TIMI group, Amgen, Cirius, Montreal Health Innovations Coordinating Center, and Lepetit; and personal fees, as a member of the Executive Steering Committee, from Amgen and Cirius. Dr Goodman reports research grants from Daiichi-Sankyo, Luitpold Pharmaceuticals, Merck, Novartis, Servier, Regeneron Pharmaceuticals, Inc, Sanofi, Amgen, AstraZeneca, Bayer, Boehringer Ingelheim, Bristol-Myers Squibb, CSL Behring, Eli Lilly, Pfizer, and Tenax Therapeutics; honoraria from Bristol-Myers Squibb, Eli Lilly, Esperion, Fenix Group International, Ferring Pharmaceuticals, Merck, Novartis, Pfizer, Servier, Regeneron Pharmaceuticals, Inc, Sanofi, Amgen, AstraZeneca, Bayer, and Boehringer Ingelheim; and serving as a consultant or on advisory boards (or both) for AstraZeneca, Boehringer Ingelheim, Bristol-Myers Squibb, Eli Lilly, HLS Therapeutics, Pfizer, Servier, Tenax Therapeutics, Sanofi, Amgen, and Bayer. Dr Jukema reports research grants from the Netherlands Heart Foundation, the Interuniversity Cardiology Institute of the Netherlands, and the European Commission Seventh Framework Program; and research support from Amgen, Astellas, AstraZeneca, Daiichi-Sankyo, Lilly, Merck-Schering-Plow, Pfizer, Roche, and Sanofi. Drs Kim and Sourdille are employees of and hold shares in Sanofi. Drs Li, Manvelian, and Pordy are employees of Regeneron Pharmaceuticals, Inc. Dr White reports receiving grant support paid to the institution and fees for serving on a steering committee for the ODYSSEY OUTCOMES trial from Sanofi-Aventis and Regeneron Pharmaceuticals, for the ACCELERATE study (A Study of Evacetrapib in High-Risk Vascular Disease) from Eli Lilly, for the STRENGTH trial (Outcomes Study to Assess Statin Residual Risk Reduction With EpaNova in High CV Risk Patients With Hypertriglyceridemia) from Omthera Pharmaceuticals, for the SPIRE trial (The Evaluation of Bococizumab [PF-04950615; RN 316] in Reducing the Occurrence of Major Cardiovascular Events in High Risk Subjects) from Pfizer USA, for the HEART-FID study (Randomized Placebo-Controlled Trial of FCM as Treatment for Heart Failure With Iron Deficiency) from American Regent; for the CAMELLIA-TIMI study (A Study to Evaluate the Effect of Long-term Treatment With BELVIQ [Lorcaserin HC] on the Incidence of Major Adverse Cardiovascular Events and Conversion to Type 2 Diabetes Mellitus in Obese and Overweight Subjects With Cardiovascular Disease or Multiple Cardiovascular Risk Factors) from Eisai Inc, for the dal-GenE study (Effect of Dalcetrapib versus Placebo on CV Risk in a Genetically Defined Population With a Recent ACS) from DalCor Pharma UK Inc, for the AEGIS-II study from CSL Behring, for the SCORED trial, and the SOLOIST-WHF trial (Effect of Sotagliflozin on Cardiovascular Events in Patients With Type2 Diabetes Post Worsening Heart Failure) from Sanofi-Aventis Australia Pty Ltd, and for the CLEAR Outcomes Study (Evaluation of Major Cardiovascular Events in Patients With, or at High Risk for, Cardiovascular Disease Who Are Statin Intolerant Treated With Bempedoic Acid [ETC-1002] or Placebo) from Esperion Therapeutics Inc. Dr White was on the Advisory Boards for Acetelion, Sirtex, and Genentech, Inc (an affiliate of F. Hoffmann-La Roche Ltd, “Roche”; Lytics Post-PCI Advisory Board at European Society of Cardiology), and received lecture fees from AstraZeneca. Dr Szarek reports serving as a consultant or on advisory boards (or both) for CiVi, Resverlogix, Baxter, Esperion, and Regeneron Pharmaceuticals, Inc.

## Supplemental Materials

ODYSSEY OUTCOMES committees and investigators

Data Supplement Tables I and II

Data Supplement Figures I and II

## Supplementary Material



## References

[1] Brown MS Goldstein JL. A receptor-mediated pathway for cholesterol homeostasis. Science. 1986;232:34–47. doi: 10.1126/science.3513311 3513311 10.1126/science.3513311

[2] O’Keefe JH Jr Cordain L Harris WH Moe RM Vogel R. Optimal low-density lipoprotein is 50 to 70 mg/dl: lower is better and physiologically normal. J Am Coll Cardiol. 2004;43:2142–2146. doi: 10.1016/j.jacc.2004.03.046 15172426 10.1016/j.jacc.2004.03.046

[3] Parker CR Jr Carr BR Simpson ER MacDonald PC. Decline in the concentration of low-density lipoprotein-cholesterol in human fetal plasma near term. Metabolism. 1983;32:919–923. doi: 10.1016/0026-0495(83)90207-x 6888272 10.1016/0026-0495(83)90207-x

[4] Forrester JS. Redefining normal low-density lipoprotein cholesterol: a strategy to unseat coronary disease as the nation’s leading killer. J Am Coll Cardiol. 2010;56:630–636. doi: 10.1016/j.jacc.2009.11.090 20705220 10.1016/j.jacc.2009.11.090

[5] Mercado CI Gregg E Gillespie C Loustalot F. Trends in lipid profiles and descriptive characteristics of U.S. adults with and without diabetes and cholesterol-lowering medication use-National Health and Nutrition Examination Survey, 2003–2012, United States. PLoS One. 2018;13:e0193756 29509776 10.1371/journal.pone.0193756 PMC5839584

[6] Cannon CP Blazing MA Giugliano RP McCagg A White JA Theroux P Darius H Lewis BS Ophuis TO Jukema JW; IMPROVE-IT Investigators. Ezetimibe added to statin therapy after acute coronary syndromes. N Engl J Med. 2015;372:2387–2397. doi: 10.1056/NEJMoa1410489 26039521 10.1056/NEJMoa1410489

[7] Baigent C Blackwell L Emberson J Holland LE Reith C Bhala N Peto R Barnes EH Keech A Simes J; Cholesterol Treatment Trialists’ (CTT) Collaboration. Efficacy and safety of more intensive lowering of LDL cholesterol: a meta-analysis of data from 170,000 participants in 26 randomised trials. Lancet. 2010;376:1670–1681. doi: 10.1016/S0140-6736(10)61350-5 21067804 10.1016/S0140-6736(10)61350-5 PMC2988224

[8] Sabatine MS Giugliano RP Keech AC Honarpour N Wiviott SD Murphy SA Kuder JF Wang H Liu T Wasserman SM; FOURIER Steering Committee and Investigators. Evolocumab and clinical outcomes in patients with cardiovascular disease. N Engl J Med. 2017;376:1713–1722. doi: 10.1056/NEJMoa1615664 28304224 10.1056/NEJMoa1615664

[9] Schwartz GG Steg PG Szarek M Bhatt DL Bittner VA Diaz R Edelberg JM Goodman SG Hanotin C Harrington RA; ODYSSEY OUTCOMES Committees and Investigators. Alirocumab and cardiovascular outcomes after acute coronary syndrome. N Engl J Med. 2018;379:2097–2107. doi: 10.1056/NEJMoa1801174 30403574 10.1056/NEJMoa1801174

[10] Steg PG Szarek M Bhatt DL Bittner VA Brégeault MF Dalby AJ Diaz R Edelberg JM Goodman SG Hanotin C. Effect of alirocumab on mortality after acute coronary syndromes. Circulation. 2019;140:103–112. doi: 10.1161/CIRCULATIONAHA.118.038840 31117810 10.1161/CIRCULATIONAHA.118.038840 PMC6661243

[11] Boekholdt SM Hovingh GK Mora S Arsenault BJ Amarenco P Pedersen TR LaRosa JC Waters DD DeMicco DA Simes RJ Very low levels of atherogenic lipoproteins and the risk for cardiovascular events: a meta-analysis of statin trials. J Am Coll Cardiol. 2014;64:485–494. doi: 10.1016/j.jacc.2014.02.615 25082583 10.1016/j.jacc.2014.02.615 PMC4443441

[12] Hsia J MacFadyen JG Monyak J Ridker PM. Cardiovascular event reduction and adverse events among subjects attaining low-density lipoprotein cholesterol <50 mg/dl with rosuvastatin. The JUPITER trial (Justification for the Use of Statins in Prevention: an Intervention Trial Evaluating Rosuvastatin). J Am Coll Cardiol. 2011;57:1666–1675. doi: 10.1016/j.jacc.2010.09.082 21492764 10.1016/j.jacc.2010.09.082

[13] Giugliano RP Pedersen TR Park JG De Ferrari GM Gaciong ZA Ceska R Toth K Gouni-Berthold I Lopez-Miranda J Schiele F; FOURIER Investigators. Clinical efficacy and safety of achieving very low LDL-cholesterol concentrations with the PCSK9 inhibitor evolocumab: a prespecified secondary analysis of the FOURIER trial. Lancet. 2017;390:1962–1971. doi: 10.1016/S0140-6736(17)32290-0 28859947 10.1016/S0140-6736(17)32290-0

[14] Mach F Baigent C Catapano AL Koskinas KC Casula M Badimon L Chapman MJ De Backer GG Delgado V Ference BA; ESC Scientific Document Group. 2019 ESC/EAS Guidelines for the management of dyslipidaemias: lipid modification to reduce cardiovascular risk. Eur Heart J. 2020;41:111–188. doi: 10.1093/eurheartj/ehz455 31504418 10.1093/eurheartj/ehz455

[15] Kinpara K Okada H Yoneyama A Okubo M Murase T. Lipoprotein(a)-cholesterol: a significant component of serum cholesterol. Clin Chim Acta. 2011;412:1783–1787. doi: 10.1016/j.cca.2011.05.036 21672532 10.1016/j.cca.2011.05.036

[16] Schwartz GG Bessac L Berdan LG Bhatt DL Bittner V Diaz R Goodman SG Hanotin C Harrington RA Jukema JW. Effect of alirocumab, a monoclonal antibody to PCSK9, on long-term cardiovascular outcomes following acute coronary syndromes: rationale and design of the ODYSSEY outcomes trial. Am Heart J. 2014;168:682–689. doi: 10.1016/j.ahj.2014.07.028 25440796 10.1016/j.ahj.2014.07.028

[17] Dehejia RH Wahba S. Propensity score-matching methods for nonexperimental causal studies. Rev Econ Stat. 2002;84:151–161

[18] Ray KK Colhoun HM Szarek M Baccara-Dinet M Bhatt DL Bittner VA Budaj AJ Diaz R Goodman SG Hanotin C ODYSSEY OUTCOMES Committees and Investigators. Effects of alirocumab on cardiovascular and metabolic outcomes after acute coronary syndrome in patients with or without diabetes: a prespecified analysis of the ODYSSEY OUTCOMES randomised controlled trial. Lancet Diabetes Endocrinol. 2019;7:618–628. doi: 10.1016/S2213-8587(19)30158-5 31272931 10.1016/S2213-8587(19)30158-5

[19] Bays HE Rosenson RS Baccara-Dinet MT Louie MJ Thompson D Hovingh GK. Assessment of the 1% of patients with consistent < 15% reduction in low-density lipoprotein cholesterol: pooled analysis of 10 phase 3 ODYSSEY alirocumab trials. Cardiovasc Drugs Ther. 2018;32:175–180. doi: 10.1007/s10557-018-6784-z 29627892 10.1007/s10557-018-6784-z PMC5958153

[20] Ference BA Ginsberg HN Graham I Ray KK Packard CJ Bruckert E Hegele RA Krauss RM Raal FJ Schunkert H. Low-density lipoproteins cause atherosclerotic cardiovascular disease. 1. Evidence from genetic, epidemiologic, and clinical studies. A consensus statement from the European Atherosclerosis Society Consensus Panel. Eur Heart J. 2017;38:2459–2472. doi: 10.1093/eurheartj/ehx144 28444290 10.1093/eurheartj/ehx144 PMC5837225

[21] Rosenson RS Hegele RA Fazio S Cannon CP. The evolving future of PCSK9 inhibitors. J Am Coll Cardiol. 2018;72:314–329. doi: 10.1016/j.jacc.2018.04.054 30012326 10.1016/j.jacc.2018.04.054

[22] Charland SL Stanek EJ. Sigmoidal maximal effect modeling of low-density lipoprotein cholesterol concentration and annual incidence of coronary heart disease events in secondary prevention trials. Pharmacotherapy. 2014;34:452–463. doi: 10.1002/phar.1368 24877185 10.1002/phar.1368

[23] Grundy SM Stone NJ Bailey AL Beam C Birtcher KK Blumenthal RS Braun LT de Ferranti S Faiella-Tommasino J Forman DE. 2018 AHA/ACC/AACVPR/AAPA/ABC/ACPM/ADA/AGS/APhA/ASPC/NLA/PCNA guideline on the management of blood cholesterol: a report of the American College of Cardiology/American Heart Association Task Force on Clinical Practice Guidelines. Circulation. 2019;139:e1082–e1143. doi: 10.1161/CIR.0000000000000625 30586774 10.1161/CIR.0000000000000625 PMC7403606

