# Supplementary material for: Clinical Efficacy and Safety of Alirocumab After Acute Coronary Syndrome According to Achieved Level of Low-Density Lipoprotein Cholesterol: A Propensity Score–Matched Analysis of the ODYSSEY OUTCOMES Trial
Source: Circulation. 2021 Jan 13;143(11):1109–22. doi: 10.1161/CIRCULATIONAHA.120.049447 (PMC7969166; doi:10.1161/CIRCULATIONAHA.120.049447)
Supplement: Supplementary file 1 [file cir-143-1109-s001.pdf]

SUPPLEMENTAL MATERIAL

Contents

ODYSSEY OUTCOMES committees and investigators 2

Table I Standardized mean differences of factors used in propensity score matching 24

Table II Protocol-specified adjustments of alirocumab dose 25

Figure I Consort Diagram. From Schwartz GG, Steg PG, Szarek M, et al. Alirocumab and cardiovascular outcomes after acute coronary syndrome. *N Engl J Med* 379(22):2097-2107.12 Copyright © (2018) Massachusetts Medical Society. Reprinted with permission. 26

Figure II Kaplan-Meier plots of cumulative MACE in strata of achieved LDL-C in the alirocumab group and in propensity score-matched patients from the placebo group. 27

ODYSSEY OUTCOMES committees and investigators

Executive Steering Committee

Gregory G. Schwartz and Ph. Gabriel Steg (Co-Chairs)
Deepak L. Bhatt, Vera A. Bittner, Rafael Diaz, Shaun G. Goodman, Robert A. Harrington, J. Wouter Jukema, Michael Szarek, Harvey D. White, Andreas M. Zeiher.

Nonvoting members:
Ex officio: Pierluigi Tricoci, Matthew T. Roe, Kenneth W. Mahaffey.

Sponsor representatives: Jay M. Edelberg, Corinne Hanotin, Guillaume Lecorps, Angèle Moryusef, Robert Pordy, William J. Sasiela, Jean-François Tamby.

National Leaders

Argentina: Rafael Diaz (Estudios Cardiológicos Latinoamérica, Rosario); **Australia**: Philip E. Aylward (South Australian Health and Medical Research Institute, Flinders University and Medical Centre, Adelaide); **Austria**: Heinz Drexel (Landeskrankenhaus Feldkirch, Feldkirch); **Belgium**: Peter Sinnaeve (UZ Leuven, Leuven); **Bosnia and Herzegovina**: Mirza Dilic (University Clinical Center Sarajevo, Sarajevo); **Brazil**: Renato D. Lopes (Duke University Medical Center, Duke Clinical Research Institute, Durham, NC); **Bulgaria**: Nina N Gotcheva (MHAT "National Cardiology Hospital" EAD, Sofia); **Canada**: Shaun G. Goodman (Canadian VIGOUR Centre, University of Alberta, Edmonton); **Chile**: Juan-Carlos Prieto (Hospital Clinico Universidad De Chile, Santiago); **China**: Huo Yong (Peking University First Hospital, Beijing); **Colombia**: Patricio López-Jaramillo (Fundación Oftalmológica de Santander [FOSCAL], Floridablanca); **Croatia**: Ivan Pećin (University of Zagreb, Zagreb school of medicine, University hospital center Zagreb; Zagreb); Zeljko Reiner (University Hospital Center Zagreb, School of Medicine, University of Zagreb, Zagreb); **Czech Republic**: Petr Ostadal (Na Homolce Hospital, Prague); **Denmark**: Steen Hvitfeldt Poulsen (Aarhus Universitetshospital Skejby, Aarhus N); **Estonia**: Margus Viigimaa (North Estonia Medical Centre, Tallinn); **Finland**: Markku S Nieminen (Division of Cardiology, Heart and Lung Center, HUCH); **France**: Nicolas Danchin (Hôpital Européen Georges Pompidou, FACT, an F-CRIN network, Paris); **Georgia**: Vakhtang Chumburidze (Chapidze Emergency Cardiology Center, Tbilisi); **Germany**: Nikolaus Marx (Universitätsklinikum Aachen, Aachen); **Greece**: Evangelos Liberopoulos (University General Hospital of Ioannina, Ioannina); **Guatemala**: Pablo Carlos Montenegro Valdovinos (Clínica privada, Unidad de Diagnóstico Cardiológico, Guatemala); **Hong Kong**: Hung-Fat Tse (Queen Mary Hospital, The University of Hong Kong, Hong Kong); **Hungary**: Robert Gabor Kiss (Magyar Honvédség Egészségügyi Központ, Budapest); **India**: Denis Xavier (St John's Medical College, Bangalore); **Israel**: Doron Zahger (Soroka University Medical Center, Faculty of Health Sciences, Ben Gurion University of the Negev, Beer Sheva); **Italy**: Marco Valgimigli (Universitaria di Ferrara, Unit Operativa di Cardiologia, Ferrara); **Japan**: Takeshi Kimura (Kyoto University Graduate School of Medicine, Kyoto-shi, Kyoto); **Korea**: Hyo Soo Kim (Seoul National University Hospital, Seoul, Republic of Korea); **Sang-Hyun Kim** (SMG Seoul National University Boramae Medical Center, Seoul); **Latvia**: Andrejs Erglis (Pauls Stradins Clinical University Hospital, University of Latvia, Riga); **Lithuania**: Aleksandras Laucevicius (Vilnius University Hospital Santaros klinikos, Vilnius). **Macedonia**: Sasko Kedev (University Clinic of Cardiology, Skopje); **Malaysia**: Khalid Yusoff (Universiti Teknologi MARA (UiTM) Selayang Campus, Batu Caves and UCSI University); **Mexico**: Gabriel Arturo Ramos López (Medical Office, Guadalajara, Jalisco); **Netherlands**: Marco Alings (Amphia Ziekenhuis Molengracht, Breda); **New Zealand**: Harvey D. White (Green Lane Cardiovascular Service, Auckland City Hospital, Auckland); **Norway**: Sigrun Halvorsen (Oslo Universitetssykehus HF, Oslo); **Peru**: Roger M Correa Flores (Hospital Nacional IV Alberto Sabogal Sologuren, Callao); **Philippines**: Rody G. Sy (Cardinal Santos Medical Center, San Juan); **Poland**: Andrzej Budaj (Postgraduate Medical School, Grochowski Hospital, Warsaw); **Portugal**: Joao Morais (Centro Hospitalar Leiria Pombal, Leiria); **Romania**: Maria Dorobantu (Clinical Emergency Hospial of Bucharest, Bucharest); **Russian Federation**: Yuri Karpov (Russian Cardiological Scientific-Productive Complex, Moscow); **Serbia**: Arsen D. Ristic (Clinical Centre of Serbia, Belgrade); **Singapore**: Terrance Chua (National Heart Centre, Singapore); **Slovakia**: Jan Murin (I. Interna klinika, Univerzitna nemocnica Bratislava, Bratislava); **Slovenia**: Zlatko Fras (Preventive

Cardiology Unit, Department of Vascular Medicine, Division of Medicine, University Medical Centre Ljubljana, Ljubljana; Faculty of Medicine, University of Ljubljana, Ljubljana); **Republic of South Africa:** Anthony J Dalby (Milpark Hospital, Johannesburg); **Spain**: José Tuñón (Fundación Jiménez Díaz, Madrid); **Sri Lanka**: H. Asita de Silva (Clinical Trials Unit, University of Kelaniya); **Sweden**: Emil Hagström (Department of Medical Sciences, and Uppsala Clinical Research Centre, Uppsala University, Uppsala); **Switzerland**: Ulf Landmesser,Universitätsspital Zürich and Christian Müller (Universitätsspital Basel, Basel); **Taiwan**: Chern-En Chiang (General Clinical Research Center, Taipei Veterans General Hospital and National Yang-Ming University, Taipei); **Thailand**: Piyamitr Sritara (Ramathibodi Hospital, Bangkok); **Turkey**: Sema Guneri (Dokuz Eylul Universitesi Tip Fakultesi, Izmir); **Ukraine**: Alexander Parkhomenko ("M.D. Strazhesko Institute of Cardiology of AMS of Ukraine", Kiev); **United Kingdom**: Kausik K. Ray (Imperial College London, London); **United States**: Patrick M. Moriarty (Clinical Pharmacology – University of Kansas Medical Center, Kansas City, Kansas), Matthew T. Roe (Duke Clinical Research Institute, Durham, NC), Robert Vogel (University of Colorado Denver, Denver, Colorado).

Data Safety Monitoring Board

Bernard Chaitman (Chair) (Saint Louis University Center for Health Outcomes Research, Saint Louis University, St. Louis, MO, USA), Sheryl F. Kelsey (Graduate School of Public Health, University of Pittsburgh, PA), Anders G. Olsson (Department of Medicine and Health, Linköping University, Linköping, Sweden), Jean-Lucien Rouleau (Institut de Cardiologie, Université de Montréal, Montréal, Quebec, Canada), Maarten L. Simoons (Erasmus MC, Rotterdam, The Netherlands).

Monitoring of Safety in Patients with Low Low-Density Lipoprotein Values

Karen Alexander (Duke University, Duke Clinical Research Institute, Durham, NC, USA), Chiara Meloni (Duke Clinical Research Institute, Duke University Medical Center, Durham, NC, USA), Robert Rosenson (Mt. Sinai School of Medicine, New York, NY, USA), Eric J.G. Sijbrands (Erasmus MC, Rotterdam, The Netherlands)

Clinical Events Committee

Pierluigi Tricoci (Chair) (Duke University, Durham, NC, USA), John H. Alexander (Duke University, Durham, NC, USA), Luciana Armaganijan (Brazilian Clinical Research Institute, São Paulo, Brazil), Akshay Bagai (St. Michael's Hospital, University of Toronto, Toronto, Canada), Maria Cecilia Bahit (Brazilian Clinical Research Institute, São Paulo, Brazil), J. Matthew Brennan (Duke University, Durham, NC, USA), Shaun Clifton (Duke University, Durham, NC, USA), Adam D. DeVore (Duke University, Durham, NC, USA), Shalonda Deloatch (Duke University, Durham, NC, USA), Sheila Dickey (Duke University, Durham, NC, USA), Keith Dombrowski (Duke University, Durham, NC, USA), Grégory Ducrocq (Hôpital Bichat, Assistance Publique – Hôpitaux de Paris, Paris, France), Zubin Eapen (Duke University, Durham, NC, USA), Patricia Endsley (Duke University, Durham, NC, USA), Arleen Eppinger (Duke University, Durham, NC, USA), Robert W. Harrison (Duke University, Durham, NC, USA), Connie Ng Hess (University of Colorado Denver, Denver, CO, USA), Mark A. Hlatky (Stanford University, Stanford, CA, USA), Joseph Dedrick Jordan (University of North Carolina, NC, USA), Joshua W. Knowles (Stanford University, Stanford, CA, USA), Bradley J. Kolls (Duke University, Durham, NC, USA), David F. Kong (Duke University, Durham, NC, USA), Sergio Leonardi (Fondazione IRCCS Policlinico S. Matteo, Pavia, Italy), Linda Lillis (Duke University, Durham, NC, USA), Renato D. Lopes (Duke University, Durham, NC, USA), David J. Maron (Stanford University, Stanford, CA, USA), Kenneth W. Mahaffey (Stanford University, Stanford, CA, USA), Jill Marcus (Duke University, Durham, NC, USA), Robin Mathews (Duke University, Durham, NC, USA), Rajendra H. Mehta (Duke University, Durham, NC, USA), Robert J. Mentz (Duke University, Durham, NC, USA), Humberto Graner Moreira (Brazilian Clinical Research Institute, São Paulo, Brazil), Chetan B. Patel (Duke University, Durham, NC, USA), Sabrina Bernardez Pereira (Brazilian Clinical Research Institute, São Paulo, Brazil), Lynn Perkins (Duke University, Durham, NC, USA), Thomas J. Povsic (Duke University, Durham, NC, USA), Etienne Puymirat (European Hospital Georges Pompidou, Paris, France), Matthew T. Roe (Duke University, Durham, NC, USA), William Schuyler Jones (Duke University, Durham, NC, USA), Bimal R. Shah (Duke University, Durham, NC, USA), Matthew W. Sherwood (Duke University, Durham, NC, USA), Kenya Stringfellow

(Duke University, Durham, NC, USA), Darin Sujjavanich (Duke University, Durham, NC, USA), Mustafa Toma (St. Paul's Hospital, University of British Columbia, Vancouver, BC, Canada), Charlene Trotter (Duke University, Durham, NC, USA), Sean F.P. van Diepen (Canadian VIGOUR Centre, University of Alberta, Edmonton, Alberta, Canada), Matthew D. Wilson (Duke University, Durham, NC, USA), Andrew Tze-Kay Yan (St. Michael’s Hospital, University of Toronto, Toronto, Canada).

Health Economics Steering Committee

To be added

Investigators who enrolled at least 1 patient (in descending order of number of patients enrolled) (18,927 patients enrolled overall)

Argentina (592 patients enrolled) Lilia B Schiavi (Clinica Del Prado, Cordoba, Córdoba); Marcelo Garrido (Clinica Privada Provincial, Merlo, Buenos Aires Province); Andrés F Alvarisqueta (Centro de Investigaciones Medicas Mar del Plata, Mar del Plata, Buenos Aires Province); Sonia A Sassone (Consultorios Asoc. de Endocrinologia e Inv. Clin. Aplicada, CABA, Buenos Aires); Anselmo P Bordonava (Clinica FUSAVIM Privada, Villa Maria, Córdoba); Alberto E Alves De Lima (Instituto Cardiovascular De Buenos Aires, Ciudad de Buenos Aires, Buenos Aires); Jorge M Schmidberg (Instituto Medico Aguero, Moron, Buenos Aires); Ernesto A Duronto (Fundacion Favaloro para la Docencia e Investigacion Médica, CABA, Buenos Aires); Orlando C Caruso (Hospital Central Mendoza, Mendoza, Mendoza); Leonardo P Novaretto (Sanatorio Santa Rosa, Santa Rosa, La Pampa); Miguel Angel Hominal (Centro de Investigaciones Clinicas del litoral, Santa Fe, Santa Fe); Oscar R Montaña (Dim Clinica Privada, Ramos Mejia, Buenos Aires Province); Alberto Caccavo (Clinica Coronel Suarez, Coronel Suarez, Buenos Aires Province); Oscar A Gomez Vilamajo (Sanatorio San Martin SA, Venado Tuerto, Santa Fe); Alberto J Lorenzatti (Instituto Medico DAMIC, Córdoba, Córdoba); Luis R Cartasegna (Hospital Italiano De La Plata, La Plata, Buenos Aires Province); Gustavo A Paterlini (Investigaciones Clinicas Tucuman, San Miguel De Tucuman, Tucumán); Ignacio J Mackinnon (Instituto De Investigaciones Clínicas, Mar Del Plata, Buenos Aires Province); Guillermo D Caime (Instituto de Diagnostico Cardiovascular La Plata, La Plata, Buenos Aires Province); Marcos Amuchastegui and Oscar Salomone (Hospital Privado Centro Medico De Cordoba, Cordoba, Córdoba); Oscar R Codutti (Cordis Instituto del Corazón, Resistencia, Chaco); Horacio O Jure (Clinica Chutro S.R.L, Cordoba, Córdoba); Julio OE Bono (Sanatorio Allende, Córdoba, Córdoba); Adrian D Hrabar (Instituto de Investigaciones Clinicas Quilmes, Quilmes, Buenos Aires Province); Julio A Vallejos (Instituto De Cardiología De Corrientes, Corrientes, Corrientes); Rodolfo A Ahuad Guerrero (Corporacion Medica San Martin, San Martin, Buenos Aires Province); Federico Novoa (Consultorio Privado, San Isidro, Buenos Aires); Cristian A Patocchi (Hospital Regional Español Bahia Blanca, Bahia Blanca, Buenos Aires Province); Cesar J Zaidman (Centro de Investigacion y Prevencion Cardiovascular, CABA, Buenos Aires); Maria E Giuliano (Instituto Medico de la Fundacion de Estudios Clinicos, Rosario, Santa Fe); Ricardo D Dran (Sanatorio Mariano Pelliza, Olivos, Buenos Aires Province); Marisa L Vico (Instituto de Investigaciones Clinicas de Zarate, Zarate, Buenos Aires Province); Gabriela S Carnero (I.M.E.P. Instituto Médico Elsa Perez, Ciudadela, Buenos Aires Province); Pablo N Guzman (Sanatorio San Francisco, Santiago del Estero, Santiago del Estero); Juan C Medrano Allende (Clínica y Maternidad Suizo Argentina, CABA, Buenos Aires); Daniela F Garcia Brasca (Hospital Italiano, Cordoba, Córdoba); Miguel H Bustamante Labarta (Centro Médico Lebensohn, Junin, Buenos Aires Province); Sebastian Nani (Clinica Olivos, Olivos, Buenos Aires Province); Eduardo DS Blumberg (Medeos, CABA, Buenos Aires); Hugo R Colombo (Clinica Colombo, Cordoba, Córdoba); Alberto Liberman and Victorino Fuentealba (Instituto Privado de Investigaciones Clinicas de Cordoba, Cordoba, Córdoba); Hector L Luciardi (Centro Modelo De Cardiología, San Miguel de Tucuman, Tucumán); Gabriel D Waisman (Hospital Italiano De Buenos Aires, Buenos Aires, Buenos Aires); Mario A Berli (Hospital "provincial" Dr Jose Maria Cullen, Santa Fe, Santa Fe); Ruben O Garcia Duran (Instituto de Investigaciones Clínicas San Nicolás, San Nicolas, Buenos Aires Province); Horacio G Cestari (Sanatorio Guemes, CABA, Buenos Aires); Hugo A Luquez (Centro Medico Luquez, Cordoba, Cordoba); Jorge A Giordano (Clinica Instituto Medico Adrogue, Adrogue, Buenos Aires Province); Silvia S Saavedra (Sanatorio Parque S.A., Salta, Salta); Gerardo Zapata (Instituto Cardiovascular de Rosario, Rosario, Santa Fe); Osvaldo Costamagna (Clinica Parra Centro de Investigaciones Clinicas de Rafaela, Rafaela, Santa Fe); Susana Llois (Hospital Interzonal [General] de Agudos Eva Peron, San Martin, Buenos Aires Province).

Australia (216 patients enrolled) Jonathon H Waites (Coffs Harbour Health Campus, Coffs Harbour, New South Wales); Nicholas Collins (John Hunter Hospital, New Lambton Heights, New South Wales); Allan Soward (Mildura Cardiology, Mildura, Victoria); Philip E Aylward (South Australian Health and Medical Research Institute, Flinders University and Medical Centre, Adelaide South Australia); Chris LS Hii (Calvary Health Care, Bruce, Australian Capital Territory); Philip E Aylward (Heart and Vascular Institute, Fullarton, South Australia); James Shaw (The Alfred Hospital, Prahran, Victoria); Margaret A Arstall (Lyell McEwin Hospital Elizabeth Vale, South Australia); John Horowitz and Daniel Ninio (The Queen Elizabeth Hospital, Woodville South, South Australia); James F Rogers (Gosford Hospital, Gosford, New South Wales); David Colquhoun (Core Research Group Pty Ltd, Milton, Queensland); Romulo E Oqueli Flores (Ballarat Health Services, Ballarat, Victoria); Philip Roberts-Thomson (Royal Hobart Hospital, Hobart, Tasmania); Owen Raffel (Prince Charles Hospital, Chermside, Queensland); Sam J Lehman (Adelaide Medical Research, Ashford, South Australia); Constantine Aroney (Holy Spirit Northside Hospital, Chermside, Queensland); Steven GM Coverdale (Nambour General Hospital, Nambour, Queensland); Paul J Garrahy (Princess Alexandra Hospital, Woolloongabba, Queensland); Gregory Starmer (The Cairns Hospital, Cairns, Queensland); Mark Sader (St George Hospital, Kogarah, New South Wales); Patrick A Carroll (Redcliffe Hospital, Redcliffe, Queensland); Ronald Dick (Epworth Healthcare, Richmond, Victoria).

Austria (58 patients enrolled) Robert Zweiker (Universitätsklinikum Graz, Graz); Uta Hoppe (Universitaetsklinikum Salzburg, Salzburg); Heinz Drexel (Vorarlberg Institut für Vaskuläre Forschung (VIVIT), Feldkirch); Kurt Huber (Wilhelminenspital der Stadt Wien, Wien); Rudolf Berger and Georg Delle-Karth and Bernhard Frey (Universitätsklinik für Innere Medizin II Währinger Gürtel 18-20; Franz Weidinger (Krankenanstalt Rudolfstiftung der Stadt Wien, Wien).

Belgium (197 patients enrolled) Dirk Faes (Mariaziekenhuis vzw, Overpelt); Kurt Hermans (AZ St. Lucas, Gent); Bruno Pirenne (Clinique Saint-Pierre, Ottignies); Attilio Leone (CHU Tivoli, La Louviere); Etienne Hoffer (CHR De La Citadelle, Liège); Peter Sinnaeve (UZ Leuven, Leuven); Mathias CM Vrolix (ZOL, Genk); Luc De Wolf (Private Practice, Tienen); Bart Wollaert (ZNA Stuivenberg, Antwerpen); Marc Castadot (Clinique Saint Jean, Bruxelles); Karl Dujardin (AZ Delta, Roeselare); Christophe Beauloye (UCL Saint Luc, Bruxelles); Geert Vervoort (AZ St.Maarten, Mechelen); Harry Striekwold (Heilig Hart Ziekenhuis, Mol); Carl Convens (ZNA Middelheim, Antwerpen); John Roosen (Imelda Hospital, Bonheiden); Emanuele Barbato (O.L Vrouwziekenhuis Campus Aalst, Aalst); Marc Claeys (UZ Antwerpen, Edegem); Frank Cools (AZ Klina, Brasschaat).

Bosnia and Herzegovina (156 patients enrolled) Ibrahim Terzic (Heart Center BH, Tuzla); Fahir Barakovic (University Medical Center Tuzla, Tuzla); Zlatko Midzic (Cantonal Hospital "Dr. Irfan Ljubijankic" Bihac, Bihac); Belma Pojskic (Cantonal Hospital Zenica, Zenica); Emir Fazlibegovic (University Clinical Hospital Mostar, Mostar); Mirza Dilic (University Clinical Center Sarajevo, Sarajevo); Mehmed Kulić (University Clinical Center of Sarajevo, Sarajevo); Azra Durak-Nalbantic and Mehmed Kulic (University Clinical Center of Sarajevo, Sarajevo); Dusko Vulic (Clinical Center Banja Luka, Banja Luka); Adis Muslibegovic (Regional Medical Center „Dr. Safet Mujic', Mostar); Boris Goronja (Clinical Center Banja Luka, Banja Luka).

Brazil (928 patients enrolled) Gilmar Reis (CARDRESEARCH - Cardiologia Assistencial e de Pesquisa, Belo Horizonte, Minas Gerais); Luciano Sousa (Hospital do Coracao do Brasil, Brasilia, Distrito Federal); Jose C Nicolau (INCOR - Instituto do Coracao, Sao Paulo, Sao Paulo); Flavio E Giorgeto (Hospital Sao Francisco De Assis, Belo Horizonte, Minas Gerais); Ricardo P Silva (Hospital Universitário Walter Cantídio, Fortaleza); Lilia Nigro Maia (Hospital De Base de São José do Rio Preto, Sao Jose Do Rio Preto, Sao Paulo); Rafael Rech (Hospital Universitário Ulbra, Canoas); Paulo RF Rossi (Hospital Evangelico De Curitiba, Curitiba, Parana); Maria José AG Cerqueira (Instituto de Ensino e Pesquisa Clínica do Ceara Ltda., Fortaleza, Ceara); Norberto Duda (Hospital São Vicente De Paulo, Passo Fundo, Rio Grande do Sul); Renato Kalil (Instituto De Cardiologia Do Rio Grande Do Sul, Porto Alegre, Rio Grande do Sul); Adrian Kormann (Sociedade Divina Providência (Hospital Santa Isabel), Blumenau, Santa Catarina); José Antonio M Abrantes (Santa Casa de Misericórdia de Pelotas, Pelotas, Rio Grande do Sul); Pedro Pimentel Filho (Hospital Nossa Senhora da Conceicao, Porto Alegre, Rio Grande do Sul); Ana Priscila Soggia (Sociedade Beneficiente de Senhoras Hospital Sírio Libanês, Sao Paulo); Mayler ON de Santos (Centro de Cardiologia e Radiologia Intervencionista LTDA, Aparecida de Goiania); Fernando Neuenschwander (Hospital Vera Cruz - NUPEC, Belo Horizonte); Luiz C Bodanese (Hospital Sao Lucas -

PUCRS, Porto Alegre, Rio Grande do Sul); Yorghos L Michalaros (Instituto de Ensino, Consultoria e Pesquisa Clinica LTDA-ME, Belo Horizonte, Minas Gerais); Freddy G Eliaschewitz (CPCLIN - Centro de Pesquisas Clinicas, Sao Paulo, Sao Paulo); Maria H Vidotti (Clínica LOEMA, Campinas, Sao Paulo); Paulo E Leaes (Santa Casa De Porto Alegre, Porto Alegre, Rio Grande do Sul); Roberto V Botelho (Instituto Do Coracao Do Triangulo, Uberlandia, Minas Gerais); Sergio Kaiser (CCBR Brasil, Rio De Janeiro, Rio de Janeiro); Euler Roberto F Fernandes Manenti (IMV - Instituto De Medicina Vascular Hospital Mae de Deus, Porto Alegre, Rio Grande do Sul); Dalton B Precoma (Sociedade Hospitalar Angelina Caron, Campina Grande do Sul, Parana); Jose C Moura Jorge (Santa Casa De Curitiba, Curitiba, Parana); Pedro GM de B Silva (Esho Empresa de Serviço Hospitalares S.A, Sao Paulo, Sao Paulo); Jose A Silveira (Faculdade de Medicina do ABC / CEMEC, Sao Bernardo do Campo, Sao Paulo); Wladmir Saporito (Instituto de Moléstias Cardiovasculares Tatuí (IMC), Tatuí, Sao Paulo); Jose A Marin Neto (HCFMRP, Riberao Preto); Gilson S Feitosa (Hospital Santa Izabel / Santa Casa de Misericordia da Bahia, Salvador, Bahia); Luiz Eduardo F Ritt (Hospital Cardio Pulmonar, Salvador; Hospital Cardio Pulmonar at Salvador); Juliana A de Souza (Centro de Cardiologia Clinica e Pesquisa Dra. Juliana Souza, Brasilia, Distrito Federal); Fernando Costa (FGM-Clinica Paulista de Doencas Cardiovasculares, Sao Paulo); Weimar KSB Souza (Barroso e Sebba Ltda, Goiania); Helder JL Reis (Hospital De Clinicas Gaspar Vianna, Belem, Para); Renato D. Lopes (Universidade Federal de São Paulo - UNIFESP/EPM); Wladimir Saporito and Leandro Machado (Hospital Estadual Mario Covas, Santo Andre); José Carlos Aidar Ayoub (Instituto de Moléstias Cardiovasculares/IMC, São José do Rio Preto).

Bulgaria (333 patients enrolled) Georgi V Todorov (2nd MHAT, Sofia); Fedya P Nikolov (Univ Multiprofile Hosp for Active Treatment "Sveti Georgi", Plovdiv); Elena S Velcheva (Specialized Hospital for Active Treatment in Cardiology, Pleven); Maria L Tzekova (UMHAT dr Georgi Stranski, Pleven); Haralambi O Benov (Multiprof Distr Hosp for Active Treatment "Dr.St.Cherkezov", Veliko Tarnovo); Stanislav L Petranov (Multiprofile Hospital for Active Treatment - Burgas AD, Burgas); Haralin S Tumbev (Specialized Hosp. for Active Cardio. Treatment–“Cardiolife”, Varna); Nina S Shehova-Yankova (MHAT Bratan Shukerov AD, Cardio. Dept. with Inrtensive Unite, Smoljan); Dimitar T Markov (UMHAT Tzaritza Yoanna – ISUL EAD, Sofia); Dimitar H Raev (MHAT 'Sveti Vrach' EOOD, Sandanski); Mihail N Mollov (First Multiprofile Hospital for Active Treatment - Sofia EAD, Sofia); Kostadin N Kichukov ("MHAT Lyulin" EAD. Department of Internal Diseases, Sofia); Katya A Ilieva-Pandeva (MHAT Blagoevgrad AD, Blagoevgrad); Nina N Gotcheva (MHAT “National Cardiology Hospital” EAD, Sofia); Raya Ivanova and Maryana Gospodinov (UMHAT Alexandrovska EAD, Sofia); Valentina M Mincheva (NMT Hospital "Tsar Boris III", Sofia); Petar V Lazov (Multiprofile Hospital for Active Treatment - Pazardzhik AD, Pazardzhik); Bojidar I Dimov (Fifth MHAT-Sofia EAD, Sofia).

Canada (361 patients enrolled) Manohara Senaratne (Dr. M. P. J Senaratne Professional Corporation, Edmonton, Alberta); James Stone (TotalCardiology, Calgary, Alberta); Jan Kornder (deceased), Stephen Pearce (Surrey Memorial Hospital, Surrey, British Columbia); Danielle Dion (Centre Hospitalier Beauce-Etchemin, St. Georges de Beauce, Quebec); Daniel Savard (CardioVasc HR, St. Jean sur Richelieu, Quebec); Yves Pesant (Saint Jerome Medical Research, Inc., St. Jerome); Amritanshu Pandey (Cambridge Cardiac Care Center, Cambridge, Ontario); Simon Robinson (Victoria Heart Institute Foundation, Victoria, British Columbia); Gilbert Gosselin (Centre Hospitalier Pierre Le Gardeur, Terrebonne, Quebec); Saul Vizel (Vizel Cardiac Research, Cambridge, Ontario); Gordon Hoag (Discovery Clinical Services, Victoria, British Columbia); Ronald Bourgeois (G.A. Research Associates, Ltd., Moncton, New Brunswick); Anne Morisset (Centre de Santé et de Services Sociaux de la Haute-Yamaska, Granby, Quebec); Eric Sabbah (Centre de Depistage et Recherche Cardiovasculaire Rive-Sud, Longueuil, Quebec); Bruce Sussex (General Hospital Health Sciences Center, St. John’s, Newfoundland and Labrador); Simon Kouz (Centre Hospitalier Régional de Lanaudière, Joliette, Quebec); Paul MacDonald (Cape Breton Regional Hospital, Sydney, Nova Scotia); Ariel Diaz (Centre Hospitalier Régional de Trois-Rivières, Trois-Rivières, Quebec); Nicolas Michaud (Clinique de Cardiologie de Lévis, Lévis, Quebec); David Fell (Newmarket Cardiology Research Group, Newmarket, Ontario); Raymond Leung and Raymond Leung Royal Alexandra Hospital, Edmonton, Alberta); Tycho Vuurmans (Royal Columbian Hospital, New Westminster, British Columbia); Christopher Lai, Frank Nigro (current PI) (Thunder Bay Regional Health Science Center, Thunder Bay, Ontario); Richard Davies (Ottawa Heart Institute, Ottawa, Ontario); Gustavo Nogareda (Red Deer Regional Hospital Centre, Red Deer, Alberta); Ram Vijayaraghavan (Scarborough Cardiology Research, Scarborough, Ontario); John Ducas (St. Boniface Hospital, Winnipeg, Manitoba); Serge Lepage (Hôtel-Dieu du Centre Hospitalier Universitaire de

Sherbrooke, Sherbrooke, Quebec); Shamir Mehta (Hamilton General Hospital, Hamilton, Ontario); James Cha (Private Practice affiliated Lakeridge Health of Oshawa, Oshawa, Ontario); Robert Dupuis (Centre de Santé et de Services Sociaux de la Région de Thetford (Recherche), Thetford Mines, Quebec); Peter Fong and Sohrab Lutchmedial (Saint John Regional Hospital, Saint John, New Brunswick); Josep Rodes-Cabau (Quebec Heart Institute, Laval Hospital, Sainte-Foy, Quebec City, Quebec); Hussein Fadlallah (Recherche GCP Research, Montreal, Quebec); David Cleveland (Penticton Regional Hospital, Penticton, British Columbia); Thao Huynh (Montreal General Hospital, Montreal, Quebec); Iqbal Bata (Queen Elizabeth II Health Sciences Center, Halifax, Nova Scotia); Adnan Hameed (Medical Professional Corporation, St. Catherines, Ontario).

Chile (132 patients enrolled) Cristian Pincetti (Centro de Investigacion Clinica del Sur Cics, Temuco, IX Region de la Araucanía); Sergio Potthoff (Corporacion de Beneficencia Osorno, Osorno, X Region los Lagos); Juan C Prieto (Hospital Clinico Universidad De Chile, Santiago); Monica Acevedo (Centro de Investigaciones Clínicas de la UniversidadCatólica, Santiago, RM Region Metropolitan); Arnoldo Aguirre (Hospital Barros Luco Trudeau, Santiago, RM Region Metropolitan); Margarita Vejar (Hospital del Salvador, Santiago); Mario Yañez (Hospital Naval Almirante Nef de Viña del Mar, Viña Del Mar, V Region de Valparaiso); Guillermo Araneda (Hospital Clinico Magallanes, Punta Arenas, XII Region Magallanes); Mauricio Fernandez (Clinica Alemana de Santiago, Santiago, RM Region Metropolitan); Luis Perez (Hospital Clinico Regional Dr. Guillermo Grant Benavente, Concepcion, VIII Region del Bio-Bio); Paola Varleta (Psicomedica Clinical and Research Group, Santiago, RM Region Metropolitan); Fernando Florenzano (Biomedica Research Group, Santiago, RM Region Metropolitan); Laura Huidobro (Clinica Universidad Catolica del Maule, Talca, VII Region del Maule); Carlos A Raffo (Estudios clinicos V Region Ltda, Viña del Mar); Claudia Olivares (Hospital y CRS El Pino, Santiago, RM Region Metropolitan); Leonardo Nahuelpan (Hospital Base Valdivia, Valdivia XIV, Region de los Rios); Humberto Montecinos (Complejo Asistencial Dr. Sotero del Rio, Santiago).

China (614 patients enrolled) Jiyan Chen (Guangdong General Hospital, Guangzhou); Yugang Dong (The First Affiliated Hospital, Sun Yat-Sen University, Guangzhou); Weijian Huang (The First Affiliated Hospital of Wenzhou Medical University, Wenzhou); Jianzhong Wang (Siping Central People's Hospital, Siping); Shi'An Huang (Affiliated Hospital of Guangdong Medical University, Zhanjiang); Zhuhua Yao (Tianjin Union Medical Center, Tianjin); Xiang Li and Lan Cui (Yanbian University Hospital, Yanji); Wenhua Lin (TEDA International Cardiovascular Hospital, Tianjin); Yuemin Sun (Tianjin Medical University General Hospital,); Jingfeng Wang (Sun Yat-Sen Memorial Hospital, Sun Yat-Sen University, Guangzhou); Jianping Li (Peking University First Hospital, Beijing); Xuelian Zhang (People's Hospital, Changchun); Hong Zhu (The Affiliated Hospital of Xuzhou Medical College, Xuzhou); Dandan Chen (Guizhou Provincial People’s Hospital, Guiyang); Lan Huang (Xinqiao Hospital of Third Military Medical University, Chongqing); Shaohong Dong (Shenzhen People’s Hospital, Shenzhen); Guohai Su (Jinan Central Hospital Affiliated to Shandong University, Jinan); Biao Xu (Nanjing Drum Tower Hospital, The Affiliated Hospital of NJMU, Nanjing); Xi Su (Wuhan Asian Heart Hospital, Wuhan); Xiaoshu Cheng (The Second Affiliated Hospital to Nanchang University, Nanchang); Jinxiu Lin (The First Affiliated Hospital of Fujian Medical University, Fuzhou); Wenxia Zong (The Third People’s Hospital of Hubei Province, Wuhan); Huanming Li (Tianjin 4th Centre Hospital, Tianjin); Yi Feng (Zhongda Hospital, Southeast University, Nanjing); Dingli Xu (Nanfang hospital, Southern Medical University, Guangzhou); Xinchun Yang (Beijing Chaoyang Hospital, Beijing); Yuannan Ke (China-Japan Friendship Hospital, Beijing); Xuefeng Lin (The First Affiliated Hospital of Baotou Medical College, Baotou); Zheng Zhang (The First Hospital of Lanzhou University, Lanzhou); Zeqi Zheng (The First Affiliated Hospital of Nanchang University, Nanchang); Zhurong Luo (Fuzhou General Hospital of Nanjing Military Command, Fuzhou); Yundai Chen (Chinese PLA General Hospital, Beijing); Chunhua Ding and Yi Zhong (The Central Hospital of China Aerospace Corporation, Beijing); Yang Zheng (First Hospital of Jilin University, Changchun); Xiaodong Li (Shengjing Hospital of China Medical University, Shenyang); Daoquan Peng and Shuiping Zhao (The Second Xiangya Hospital of Central South University, Changsha); Ying Li and Xuebo Liu (Shanghai East Hospital, Shanghai); Meng Wei (Shanghai Sixth People's Hospital, Shanghai); Shaowen Liu (Shanghai First People's Hospital, Shanghai); Yihua Yu (Zhejiang Hospital, Hangzhou); Baiming Qu (Zhejiang Provincial People’s Hospital, Hangzhou); Weihong Jiang (The Third Xiangya Hospital Of Central South University, Changsha); Yujie Zhou (Beijing Anzhen Hospital, Capital Medical University, Beijing); Xingsheng Zhao (Inner Mongolia People's Hospital, Hohhot); Zuyi Yuan (The First Affiliated Hospital of Xi'an JiaoTong University, Xi'An); Ying Guo (Hunan Provincial People's Hospital, Changsha);

Xiping Xu (The First People's Hospital of Yueyang, Yueyang); Xubo Shi (Beijing Tongren Hospital, Capital Medical University, Beijing); Junbo Ge (Zhongshan Hospital Fudan University, Shanghai); Guosheng Fu (Sir Run Run Shaw Hospital of Zhejiang University, Hangzhou); Feng Bai (Lanzhou University Second Hospital, Lanzhou); Weiyi Fang (Shanghai Chest Hospital Shanghai Jiaotong University, Shanghai); Xiling Shou (Shaanxi Provincial People's Hospital, Xi'an); Xiangjun Yang (First Affiliated Hospital Of Soochow University, Suzhou); Jian'An Wang and Meixiang Xiang (The Second Affiliated Hospital of Zhejiang University, Hangzhou); Yingxian Sun (First Hospital of China Medical University, Shenyang); Qinghua Lu (Second Hospital of Shandong University, Jinan); Ruiyan Zhang (Ruijin Hospital Affiliated to Shanghai Jiaotong University, Shanghai); Jianhua Zhu (First Affiliated Hospital of Zhejiang University, Hangzhou); Yizhou Xu (Hangzhou First People's Hospital, Hangzhou); Zhongcai Fan (Affiliated Hospital of Luzhou Medical College, Luzhou); Tianchang Li (Navy General Hospital, Cardiology Center, Beijing); Chun Wu (Peking University Shenzhen Hospital, Shenzhen).

Colombia (354 patients enrolled) Nicolas Jaramillo (Centro de Medicina del Ejercicio y Rehabilitacion Cardiaca, Medellin); Gregorio Sanchez Vallejo (Fundación Cardiomet CEQUIN, Armenia, Quindio); Diana C Luna Botia (Hospital Santa Clara, Bogota); Rodrigo Botero Lopez (IPS Rodrigo Botero SAS, Medellin); Dora I Molina De Salazar (Asociacion IPS Medicos Internistas de Manizales, Caldas); Alberto J Cadena Bonfanti and Carlos Cotes Aroca (Clinica de la Costa ltda, Barranquilla); Juan Diego Higuera and Marco Blanquicett (Fundación Oftalmológica FOSCAL,); Sandra I Barrera Silva (Caja de Compensacion Familiar CAFAM, Bogota); Henry J Garcia Lozada (Fundacion Centro de Investigaciones Biomedicas Riescard, Espinal - Tolima); Julian A Coronel Arroyo (IPS Centro Medico Julian Coronel S.A., Cali, Valle); Jose L Accini Mendoza (IPS Centro cientifico asistencial SAS, Barranquilla); Ricardo L Fernandez Ruiz and Alvaro M. Quintero Ossa (Centro Cardiovascular Colombiano Clinica Santa Maria CARDIOVID Medellin, Antioquia); Fernando G Manzur Jatin (Centro de diagnostico cardiologico, Cartagena); Aristides Sotomayor Herazo (Centro Cardiovascular Santa Lucia, Cartagena, Bolivar); Jeffrey Castellanos Parada and Rafael Suarez Arambula (SIMEDICS IPS S.A.S, Bogota D.C.); Miguel A Urina Triana (Fundacion del Caribe para la Investigacion Biomedica, Barranquilla); Angela M Fernandez Trujillo (Centro Medico Imbanaco de Cali S.A, Cali, Valle).

Croatia (70 patients enrolled) Maja Strozzi (Clinical Hospital Center Zagreb, Zagreb); Siniša Car (General Hospital Varazdin, Varazdin); Melita Jerić (General Hospital Varazdin, Varazdin), Davor Miličić (University Hospital Center Zagreb, Zagreb); Martina Lovrić Benčić (Clinical Hospital Center Zagreb, Zagreb); Hrvoje Pintarić (University Hospital Centre „Sestre Milosrdnice“, Zagreb); Đeiti Prvulović (General Hospital Slavonski Brod, Slavonski brod); Jozica Šikić (Clinical Hospital Sveti Duh, Zagreb); Viktor Peršić (Thalassotherapia-Opatija, Opatija); Dean Mileta (Clinical Hospital Merkur, Zagreb); Kresimir Štambuk (Special Hosp. for Med. Rehab.Krapinske Toplice, Krapinske Toplice); Zdravko Babić (Clinical Hospital Center, 'Sestre Milosrdnice', Zagreb); Vjekoslav Tomulic (Clinical Hospital Center Rijeka, Rijeka); Josip Lukenda and Stanka Mejic Krstulovic (General Hospital Dubrovnik, General Hospital Dubrovnik, Dubrovnik); Boris Starcevic (Clinical Hospital Dubrava, Clinical Hospital Dubrava, Zagreb).

Czech Republic (381 patients enrolled) Jindrich Spinar (FN Brno, Brno); David Horak and Zdenek Velicka (Krajska Nemocnice Liberec a.s., Liberec); Josef Stasek (Fakultni Nemocnice Hradec Kralove, Hradec Kralove); David Alan (Fakultni Nemocnice v Motole, Praha); Vilma Machova (Interni a kardiologicka ambulance, InterkardioML, s.r.o., Marianske Lazne); Ales Linhart (Vseobecna Fakultni Nemocnice V Praze, Praha); Vojtech Novotny (Nemocnice Pardubickeho kraje, a.s., Pardubice); Vladimir Kaucak (Mestska Nemocnice Ostrava, Ostrava); Richard Rokyta (Fakultni Nemocnice Plzen, Plzen); Robert Naplava (Centrum pro choroby srdce a cev, Kromeriz); Zdenek Coufal (Krajska Nemocnice Tomase Bati a.s., Zlin); Vera Adamkova (Institut klinicke a experimentalni mediciny, Praha); Ivo Podpera (Oblastni Nemocnice Kladno a.s., Kladno); Jiri Zizka (Thomayerova nemocnice, Praha); Zuzana Motovska (Fakultni Nemocnice Kralovske Vinohrady, Praha); Ivana Marusincova (Kardio Sever, r.o., Ceska Lipa); Premysl Svab (Kardiologicka ambulance, Rakovnik); Petr Ostadal (Nemocnice Na Homolce, Praha); Petr Heinc (Fakultni Nemocnice Olomouc, Olomouc); Jiri Kuchar (Kardiologie s.r.o., Cesky Krumlov); Petr Povolny (Cardiocentrum Kladno s.r.o., P-P klinika Kladno, Kladno); Jiri Matuska (Interni a cevni ambulance, Hodonin).

Denmark (352 patients enrolled) Steen H Poulsen (Aarhus Universitetshospital Skejby, Aarhus N); Bent Raungaard (Dep Cardiology, Aalborg); Peter Clemmensen; Lia E Bang (Rigshospitalet Hjertecentret,

Copenhagen); Ole May; Morten Bøttcher (Region Hospital Herning, Herning); Jens D Hove (Hvidovre Hospital, Hvidovre); Lars Frost (Regionshospitalet Silkeborg, Silkeborg); Gunnar Gislason (Gentofte Hospital, Hellerup); John Larsen (Næstved Sygehus, Næstved);Peter Betton Johansen; Flemming Hald and Peter Johansen (Sygehus Lillebælt, Vejle Sygehus, Vejle); Jørgen Jeppesen (Glostrup Hospital, Glostrup); Tonny Nielsen (Sjællands Universitetshospital Køge, Køge); Kjeld S Kristensen; Piotr Maria Walichiewicz (Holbaek Sygehus, Holbaek); Jens D Lomholdt (Slagelse Sygehus, Slagelse); Ib C Klausen (Regionshospitalet Viborg, Viborg); Peter Kaiser Nielsen (Hillerød Hospital, Hillerød); Flemming Davidsen (Sygehus Sønderjylland, Aabenraa); Lars Videbaek (Odense Universitetshospital, Odense C).

Estonia (216 patients enrolled) Margus Viigimaa (North Estonia Medical Centre, Tallinn); Mai Soots (SA Viljandi Haigla, Viljandimaa); Veiko Vahula (SA Pärnu Haigla, Pärnu); Anu Hedman (AS Ida-Tallinna Keskhaigla, Tallinn); Üllar Soopõld (SA Tartu Ülikooli Kliinikum, Tartu); Kaja Märtsin and Tiina Jurgenson (Mustamäe Tervisekeskus OÜ, Tallinn); Arved Kristjan (Mahe Perearst OU, Tallinn).

Finland (116 patients enrolled) Markku S Nieminen, (Biomedicum and Division of Cardiology, Helsinki University Hospital, Juhani K Airaksinen); Saila Vikman Heart Center, Tampere University Hospital; Heikki Huikuri (University Hospital of Oulu), Juhani Airaksinen (Turun Yliopistollinen sairaala Sydänkeskus)**France (185 patients enrolled)** Pierre Coste (Hôpital Cardiologique du Haut-Leveque, Pessac); Emile Ferrari (Hôpital Pasteur, Nice); Nicolas Danchin (Hôpital Europeen Georges Pompidou, Paris); Olivier Morel (Nouvel Hôpital Civil, Strasbourg); Gilles Montalescot (Hôpital Pitié Salpêtrière, Paris); Jacques Machecourt and Gilles Barone-Rochette (CHU Albert Michallon, Grenoble); Jacques Mansourati (CHU De Brest Hôpital de la Cavale Blanche, Brest); Yves Cottin (CHU de Dijon Le Bocage Central, Dijon); Philippe Gabriel Steg (Hôpital Bichat, Paris); Florence Leclercq (Hôpital Arnaud de Villeneuve, Montpellier); Abdelkader Belhassane (Centre Hospitalier de Cambrai, Cambrai); Nicolas Delarche (Centre Hospitalier Général, Pau); Franck Boccara (Hôpital Saint-Antoine, Paris); Franck Paganelli (Hôpital Nord, Marseille); Jérôme Clerc (CH Compiègne - Noyon, Compiegne); Francois Schiele (CHU Jean Minjoz, Besancon); Victor Aboyans (CHU Dupuytren, Limoges); Vincent Probst (CHU de Nantes, Nantes); Jacques Berland (Clinique Saint Hilaire, Rouen); Thierry Lefèvre (Institut Hospitalier Jacques Cartier, Massy); Bernard Citron (CHU Gabriel Montpied, Clermont-Ferrand).

Georgia (131 patients enrolled) Vakhtang Chumburidze (Chapidze Emergency Cardiology Center, Tbilisi); Irakli Khintibidze ("Aleksandr Aladashvili Clinic" LLC, Tbilisi); Tamaz Shaburishvili (Tbilisi Heart and Vascular Clinic, Ltd, Tbilisi); Zurab Pagava (Center of vascular and heart diseases, Tbilisi); Ramaz Ghlonti (Unimed Ajara Batumi Refferal Hospital, Batumi); Zaza Lominadze (LTD "Clinic-LJ", Kutaisi); George Khabeishvili (Tbilisi Heart and Vascular Clinic, Ltd, Tbilisi).

Germany (509 patients enrolled) Rayyan Hemetsberger and Kemala Edward (St. Johannes Hospital, Dortmund); Ursula Rauch-Kröhnert (Charité - Universitätsmedizin Campus Benjamin Franklin, Berlin); Matthias Stratmann (Kardiologische Gemeinschaftspraxis, Dortmund); Karl-Friedrich Appel (Studienzentrum Dr. Appel, Kassel); Ekkehard Schmidt (Cardiologicum Hamburg, Hamburg); Heyder Omran (GFO Kliniken Bonn, Bonn); Christoph Stellbrink (Klinikum Bielefeld Mitte, Bielefeld); Thomas Dorsel (Joseph-Hospital Warendorf, Warendorf); Emmanouil Lianopoulos and Hans Friedrich Vöhringer (DRK Kliniken Berlin-Köpenick, Berlin); Roger Marx (MediClin Fachklinik Rhein/Ruhr, Essen); Andreas Zirlik (Universitätsherzzentrum Freiburg, Freiburg); Detlev Schellenberg (Institut für Klinische Forschung, Rotenburg an der Fulda); Thomas Heitzer (Klinikum Dortmund, Dortmund); Ulrich Laufs and Christian Werner (Universitätsklinikum des Saarlandes, Homburg/Saar); Nikolaus Marx (Universitätsklinikum Aachen, Aachen); Stephan Gielen and Sebastian Nuding (Universitätsklinikum Halle/Saale, Halle (Saale); Bernhard Winkelmann (ClinPhenomics GmbH & Co. KG, Frankfurt am Main); Steffen Behrens (Vivantes Humboldt-Klinikum, Berlin); Karsten Sydow and Mahir Karakas (Universitäres Herzzentrum Hamburg GmbH, Hamburg); Gregor Simonis (FAZ Dresden-Neustadt GbR, Dresden); Thomas Muenzel (Universitätsklinikum Mainz, Mainz); Nikos Werner (Universitätsklinikum Bonn, Bonn); Stefan Leggewie (Universitäts-Herzzentrum Freiburg-Bad Krozingen, Bad Krozingen); Dirk Böcker (St. Marien-Hospital Hamm, Hamm); Rüdiger Braun-Dullaeus (Universitätsklinikum Magdeburg, Magdeburg); Nicole Toursarkissian (Praxis Dr. Nicole Toursarkissian, Berlin); Michael Jeserich (GP Dr. Haggenmiller, PD Dr. Michael Jeserich, Nürnberg); Matthias Weißbrodt (Gemeinschaftspraxis Dr. M. Löbe, Dr. M. Weißbrodt, Leipzig); Tim Schaeufele (Robert-Bosch-Krankenhaus GmbH, Stuttgart); Joachim Weil (Sana Kliniken Lübeck, Lübeck); Heinz Völler (Klinik am See, Rüdersdorf bei Berlin); Johannes Waltenberger (Universitätsklinikum Münster, Münster); Mohammed Natour (HPK-Heidelberger Privatklinik für

Kardiologie, Heidelberg); Susanne Schmitt and Dirk Müller-Wieland (Asklepios Klinik St. Georg., Allgemeine Innere Medizin, Hamburg); Stephan Steiner (Krankenhausgesellschaft St. Vincenz mbH, Limburg); Lothar Heidenreich and Elmar Offers (Dreifaltigkeits-Hospital Lippstadt, Lippstadt); Uwe Gremmler (MVZ Ambulantes Kardiologisches Zentrum Peine GbR, Peine); Holger Killat (Praxis Dr. Holger Killat, Haßloch); Werner Rieker (Studienzentrum Rankestrasse, Berlin).
Greece (70 patients enrolled) Sotiris Patsilinakos ("Konstantopouleio" General Hospital of Nea Ionia, Athens); Athanasios Kartalis ("Skylitseio" General Hopsital of Chios, Chios); Athanassios Manolis ("Asklipeio" General Hospital, Athens); Dimitrios Sionis and Geargios Chachalis ("Sismanogleio" General Hospital of Attica, Marousi); Evangelos Liberopoulos (University General Hospital of Ioannina, Ioannina); Ioannis Skoumas ("Ippokrateio" General Hospital, Athens); Vasilios Athyros ("Ippokrateio" General Hospital, Thessaloniki); Panagiotis Vardas and Frangkiskos Parthenakis (University General Hospital of Heraklion, Crete); previous PI Dimitrios Alexopoulos / current PI Georgios Hahalis (University General Hospital of Patra, Patra); John Lekakis ("Attikon" University General Hospital, Athens); Apostolos Hatzitolios (AHEPA General Hospital of Thessaloniki, Thessaloniki).

Guatemala (25 patients enrolled) Sergio R Fausto Ovando (Cilinical Research Center, Guatemala); Pablo Carlos Montenegro Valdovinos (Clinica Privada, Guatemala); Juan L Arango Benecke (Clinicas Medicas; Dr. Arango, Guatemala); Edgar R Rodriguez De Leon (Unidad de Diagnostico Cardiologico, Guatemala).

Hong Kong (17 patients enrolled) Bryan PY Yan (Prince of Wales Hospital, Shatin, NT); David CW Siu (Queen Mary Hospital).

Hungary (224 patients enrolled) Tibor Turi (Fejér Megyei Szent György Egyetemi Oktató Kórház, Székesfehérvár); Bela Merkely (Semmelweis Egyetem Városmajori Szív-és Érgyógyászati Klinika, Budapest); Robert Gabor Kiss (Magyar Honvédség Egészségügyi Központ, Budapest); Imre Ungi (Szegedi Tudományegyetem, Szeged); Geza Lupkovics (Zala Megyei Szent Rafael Kórház, Zalaegerszeg); Lajos Nagy (Markusovszky Egyetemi Oktatókórház, Szombathely); András Katona (Békés Megyei Központi Kórház, Gyula); István Édes (Debreceni Egyetem Klinikai Központ, Debrecen); Gábor Müller (Markhot Ferenc Oktatókórház és Rendelointézet, Eger); Iván Horvath (Pécsi Tudományegyetem Klinikai Központ, Pécs); Tibor Kapin and Zsolt Szigeti (Bajcsy-Zsilinszky KórházRendelöintézet, Budapest); József Faluközy (Állami Szívkórház Balatonfüred, Balatonfüred).

India (521 patients enrolled) Mukund Kumbla (Omega Hospital, Mangalore); Manjinder Sandhu (Artemis Health Institute, Gurgaon); Sharath Annam (Sunshine Hospital, Secunderabad); Naveen Reddy Proddutur and Reddy Regella (Mediciti Hospitals, Hyderabad); Rajendra K Premchand (Krishna Institute Of Medical Sciences, Secunderabad); Ajaykumar Mahajan and Sudhir Pawar (Lokmanya Tilak Municipal Medical College & General Hospital, Mumbai); Atul D Abhyanakar (Nirmal Hospital Pvt Ltd, Surat); Prafulla Kerkar (Seth GS Medical College & KEM Hospital, Mumbai); Ravishankar A Govinda (K.R.Hospital, Mysore); Abraham Oomman (Apollo Hospital, Chennai); Dhurjati Sinha (ICVS at IPGME&R, SSKM Hospital, Kolkata); Sachin N Patil (Aster Aadhar Hospital, Kolhapur); Dhiman Kahali (B. M. Birla Heart Research Centre, Kolkata); Jitendra Sawhney (Dharma Vira Heart Center, Sir Ganga Ram Hospital, Delhi); Abhijeet B Joshi (Niramay Hospital, Pune); Sanjeev Chaudhary (Fortis Hospital, Gurgaon); Pankaj Harkut (Meditrina Institute of Medical Sciences, Nagpur); Santanu Guha (Medical College and Hospital, Kolkata); Sanjay Porwal (KLEs Dr Prabhakar Kore Hospital & Medical Research Centre, Belgaum); Srimannarayana Jujjuru (Andhra Hospital, Vijaywada); Ramesh B Pothineni (Dr. Ramesh Cardiac & Multispeciality Hospital Ltd., Vijayawada); Minguel R Monteiro (UGH Vintage Hospital & Medical Research Centre Pvt Ltd, Panaji); Aziz Khan (Crescent hospital and heart clinic, Nagpur); Shamanna S Iyengar (Manipal Hospital, Bangalore, Karnataka); Jasprakash Singh Grewal (Satguru Pratap Singh Apollo Hospitals, Ludhiana); Manoj Chopda (Magnum Heart Institute, Nashik); Mahesh C Fulwani (Shrikrishna Hrudayalay and Critical Care Centre, Nagpur); Dr. Aparna Patange and Patil Sachin (Krishna Institute of Medical Sciences Deemed University, Karad, Maharashtra); Vijay K Chopra (Medanta - The Medicity, New Delhi); Naresh K Goyal (Max Super Speciality Hospital, Delhi, Delhi); Rituparna Shinde (Sanjeevan Hospital, Pune); Gajendra V Manakshe (Datta Meghe Institute of Medical Sciences (Deemed University, Wardha); Nitin Patki (Maharashtra Medical Foundation Joshi Hospital, Pune, Maharashtra); Sumeet Sethi (Max Heart & Vascular Institute, New Delhi); Vengatesh Munusamy (SRM Medical College Hospital And Research Centre, Chennai); Sunil Karnaand Sunil Thanvi (Bahnubhai And Madhuben Patel Cardiac Centre, Karamsad); Srilakshmi Adhyapak and Chandrakant

Patil (St John's Medical College & Hospital, Bangalore); Ulhas Pandurangi (Madras Medical Mission, Chennai); Rishabh Mathur and Jugal Gupta (S. R. Kalla Memorial Gastro & General Hospital, Jaipur); Suhas Kalashetti (K. E .M Hospital, Pune, Maharashtra); Ajit Bhagwat (Kamal Nayan Bajaj Hospital, Aurangabad); Bagirath Raghuraman (Chinmaya Narayana superspecility hospital, Bangalore); Shiv Kumar Yerra (Mahavir Hospital And Research Centre, Hyderabad); Prasant Bhansali (AMC-MET medical College and Sheth L G General Hospital, Ahmedabad); Rohidas Borse and Patil Rahul (B.J. Government Med. College and Sassoon General Hospitals, Pune); Srihari Das and Vinay Kumar (NH-Narayana Multispecialty hospital, Bangalore); Jabir Abdullakutty (Lisie Hospital, Cochin); Shireesh Saathe (Deenanath Mangeshkar Hospital, Pune); Priya Palimkar (Jehangir Clinical Development centre, Pune); Jabir Abdullkutty (Lisie Hospital, Kochi); Shireesh Sathe (Dmangeshkar Hospital, Pune); Priya Palimkar (Jehangir Hospital, Pune).

Israel (582 patients enrolled) Shaul Atar (Western Galilee Medical Center, Nahariya); Michael Shechter (Sheba MC, Tel HaShomer); Morris Mosseri (Meir Medical Center, Kfar Saba); Yaron Arbel, Chorin Ehud and Havakuk Ofer (Tel Aviv Sourasky MC, Tel Aviv); Chaim Lotan (Hadassah Ein Kerem MC, Jerusalem); Uri Rosenschein (Bnai Zion Medical Center, Haifa); Amos Katz (Barzilai Medical Center, Ashkelon); Yaakov Henkin (Soroka Medical Center, Beer Sheva); Adi Francis (Holy Family Hospital, Nazareth); Marc Klutstein (Shaare Zedek Medical Center, Jerusalem); Eugenia Nikolsky and Robert Zukermann (Rambam MC, Haifa); Yoav Turgeman (Haemek Medical Center, Afula); Majdi Halabi and Alon Marmor (Ziv Medical Center, Safed); Ran Kornowski (Rabin Mc Belinson Campus, Petah-Tikva); Michael Jonas (Kaplan Medical Center, Rehovot); Offer Amir and Yonathan Hasin (The Baruch Padeh Medical Center, Poriya, Tiberias); Yoseph Rozenman (Wolfson Mc, Holon); Shmuel Fuchs and Vered Zvi (Rabin Mc Belinson Campus, Petah-Tikva); Osamah Hussein (Ziv Medical Center, Safed); Dov Gavish (Wolfson Mc, Holon); Zvi Vered (Assaf Harofe Medical Center, Tzrifin); Yoseph Caraco (Hadassah Ein Kerem MC, Jerusalem); Mazen Elias (Haemek Medical Center, Afula); Naveh Tov and Efrat Wolfovitz (Bnai Zion Medical Center, Haifa); Michael Lishner (Meir Medical Center, Kfar Saba); Nizar Elias (Bnei Zion MC, Haifa).

Italy (275 patients enrolled) Giancarlo Piovaccari (Ospedale Degli Infermi Rimini, Rimini); Annamaria De Pellegrin and Raffaella Garbelotto (UO Medicina Generale Ospedale di Vittorio Veneto, Vittorio Veneto); Gabriele Guardigli and Valgimigli Marco (Azienda Ospedaliera S. Anna, Ferrara); Giovanni Licciardello (Ospedale E. Moscatello, Augusta, Salerno); Carla Auguadro and Filippo Scalise (Policlinico di Monza, Monza); Claudio Cuccia (Fondazione Poliambulanza - Istituto Ospedaliero, Brescia); Alessandro Salvioni (Centro Cardiologico Fondazione Monzino, Milano); Giuseppe Musumeci and Michelle Senni (ASST-Papa Giovanni XXII, Bergamo); Paolo Calabrò (Azienda Ospedaliera Monaldi, Napoli); Salvatore Novo (A O Universitaria Policlinico P Giaccone, Palermo); Pompilio Faggiano and Marco Metra (Azienda Ospedaliera Spedali Civili di Brescia, Brescia); Nicoletta B De Cesare (Policlinico San Marco - Istituti Ospedalieri Bergamaschi, Osio Sotto, Bergamo); Sergio Berti (Fond.Toscana G.Monasterio-Presidio di Massa-Osped. del Cuore, Massa, Massa-Carrara); Claudio Cavallini (Ospedale S Maria Della Misericordia, Perugia); Enrico Puccioni (Ospedale Civile di Livorno - Divisione Cardiologia-UTIC, Livorno); Marcello Galvani (Ospedale Morgagni-Pierantoni, Forlì, Forlì-Cesena); Maurizio Tespili (Ospedale Bolognini, Seriate, Bergamo); Piermarco Piatti (Ospedale San Raffaele, Milano); Michela Palvarini (A.O. Guido Salvini - P.O. di Passirana, Rho, Milano); Giuseppe De Luca (Azienda Ospedaliera Maggiore Della Carità, Novara); Roberto Violini (Azienda Ospedaliera San Camillo-Forlanini, Roma); Alessandro De Leo and Zoran Olivari (Ospedale Civile Treviso Presidio Ospedaliero Ca' Foncello, Treviso); Pasquale Perrone Filardi (Az. Ospedaliera Univ. Federico II, Napoli); Maurizio Ferratini (IRCCS Fondazione "Don Gnocchi" Centro "S. Maria Nacente", Milano); Vittorio Racca (IRCCS Fondazione "Don Gnocchi" Centro "S, Maria Nacente" – Cardiologia, Milano).

Japan (204 patients enrolled) Kazuoki Dai and Yuji Shimatani (Hiroshima City Hospital, Hiroshima-shi, Hiroshima); Haruo Kamiya (Japanese Red Cross Nagoya Daiichi Hospital, Nagoya-shi, Aichi); Kenji Ando (Kokura Memorial Hospital, Kitakyushu-shi, Fukuoka); Yoshihiro Takeda (Rinku General Medical Center, Izumisano-shi, Osaka); Yoshihiro Morino (Iwate Medical University Hospital, Morioka-shi, Iwate); Yoshiki Hata (Minamino Cardiovascular Hospital, Hachioji-shi, Tokyo); Kazuo Kimura (Yokohama City University Medical Center, Yokohama-shi, Kanagawa); Koichi Kishi (Tokushima Red Cross Hospital, Komatsushima-shi, Tokushima); Ichiro Michishita (Yokohama Sakae Kyosai Hospital, Yokohama-shi, Kanagawa); Hiroki Uehara (Urasoe General Hospital, Urasoe-shi, Okinawa); Toshinori Higashikata

(National Health Insurance Komatsu Municipal Hospital, Komatsu-shi, Ishikawa); Atsushi Hirayama (Nihon University Itabashi Hospital, Itabashi-ku, Tokyo); Keiji Hirooka and Yasuji Doi (Saiseikai Senri Hospital, Suita-shi, Osaka); Satoru Sakagami (Kanazawa Medical Center, Kanazawa-shi, Ishikawa); Shuichi Taguchi (Mito Medical Center, Higashiibaraki-gun, Ibaraki); Akihiro Koike (Fukuoka-Higashi Medical Center, Koga-shi, Fukuoka); Hiroyuki Fujinaga (Tokushima Prefectural Central Hospital, Tokushima-shi, Tokushima); Shinji Koba (Showa University Hospital, Shinagawa-ku, Tokyo); Ken Kozuma (Teikyo University Hospital, Itabashi-ku, Tokyo); Tomohiro Kawasaki (Shin-Koga Hospital, Kurume-shi, Fukuoka); Yujiro Ono (Higashi-Hiroshima Medical Center, Higashihiroshima-shi, Hiroshima); Masatoshi Shimizu (Kobe Medical Center, Kobe-shi, Hyogo); Yousuke Katsuda (Fukuoka City Medical Association Hospital, Fukuoka-shi, Fukuoka); Atsuyuki Wada (Kusatsu General Hospital, Kusatsu-shi, Shiga); Toshiro Shinke (Kobe University Hospital, Kobe-shi, Hyogo); Takeshi Kimura (Kyoto University Hospital, Kyoto-shi, Kyoto); Junya Ako (Kitasato University Hospital, Sagamihara-shi, Kanagawa); Kenshi Fujii (Sakurabashi Watanabe Hospital, Osaka-shi, Osaka); Toshiyuki Takahashi (Tokyo Saiseikai Central Hospital, Minato-ku, Tokyo); Tomohiro Sakamoto and Koichi Nakao (Saiseikai Kumamoto Hospital, Kumamoto-shi, Kumamoto); Yutaka Furukawa (Kobe City Medical Center General Hospital, Kobe-shi, Hyogo); Hiroshi Sugino and Ritsu Tamura (Kure Medical Center and Chugoku Cancer Center, Kure-shi, Hiroshima); Toshiaki Mano and Masaaki Uematsu (Kansai Rosai Hospital, Amagasaki-shi, Hyogo); Noriaki Utsu (Minami Osaka Hospital, Osaka-shi, Osaka); Kashima Ito (Nerima Hospital, Nerima-ku, Tokyo); Takuya Haraguchi and Katsuhiko Sato (Caress Sapporo Tokeidai Memorial Clinic, Sapporo-shi, Hokkaido); Yasunori Ueda (Osaka National Hospital, Osaka-shi, Osaka); Akira Nishibe (Amagasaki New Town Hospital, Amagasaki-shi, Hyogo); Kazuteru Fujimoto (Kumamoto Medical Center, Kumamoto-shi, Kumamoto); Motomaru Masutani and Akira Nishibe (Human Medical Amagasaki New Town Hospital, Amagasaki-shi); Kazuteru Fujimoto (National Hospital Organization Kumamoto Medical Center, Kumamoto-shi).

Korea, Republic of (94 patients enrolled) Jung Han Yoon (Yonsei University Wonju Severance Christian Hospital, Wonju, Gangwon-do); **Sang-Hyun Kim and Hack-Lyoung Kim** (SMG Seoul National University Boramae Medical Center, Seoul, Seoul); Hun Sik Park (Kyungpook National University Hospital, Daegu); In-Ho Chae (Seoul National University Bundang Hospital, Seongnam, Gyeonggi-do); Moo Hyun Kim (Dong-A University Medical Center, Busan); Myung Ho Jeong (Chonnam National University Hospital, Gwangju, Gwangju); Seungwoon Rha (Korea University Guro Hospital, Seoul); Chongjin Kim (KyungHee University Hospital at Gangdong, Seoul, Seoul); Hyo-Soo Kim and Hae Young Kim (Seoul National University Hospital, Seoul, Seoul); Taekjong Hong (Pusan University Hospital, Busan, Busan); Seung-Jea Tahk (Ajou University Hospital, Suwon, Gyeonggi-Do); Youngkwon Kim (Dongguk University Medical Center, Goyang-si, Gyeonggi-Do).

Latvia (80 patients enrolled) Arija Busmane (Adoria, Sabiedriba ar ierobežotu atbildibu, Riga); Natalija Pontaga (SIA "Daugavpils regionala slimnica, Daugavpils); Aldis Strelnieks (Rigas Austrumu kliniska universitates slimnica, SIA, Riga); Iveta Mintale (Pauls Stradins Clinical University Hospital, Riga); Iveta Sime (Regional Hospital of Liepaja, Liepaja).

Lithuania (188 patients enrolled) Zaneta Petrulioniene (Vilnius University Hospital Santaros klinikos, Vilnius); Roma Kavaliauskiene (Clinic of Cardiology and Rehabilitation, Klaipeda); Ruta Jurgaitiene (Hospital of Lithuanian University of Health Sciences, Kaunas); Gintare Sakalyte and Rimvydas Slapikas (Hospital of Lithuanian University of Health Sciences, Kaunas); Sigute Norkiene (Klaipeda Seamen's hospital, Klaipeda); Nerijus Misonis (Private Medicine Center Kardivita, Vilnius); Aleksandras Kibarskis (Private Cardiologic Clinic Sirdies Namai, Vilnius); Raimondas Kubilius (Elite Medicale, Kaunas).

Macedonia, The Former Yugoslav Republic of (132 patients enrolled) Stojko Bojovski (Cardiological Diagnostic Center, Tetovo); Sasko Kedev (University Clinic of Cardiology, Skopje); Nensi Lozance (Clinical hospital dr. Trifun Panovski, Bitola, Bitola); Aleksandar Kjovkaroski (Military medical Center, Skopje); Snezana Doncovska (PHI Clinical hospital Shtip, Shtip).

Malaysia (110 patients enrolled) Tiong Kiam Ong (Sarawak General Hospital, Kuching); Sazzli Kasim (Universiti Teknologi MARA (UiTM), Sungai Buloh Campus, Sungai Buloh, Selangor); Oteh Maskon (Hospital Universiti Kebangsaan Malaysia, Kuala Lumpur); Balachandran Kandasamy (Institut Jantung Negara, Kuala Lumpur, Kuala Lumpur); Khalid Yusoff (Universiti Teknologi MARA (UiTM) Selayang

Campus, Batu Caves and UCSI University); Houng B Liew (Queen Elizabeth Hospital II, Kota Kinabalu); Wan Mohd Izani Wan Mohamed (Hospital Universiti Sains Malaysia, Kota Bharu).

Mexico (349 patients enrolled) Armando García Castillo (Cardiolink Clin Trials S.C., Monterrey, Nuevo León); Gabriel Arturo Ramos López (Medical Office, Guadalajara, Jalisco); Jorge Carrillo Calvillo (Hospital Central "Dr. Ignacio Morones Prieto", San Luis Potosi, San Luis Potosi); Pedro Fajardo Campos (CINCAME Centro de Investigación Cardiovascular y Metabólica, Tijuana, Baja California Norte); Juan Carlos Núñez Fragoso (Lahoja Asociación para la Investigación y la Farmacovigilancia S.C., Durango, Durango); Edmundo Alfredo Bayram Llamas (Fundación Cardiovascular de Aguascalientes A.C., Aguascalientes, Aguascalientes); Marco Antonio Alcocer Gamba (Centro de Estudios Clínicos de Querétaro, S.C., Querétaro, Querétaro); Jaime Carranza Madrigal (Unidad de Prevención y Atención Metabólica, Morelia, Michoacan); Luis Gerardo González Salas (Centro para el Desarrollo de la Medicina y de Asistencia Médica Especializada, S.C., Culiacán, Sinaloa); Enrique López Rosas (Centro de Alta Especialidad "Dr. Rafael Lucio", Jalapa, Veracruz); Belinda González Díaz (UMAE Hospital de Cardiología del Centro Médico Nacional Siglo XXI., Ciudad de México, México); Eduardo Salcido Vázquez (Antiguo Hospital Civil de Guadalajara "Fray Antonio Alcalde", Guadalajara, Jalisco); Alfredo Nacoud Ackar (Hospital Universitario "Dr. José Eleuterio González", Monterrey, Nuevo León); Guillermo Antonio Llamas Esperón (Hospital Cardiológica Aguascalientes, Aguascalientes, Aguascalientes); Carlos Rodolfo Martínez Sánchez (Instituto Nacional de Cardiología "Ignacio Chávez", Ciudad de México, México); María Guerrero De Leon (Avix Investigación Clínica, S.C., Monterrey, Nuevo León); Rodrigo Suarez Otero (INBIOMEDYC Toluca, Toluca, México); Guillermo Fanghänel Salmón (Clínica Integral del Paciente Diabético y Obeso, Ciudad de México, México); Jesús Antonio Pérez Ríos (OSMO Oaxaca Site Management Organization S.C., Oaxaca, Oaxaca); José Angel Garza Ruíz (IMED Internal Medicine Clin Trials, Monterrey, Nuevo León).

Netherlands (686 patients enrolled) Marco Alings (Amphia Ziekenhuis Molengracht, Breda); Robert W Breedveld, Margriet Feenema-Aardema and Alida Borger-Van Der Burg (Medisch Centrum Leeuwarden, Leeuwarden); Pieter AM Hoogslag (Diaconessenhuis Meppel, Meppel); Harry Suryapranata (Radboud University Medical Center, Nijmegen); Antonius Oomen, Paulus Van Haelst and Margriet Feenema-Aradema (D & A Research, Sneek); Jacobijne J Wiersma and Dirk Basart (Vasculair Onderzoeks Centrum BV, Hoorn); Ruud MA Van Der Wal and Peter Zwart (Ziekenhuis Bernhoven, Uden); Pascalle Monraats and Henricus Van Kesteren (Admiraal De Ruiter Ziekenhuis, Goes); Ioannis Karalis and Johan Jukema (Leiden University Medical Center, Leiden); Gerardus JE Verdel (Spaarne Gasthuis, Haarlem); Bart RG Brueren (Catharina Ziekenhuis, Eindhoven); Roland PTh Troquay (VieCuri Medisch Centrum Voor Noord-Limburg, Venlo); Eric P Viergever (Groene Hart Ziekenhuis, Gouda); Nadea YY Al-Windy (Gelre Ziekenhuis Zutphen, Zutphen); Gerard L Bartels (Martini Ziekenhuis, Groningen); Jan H Cornel (Noordwest Ziekenhuisgroep, locatie Alkmaar, Alkmaar); Walter RM Hermans (St Elisabeth Ziekenhuis, Tilburg); Johannes PR Herrman (OLVG locatie Oost, Amsterdam); Robert J Bos (Bravis Ziekenhuis, Roosendaal); Reginald GEJ Groutars (OLVG locatie Oost, Amsterdam); Coenraad C Van Der Zwaan (Ziekenhuis Rivierenland, Tiel); Refik Kaplan and Raymond Lionarons (Röpcke-Zweers Ziekenhuis, Hardenberg); Eelko Ronner (Reinier De Graaf Gasthuis, Delft); Bjorn E Groenemeijer (Gelre Diagnostisch Centrum, Apeldoorn); Patrick NA Bronzwaer (Zaans Medisch Centrum, Zaandam); Anho AH Liem (Sint Franciscus Gasthuis, Rotterdam); Bernard JWM Rensing (St Antonius Ziekenhuis, Nieuwegein); Marcel JJA Bokern (Waterlandziekenhuis, Purmerend); Remco Nijmeijer (Tjongerschans Hospital Heerenveen, Heerenveen); Ferry MRJ Hersbach (Maasstad ziekenhuis, Rotterdam); Frank F Willems (Rijnstate Ziekenhuis, Arnhem); Antonius TM Gosselink and Saman Rasoul (Isala Klinieken, Zwolle).

New Zealand (257 patients enrolled) John Elliott (Christchurch Hospital, Christchurch, Canterbury); Gerard Wilkins (Dunedin Hospital, Dunedin, Otago); Raewyn Fisher (Waikato Hospital, Hamilton, Waikato); Douglas Scott (Middlemore Hospital, Otahuhu, Auckland); Hamish Hart (North Shore Hospital, Takapuna, Auckland); Ralph Stewart (Auckland City Hospital, Grafton, Auckland); Scott Harding (Wellington Hospital, Newtown, Wellington); Ian Ternouth (Taranaki Base Hospital, New Plymouth, Taranaki); Nicholas Fisher and Samuel Wilson (Nelson Hospital, Nelson, Nelson); Denise Aitken (Lakes District Health Board, Rotorua, Bay of Plenty); Russell Anscombe (Hutt Valley District Health Board, Lower Hutt, Wellington); Laura Davidson (Palmerston North Hospital, Palmerston North, Manawatu-Wanganui).

Norway (97 patients enrolled) Tadeusz Tomala (Svelvik Legesenter, Svelvik); Ottar Nygård (Helse Bergen HF, Bergen); Jon Arne Sparby (Sykehuset innlandet HF, Kongsvinger, Kongsvinger); Kjell Andersen (Sykehuset Innlandet HF, Hamar, Hamar); Lars Gullestad (Oslo Universitetssykehus HF, Oslo); Jarle Jortveit (Sørlandet sykehus HF Arendal, Arendal); Peter S Munk and Erlend gyllensten Singsaas (Stavanger Helseforskning AS, Stavanger); Sigrun Halvorsen (Oslo Universitetssykehus HF, Oslo); Ulf Hurtig (Sykehuset Innlandet HF, Tynset).

Peru (208 patients enrolled) Roger M Correa Flores (Hospital Nacional IV Alberto Sabogal Sologuren, Callao); Jorge R Calderon Ticona (Novocardio Centro de Investigacion y Atencion Cardiovascular, Lima); Julio R Durand Velasquez (Centro de Invesgaciones Médicas - Hospital Maria Auxiliadora, Lima); Sandra A Negron Miguel (Clínica Internacional Sede San Borja, Lima); Enrique S Sanabria Perez (Clinica Javier Prado, Lima); Jesus M Carrion Chambilla (Hospital Nacional Arzobispo Loayza, Lima); Carlos A Chavez Ayala (Hospital Nacional Daniel Alcides Carrión, Callao); Reynaldo P Castillo Leon (Clínica San Gabriel - Consultorio 316, Lima); Rolando J Vargas Gonzales (Clínica Virgen María Auxiliadora, Piura); Jose D Hernandez Zuniga (Clinica Medica San Martin E.I.R.L, Ica); Luis A Camacho Cosavalente (Clínica Peruano Americana, Trujillo); Jorge E Bravo Mannucci (Clínica Divino Niño Jesus Orden de Malta,); Javier Heredia Landeo (Clinica Angloamericana, Clinica Angloamericana, Lima); Nassip C Llerena Navarro (Hospital Nacional Carlos Alberto Seguín Escobedo, Arequipa); Yudy M Roldan Concha (Hospital Nacional Hipolito Unanue, Lima); Víctor E Rodriguez Chavez (Hospital Central de la Fuerza Aerea Peruana, Lima); Henry A Anchante Hernandez (Hospital Nacional Cayetano Heredia, Lima); Carlos A Zea Nunez (Hospital Nivel IV Adolfo Guevara Velasco, Cuzco); Walter Mogrovejo Ramos (Instituto Neuro Cardiovascular de las Americas, Lima).

Philippines (116 patients enrolled) Arthur Ferrolino (Philippine Heart Center, Quezon City); Rosa Allyn G Sy (Ospital ng Makati Diabetes Clinic, Makati City); Louie Tirador (St. Paul's Hospital, Iloilo City); Rody G. Sy (Cardinal Santos Medical Center, San Juan); Generoso Matiga (Perpetual Succour Hospital/Cebu Heart Institute, Cebu City); Raul Martin Coching (Davao Doctors Hospital, Davao); Alisa Bernan (Community Health and Development Cooperative Hospital., Davao City); Gregorio Rogelio (St. Luke's Medical Center, Quezon); Dante D. Morales (Manila Doctors Hospital, Manila); Edgar Tan (Cebu Doctors University Hospital, Cebu City); Dennis Jose Sulit (Quirino Memorial Medical Center, Quezon City).

Poland (926 patients enrolled) Adrian Wlodarczak (Miedziowe Centrum Zdrowia, Lubin,); Krystyna Jaworska, Grzegorz Skonieczny (Wojewodzki Szpital Zespolony im. L. Rydygiera, Torun,); Lidia Pawlowicz (Prywatne Centrum Kardiologii, Torun); Pawel Wojewoda (Poradnia Kardiologiczna Centrum Medyczne Ogrodowa, Skierniewice,); Benita Busz-Papiez (Indywidualna Specjalistyczna Praktyka Lekarska, Szczecin); Janusz Bednarski (Samodzielny Pub. Spec. Szp. Zachodni im. Jana Pawla II, Grodzisk Mazowiecki); Aleksander Goch (10 Wojskowy Szpital Kliniczny z Poliklinika, Bydgoszcz); Pawel Staneta (NZOZ Specjalistyczna Przychodnia Lekarska „Medikard”, Plock); Elzbieta Dulak (NZOZ "Srodmiescie" Sp. z o.o., Gdynia); Andrzej Budaj (Centrum Medyczne Ksztalcenia Podyplomowego, Szpital Grochowski im. dr med. Rafala Masztaka, Warszawa); Krzysztof Saminski (Centrum Kardiologiczne "Pro Corde" Sp. z o.o., Wroclaw); Wlodzimierz Krasowski (COPERNICUS Podmiot Leczniczy Sp. z o.o. Szpital Sw. Wojciecha Gdansk); Wanda Sudnik (Centrum Medyczne Dr Sudnik s.c. W&J Sudnik, Sokolka); Aleksander Zurakowski (Malopolskie Centrum Sercowo-Naczyniowe, Chrzanow); Marcin Skorski (SPZOZ w Lecznej, Leczna); Roman Lysek (Przychodnia Lecznice CITOMED Sp. z o.o., Torun); Beata Miklaszewicz (B.Miklaszewicz & D.Dabrowski "CARDIAMED" Spolka Jawna, Legnica); Jacek Kubica (Szpital Uniwersytecki im. dr A. Jurasza, Bydgoszcz); Jan Andrzej Lipko (5 Wojskowy Szpital Kliniczny z Poliklinika SPZOZ, Krakow); Edyta Kostarska-Srokosz (Centrum Medyczne „Nasze Zdrowie”, Warszawa); Marek Piepiorka (Gabinet Kardiologiczno-Internistyczny, Gdynia); Anna Drzewiecka (NZOZ "Centrum-Med" s.c., Czeladz); Ryszard Sciborski (SPZOZ, Olawa); Arkadiusz Stasiewski (NZOZ Neuro-Kard Ilkowski i Partnerzy, Poznan); Tomasz Blicharski (Lubelskie Centrum Diagnostyczne, Swidnik); Leszek Bystryk (Wojewodzkie Centrum Szpitalne Kotliny Jeleniogorskiej, Jelenia Gora); Michal Szpajer (Poliklinika evi-, Gdynia); Marek Korol (Osrodek Medycyny Rodzinnej Sp. z o.o., Sobotka); Tomasz Czerski (SPZOZ w Wegrowie, Wegrów); Ewa Mirek-Bryniarska (Szpital Specjalistyczny im. J. Dietla, Krakow); Jacek Gniot (SPZOZ w Pulawach, Pulawy); Andrzej Lubinski (Indywidualna Specjalistyczna Praktyka Lekarska A. Lubinski, Lodz); Jerzy Gorny (Wojewodzki Szpital Specjalistyczny, Olsztyn); Edward Franek (Clinical Research Group Sp. z o.o., Warszawa); Grzegorz

Raczak (Centrum Kardiologii Uniwersyteckie Centrum Kliniczne, Gdansk); Hanna Szwed (Instytut Kardiologii, Klinika Choroby Wiencowej, Warsaw).

Portugal (174 patients enrolled) Pedro Monteiro (Centro Hosp e Univ de Coimbra - Hosp da Univ de Coimbra, Coimbra); Jose Mesquita Bastos (Centro Hospitalar de Baixo Vouga, Aveiro); Helder H Pereira (Hospital Garcia De Orta, Almada); Dinis Martins (Hospital Do Divino Espírito Santo, Ponta Delgada); Joao Morais (Centro Hospitalar Leiria Pombal, Leiria); Filipe Seixo (Centro Hospitalar De Setubal EPE, Setubal); Carlos Mendonça (Hospital de Vila Franca de Xira, Vila Franca de Xira); Ana Botelho and Francisca Caetano (Centro Hosp e Univ de Coimbra - Hosp da Univ de Coimbra, Coimbra).

Romania (145 patients enrolled) Bogdan Minescu (Spitalul Judetean De Urgenta Braila, Braila); Octavian Istratoaie (Spitalul Clinic Judetean De Urgenta, Craiova); Dan N Tesloianu (S.C. CARDIOMED SRL, Iasi); Maria Dorobantu (Spitalul Clinic De Urgenta Bucuresti, Bucuresti); Gabriel Cristian and Silviu Dumitrescu (SCUMC "dr. Carol Davila", Bucharest); Cristian GC Podoleanu (Podoleanu G. Cristian Gheorghe Calin, Targu Mures); Mircea CA Constantinescu (S.C. Medcon S.R.L., Buzau); Cristina M Bengus (Spitalul Judetean de Urgenta "Sf. Pantelimon" Focsani, Focsani); Constantin Militaru (Cardiomed SRL, Craiova); Doina Rosu (Spitalul Clinic Judetean de Urgenta "Pius Branzeu", Timisoara); Irinel R Parepa (Spitalul Clinic Judetean de Urgenta Constanta, Constanta); Adrian V Matei (Institutul de Urgenta pentru Boli Cardiovasculare si Transpl, Targu Mures); Tom M Alexandru and Mihaela Malis (Impatients SRL, Codlea); Ioan Coman (Alcor Med Srl, Bucuresti); Rodica Stanescu Cioranu (Clinica Angiomed SRL, Bucuresti); Doina Dimulescu (Spitalul Clinic De Urgenta "elias", Bucuresti).

Russian Federation (1109 patients enrolled) Yury Shvarts (Faculty Therapy Department of Clinical Hospital n.a. Mirotvo, Saratov); Olga Orlikova (Regional Clinical Cardiology Dispensary, Saratov); Zhanna Kobalava (City Clinical Hospital #64, Moscow); Olga L Barbarash (Scientific & Research Institute of Complex Problems of Cardi, Kemerovo); Valentin Markov (Research Institute Of Cardiology, Tomsk); Nadezhda Lyamina (Saratov Scientific Research Institute Of Cardiology, Saratov); Alexander Gordienko (Medical Military Academy n.a.S.M.Kirov, Saint - Petersburg); Konstantin Zrazhevsky (City Hospital #38 named after Semashko N.A, Saint Petersburg); Alexander Y Vishnevsky (Pokrovskaya City Hospital, St-Petersburg); Victor Gurevich (Central Medical Unit #122, St-Petersburg); Raisa Stryuk (City Clinical Hospital No. 71, Moscow); Nikita V Lomakin (Central Clinical Hospital with Polyclinic, Moscow); Igor Bokarev and Tatiana Khlevchuk (City Clinical Hospital n.a. Eramishantsev, Moscow); Sergey Shalaev (State Autonomous Institution of Healthcare of Tyumen region, Tyumen); Larisa Khaisheva (City Emergency Hospital #2, Rostov-On-Don); Petr Chizhov (Therapeutic Department of Clinical Hospital n.a. N.A. Semash, Yaroslavl); Inna Viktorova (Omsk City Clinical Hospital 1, Omsk); Natalya Osokina and Vladimir Shchekotov (State Budgetary Healthcare Institution Clinical Medical Sani, Perm); Evgenia Akatova (City Clinical Hospital#40of the Department of Healthcare of, Moscow); Galina Chumakova (Altay Regional Cardiology Dispensary, Barnaul); Igor Libov (City Clinical Hospital n.a.Botkin ofMoscow Healthcare depart, Moscow); Mikhail I Voevoda (Institute of Therapy and preventive medicine, Novosibirsk); Tatyana V Tretyakova (City Clinical Hospital #25, Novosibirsk); Evgeny Baranov (City Clin. Hosp. #5 of Nizhni Novgorod district, Nizhny Novgorod); Sergey Shustov (Medical Military Academy n.a.S.M.Kirov, Saint - Petersburg); Sergey Yakushin (Ryazan Regional Clinical Cardiology Dispensary, Ryazan); Ivan Gordeev (City Clinical Hospital #15 n.a. Filatov, Moscow); Niiaz Khasanov (State Med. Uni. based on MSHI City Clinical Hospital #7, Kazan); Olga Reshetko (Regional Clinical Hospital, Saratov); Tatiana Sotnikova (City Clinical Hospital n.a.Botkin ofMoscow Healthcare depart, Moscow); Olga Molchanova (National Research Center For Preventive Medicine, Moscow); Konstantin Nikolaev (City Clinical Hospital #19, Novosibirsk); Liudmila Gapon (Tyumen Cardiology Center, Tyumen); Elena Baranova (St. Petersburg State Med. Univ. named for I. P. Pavlov, Saint-Petersburg); Zaur Shogenov (City Clinical Hospital n.a. Veresaev, Moscow); Elena Kosmachova (Regional Clinical Hospital #1, Krasnodar); Yuriy Karpov (Voronezh City Clin. Hosp. of Emergency Medical Care #1, Voronezh); Yuri Karpov (Russian Cardiological Scientific-Productive Complex, Moscow); Anton Povzun (Saint-Petersburg I.I. Dzhanelidze Research Institute of Emer, Saint-Petersburg); Liudmila Egorova (Saint Luke's Clinical Hospital, Saint Petersburg); Vadim V Tyrenko (Military Medical Academy n.a. Kirov, St-Petersburg); Igor G Ivanov and Masterov Ilya (St. George the Martyr City Hospital, Saint-Petersburg); Sergey Kanorsky (Krasnodar City Clinical Urgent Care Hospital, Krasnodar).

Serbia (255 patients enrolled) Dragan Simic (Clinical Centre of Serbia, Belgrade); Nikola Ivanovic (General Hospital Sremska Mitrovica, Sremska Mitrovica); Goran Davidovic (Clinical Centre Kragujevac,

Kragujevac); Nebojsa Tasic (Institute for Cardiovascular Diseases Dedinje, Belgrade); Milika R. Asanin (Clinical Centre of Serbia, Belgrade); Stevo Stojic (General Hospital Pancevo, Pancevo); Svetlana R. Apostolovic (Clinical Center Nis, Nis); Stevan Ilic (Institute for treatment and rehabilitation “Niska Banja”, Nis); Biljana Putnikovic Tosic (Clinical Hospital Centre Zemun, Belgrade); Aleksandar Stankovic (General Hospital Leskovac, Leskovac); Aleksandra Arandjelovic (Clinical Hospital Centre Zvezdara, Belgrade); Slavica Radovanovic and Branislava Todic (Clinical-Hospital Center Bezanijska Kosa, Belgrade); Arsen D. Ristic (Clinical Centre of Serbia, Belgrade); Jovan Balinovac (General Hospital Valjevo, Valjevo); Dragan V. Dincic (Military Medical Academy, Belgrade); Petar Seferovic and Ana Karadzic (Clinical Center of Serbia, Belgrade); Slobodan Dodic (Institute of Cardiovascular Diseases of Vojvodina, Sremska Kamenica); Sinisa Dimkovic and Tamara Jakimov (Clinical Hospital Center Bezanijska Kosa, Belgrade).

Singapore (49 patients enrolled) Terrance Chua (National Heart Centre, Singapore); Kian-Keong Poh (National University Hospital, Singapore); Hean Yee Ong and Justin Tang I-Shing (Khoo Teck Puat Hospital, Singapore).

Slovakia (340 patients enrolled) Karol Micko (Interna a kardiologicka klinika, Kardiomed s.r.o., Lucenec); Jan Nociar (Interna a kardiologicka ambulancia KARDIO1, s.r.o., Lucenec); Daniel Pella (Cardio D&R s.r.o., Kosice); Peter Fulop (Kardiologicka a interna ambulancia Interna SK, s.r.o., Svidnik); Marian Hranai (Kardiologicka ambulancia, Nitra); Juraj Palka (Kardiologicka a interna ambulancia CORP, s.r.o., Kosice); Juraj Mazur (Kardiologicka a interna ambulancia Kardio-onkologia, s.r.o., Dolny Kubin); Ivan Majercák (Sukromna interna a kardiologicka ambulancia, Kosice); Andrej Dzupina (Alian, s.r.o., Bardejov); František Fazekas (Kardiologicka ambulancia Medicentrum Heart, s.r.o., Kralovsky Chlmec); Jozef Gonsorcik (Nestatna interna a kardiologicka ambulancia, Kosice); Viliam Bugan (Medivasa s.r.o., Zilina); Jan Murin (I. Interna klinika, Univerzitna nemocnica Bratislava, Bratislava); Juraj Selecky (Sciens, s.r.o., Trebisov); Gabriel Kamensky (V. Interna klinika Univerzitna nemocnica Bratislava Ruzinov, Bratislava); Jaroslava Strbova (Kardiologicka, interna a sonograficka amb. Kardio-Sanus, sro, Bratislava); Rudolf Smik (Interna, kardiologicka, angiologicka ambul. Medikard, s.r.o., Presov); Andrej Dukat (Univerzitna nemocnica Bratislava, Bratislava); Peter Olexa (Topcare s.r.o, Kosice).

Slovenia (36 patients enrolled) Ivan Žuran (General Hospital Celje, Celje); Janez Poklukar (General Hospital Jesenice, Jesenice); Nataša Černič Šuligoj (General Hospital Izola, Izola); Matija Cevc (Department of Vascular Medicine, Division of Medicine, University Medical Centre Ljubljana, Ljubljana); Zlatko Fras (Preventive Cardiology Unit, Department of Vascular Medicine, Division of Medicine, University Medical Centre Ljubljana, Ljubljana; Faculty of Medicine, University of Ljubljana, Ljubljana).

Republic of South Africa (505 patients enrolled) Henry P Cyster (TREAD Research CC, Parow); Naresh Ranjith (Nash Ranjith Research Centre, Durban); Clive Corbett (Panorma Medi-Clinic, Western Cape); Junaid Bayat (Suite 502, Durban Medical Centre, Durban); Ellen Makoali Makotoko and Hendrik du Toit Theron (Cardiology Research, Bloemfontein); Ilse E Kapp (Cardiology Clinical Research, Alberton); Matthys M de V Basson (Tiervlei Trial Centre, Bellville); Hanlie Lottering and Dina Van Aswegen (SCION Clinical Research, Pretoria); Louis J Van Zyl (Clinical Projects Research SA (Pty) Ltd, Worcester); Peter J Sebastian (Dr PJ Sebastian Private Practice, Chatsworth); Thayabran Pillay (Vincent Palotti Hospital, Cape Town); Jan A Saaiman (Kuils River Private Hospital, Kuilsrivier, Western Cape); Patrick J Commerford (Groote Schuur Hospital, Cape Town); Soraya Cassimjee and Garda Riaz (Dr Garda Cardiology Practice, Johannesburg); Iftikhar O Ebrahim (Unitas Hospital, Pretoria); Mahomed Sarvan (Drs MI Sarvan, R Moodley & Partners incorporated, Tongaat); Joseph H Mynhardt (Dr. J Mynhardt, Private Practice, Kimberley); Anthony J Dalby (Milpark Hospital, Johannesburg); Helmuth Reuter (Winelands Medical Research Centre, Stellenbosch); Rajendran Moodley (Netcare Umhlanga Medical Centre, Durban).

Spain (826 patients enrolled) Manuel Vida (Hospital Virgen del Mar, Almería, Almería); Angel R. Cequier Fillat (Hospital Universitari de Bellvitge, Hospitalet de Llobregat, Barcelona); Vicente Bodí Peris (Hospital Clínico Universitario, Valencia, Valencia); Francisco Fuentes Jimenez (Hospital Universitario Reina Sofía, Córdoba, Córdoba); Francisco Marín (Hospital Virgen De La Arrixaca, Murcia, Murcia); Jose M Cruz Fernández and Rafael Jesus Hidalgo Urbano (Hospital Universitario Virgen Macarena, Sevilla, Sevilla); Blas Gil-Extremera and Pablo Toledo (Complejo Hospitalario Universitario de Granada, Granada); Fernando Worner Diz (Hospital Universitari Arnau de Vilanova, Lleida, Lérida); David Garcia-

Dorado (Hospital Vall d'Hebron, Barcelona, Barcelona); Andres Iñiguez (Hospital Alvaro Cunqueiro, Vigo); José Tuñón Fernández (Fundación Jiménez Díaz, Madrid, Madrid); Jose R Gonzalez-Juanatey (Hospital Clínico Universitario de Santiago, Santiago de Compostela); Javier Fernandez Portales (Hospital San Pedro de Alcántara, Cáceres, Cáceres); Fernando Civeira Murillo (Hospital Miguel Servet, Zaragoza, Zaragoza); Laia Matas Pericas (Hospital de la Santa Creu i Sant Pau, Barcelona, Barcelona); Jose Luis Zamorano (Hospital Universitario Ramon y Cajal, Madrid, Madrid); Manuel De Mora Martin (Hospital Carlos Haya, Málaga, Málaga); Jordi Bruguera Cortada (Hospital Del Mar, Barcelona, Barcelona); Joaquin J Alonso Martin and Jose Maria Serrano Antolin (Hospital Universitario De Fuenlabrada, Fuenlabrada); José R De Berrazueta Fernández and José Antonio Vázquez de Prada (Hospital Universitario Marques De Valdecilla, Santander, Santander); Jose Francisco Díaz Fernández (Hospital Juan Ramón Jiménez, Huelva, Huelva); José Alberto García Lledó (Hospital Príncipe de Asturias, Alcalá de Henares, Madrid); Juan Cosín Sales (Hospital Arnau De Vilanova, Valencia, Valencia); Javier Botas Rodriguez (Hospital Universitario Fundación Alcorcón, Madrid, Madrid); Gabriel Gusi Tragant (Hospital de Sabadell - Corporacio Sanitaria Parc Tauli, Sabadell, Barcelona); Amparo Benedicto (Hospital Universitario la Princesa, Madrid, Madrid); Carlos Gonzalez-Juanatey (Hospital Universitario Lucus Augusti, Lugo, Lugo); Mercedes Camprubí Potau (Hospital Joan XXIII, Tarragona, Tarragona); Ignacio Plaza Perez (Hospital Universitario Infanta Sofia, San Sebastian de los Reyes); César Morís De La Tassa (Hospital Universitario Central de Asturias, Oviedo, Asturias); Pablo Loma-Osorio Rincon (Hospital Universitari de Girona Dr. Josep Trueta, Girona); Javier Balaguer Recena (Hospital General Universitario de Guadalajara, Guadalajara, Guadalajara); Juan M Escudier (Hospital Universitario Puerta De Hierro Majadahonda, Majadahonda); Antonio Coca Payeras (Hospital Clinic i Provincial, Barcelona); Norberto Alonso Orcajo (Complejo asistencial de Leon, Leon); Pedro Valdivielso (Hospital Virgen de la Victoria, Malaga).

Sri Lanka (314 patients enrolled) Godwin Constantine (University of Colombo, Colombo); Ruvaiz Haniffa (University of Colombo, Colombo); Nirmali Tissera (National Hospital of Sri Lanka, Colombo); Stanley Amarasekera and Chandrike Ponnamperuma (Colombo South Teaching Hospital, Kalubowila); Nimali Fernando and Kaputella Fernando (North Colombo Teaching Hospital, Ragama); Jayanthimala Jayawardena (Institute of Cardiology, National Hospital of Sri Lanka, Colombo); Santharaj Wijeyasingam and Gotabhaya Ranasinghe (Institute of Cardiology, National Hospital of Sri Lanka, Colombo); Ruvan Ekanayaka and Sepalika Mendis (Institute of Cardiology, National Hospital of Sri Lanka, Colombo); Sepalika Mendis (Institute of Cardiology, National Hospital of Sri Lanka, Colombo); Vajira Senaratne (Institute of Cardiology, National Hospital of Sri Lanka, Colombo); Gnanamoorthy Mayurathan (Kandy Teaching Hospital, Kandy); Thilak Sirisena (Kurunegala Teaching Hospital, Kurunegala); Ajantha Rajapaksha and Thilak Sirisena (Kurunegala Teaching Hospital, Kurunegala); Jagath I Herath (Sri Jayawardenapura General Hospital, Nugegoda); Naomali Amarasena (Sri Jayawardenapura General Hospital, Nugegoda).

Sweden (250 patients enrolled) Stefan Berglund (Falu Lasarett, Falun); Gundars Rasmanis (Karolinska University Hospital Huddinge, Stockholm); Emil Hagström and Ola Vedin (Dept of Medical Sciences, and Uppsala Clinical Research Centre, Uppsala University, Uppsala); Nils Witt (Södersjukhuset, Stockholm); Georgios Mourtzinis (Sahlgrenska University Hospital, Mölndal); Peter Nicol (Närsjukhuset Köping, Köping); Ole Hansen (Skåne University Hospital, Malmö); Stefano Romeo (Sahlgrenska University Hospital, Göteborg); Steen Agergaard Jensen (Blekingesjukhuset Karlshamn, Karlshamn); Ingemar Torstensson (Centralsjukhuset Kristianstad, Kristianstad); Steen Agergaard Jensen (Blekingesjukhuset Karlskrona, Karlskrona); Ulf Ahremark (Hallands sjukhus Halmstad, Halmstad); Torbjörn Sundelin (Länssjukhuset Sundsvall-Härnösand, Sundsvall).

Switzerland (88 patients enrolled) Tiziano Moccetti (Cardiocentro Ticino, Lugano); Christian Müller (Universitätsspital Basel, Basel); Francois Mach (Hopitaux Universitaires de Geneve, Geneve); Ronald Binde, Ulf Landmesser and Oliver Gämperli (Universitätsspital Zürich, Zürich).

Taiwan (93 patients enrolled) Chern-En Chiang (Taipei Veterans General Hospital, Taipei); Wei-Chuan Tsai (National Cheng Kung University Hospital, Tainan); Kwo-Chang Ueng (Chung-Shan Medical University Hospital, Taichung); Wen-Ter Lai (Kaohsiung Medical University Chung-Ho Memorial Hospital, Kaohsiung); Ming-En Liu (HsinChu Mackay Memorial Hospital, Hsinchu); Juey-Jen Hwang (National Taiwan University Hospital, Taipei); Wei-Hsian Yin (Cheng-Hsin General Hospital, Taipei); I-Chang Hsieh and Ming-Jer Hsieh (Chang Gung Memorial Hospital - Linkou, Taoyuan Hsien); Wei Hsiang Lin (Tri-

Service General Hospital - Taipei, Taipei); Jen-Yuan Kuo (Mackay Memorial Hospital, Taipei); Tsuei-Yuan Huang (Chi-Mei Medical Center, Tainan); Chih-Yuan Fang (Kaohsiung Chang Gung Memorial Hospital, Kaohsiung).

Thailand (161 patients enrolled) Pinij Kaewsuwanna (Maharat Nakhonratchasima Hospital, Moung); Wasant Soonfuang (Police General Hospital, Pratumwan); Woravut Jintapakorn (Songklanagarind Hospital, Hat Yai); Apichard Sukonthasarn (Chiang Mai University, Muang, Chiang Mai); Piyamitr Sritara (Ramathibodi Hospital, Bangkok); Nattawut Wongpraparut (Siriraj Hospital, Mahidol University, Bangkok-Noi); Krisada Sastravaha (Bhumibol Adulyadej Hospital, Bangkok); Nakarin Sansanayudh (Phramongkutklao Hospital, Bangkok); Wirash Kehasukcharoen (Central Chest Institute, Nonthaburi); Dilok Piyayotai (Thammasat University Hospital, Klong Luang); Paiboon Chotnoparatpat (Vajira Bangkok University Hospital, Bangkok).

Turkey (78 patients enrolled) Ahmet Camsari (Mersin Universty School Of Medicine Department Of Cardiology, Mersin); Hakan Kultursay (Ege University Medical Faculty Department of Cardiyology, IZMIR); Sema Guneri (Dokuz Eylul Universitesi Tip Fakultesi, Izmir); Bulent Mutlu (Marmara Universitesi Pendik Egitim ve Arastirma Hastanesi, Istanbul); Murat Ersanli (Istanbul Universitesi Kardiyoloji Enstitusu, Istanbul); Mustafa Demirtas (Cukurova Universitesi Tip Fakultesi, Adana); Cevat Kirma (Kartal Kosuyolu Yuksek Ihtisas Egitim ve Arastirma Hastanesi, Istanbul); Ertan Ural (Kocaeli Universitesi Tip Fakultesi, Kocaeli); Lale Koldas (Istanbul Universitesi Cerrahpasa Tip Fakultesi, Istanbul).

Ukraine (639 patients enrolled) Oleksandr Karpenko (City Clinical Hospital No 1, Kiev); Alexander Prokhorov (City Clinical Hospital #27, Kharkiv); Ihor Vakaluyk (Ivano-Frankivsk Regional Clinical Cardiology Center, Ivano-Frankivsk); Halyna Myshanych (Railway Clinical Hospital #2, "Health Center", Kyiv); Dmytro Reshotko (Kyiv City Oleksandrivska Clinical Hospital, Kyiv); Valeriy Batushkin (Kyiv City Clinical Hospital #5, Cardiology Department, Kyiv); Leonid Rudenko (Kyiv Emergency Care Hospital, Kiev); Ihor Kovalskyi (City Hospital #1, Mykolaiv); Mykola Kushnir (Zhytomyr regional clinical hospital n.a. O.F. Gerbachevskyy, Zhytomyr); Vira Tseluyko (City Clinical Hospital #8, Kharkiv); Yuriy Mostovoy (Vinnitsya City Clinical Hospital 1, Vinnitsya); Mykola Stanislavchuk (Vinnytsia M.I. Pyrohov Regional Clinical Hospital, Vinnytsia); Yulian Kyiak (Lviv Danylo Halytskyi National Medical University, Lviv); Yuriy Karpenko (Odesa Regional Clinical Hospital, Odesa); Yaroslav Malynovsky (Regional Medical Center for Cardiovascular Diseases, Zaporizhia); Andriy Klantsa (Khmelnytskyi Regional Hospital, Khmelnytskyi); Oles Kutniy (Public Institution: Central City Hospital #2, Zhytomyr); Ekaterina Amosova (Kyiv City Oleksandrivska Clinical Hospital, Kyiv); Viktor Tashchuk (Chernivtsi Regional Clinical Cardiology Center, Chernivtsi); Oleh Leshchuk (Lviv Regional Clinical Hospital, Dept. of Internal Medicine, Lviv); Alexander Parkhomenko (“M.D. Strazhesko Institute of Cardiology of AMS of Ukraine”, Kiev); Mykola Rishko (Zakarpattya Regional cardiology Dispensary, Uzhgorod); Mykola Kopytsya (State Institution LT Malaya Inst. of Ther. of AMS of Ukraine, kharkiv); Andriy Yagensky (Lutsk City Clinical Hospital, Lutsk); Mykola Vatutin (Donetsk National Medical University named after M. Gorkiy, Donetsk); Andriy Bagriy (Donetsk National Medical University named after M. Gorkiy, Donetsk); Olga M Barna (Policlinic ofadm of med services and rehabilitation of ARTEM, Kyiv); Olexiy Ushakov (City Clinical Hospital #6 of Emergency Medical Care, Simferopol); Georgiy Dzyak (Dnipropetrovsk Regional Clinical Center of Cardiology, Dnipropetrovsk); Borys Goloborodko (City Clinical Hospital # 3, Odessa); Anatolii Rudenko (M.M. Amosov National Institute of Cardiovascular Surgery, Kyiv); Volodymyr Zheleznyy (Sumy City Clinical Hospital #1, Sumy).

United Kingdom (292 patients enrolled) Jasper Trevelyan (Worcestershire Royal Hospital, Worcester, Worcestershire); Azfar Zaman (Freeman Hospital, Newcastle Upon Tyne, Tyne and Wear); Kaeng Lee (Birmingham Heartlands Hospital, Birmingham); Andrew Moriarty (Craigavon Area Hospital, Portadown, Armagh); Rajesh K Aggarwal (Basildon and Thurrock University Hospitals, Basildon, Essex); Piers Clifford (Wycombe General Hospital, High Wycombe, Buckinghamshire); Yuk-Ki Wong (St Richard's Hospital, Chichester, West Sussex); Syed MR Iqbal (Surrey and Sussex Healthcare NHS Trust, Redhill); Eduardas Subkovas (Glan Clwyd Hospital, Rhyl, Denbighshire); Denise Braganza (Peterborough and Stamford Hospitals NHS Foundation Trust, Peterborough); David Sarkar (Plymouth Hospitals NHS Trust, Plymouth, Devon); Robert Storey (University of Sheffield, Sheffield, South Yorkshire); Huw Griffiths (Queen Alexandra Hospital, Portsmouth, Hampshire); Sam Mcclure (City Hospitals Sunderland NHS Foundation Trust, Sunderland); Rangasamy Muthusamy and Simon Smith (The Rotherham NHS Foundation Trust, Rotherham, South Yorkshire); John Kurian (Bradford Teaching Hospitals NHS

Foundation Trust, Bradford); Terry Levy (Royal Bournemouth Hospital, Bournemouth, Dorset); Craig Barr (The Dudley Group of Hospitals NHS Foundation Trust, Dudley, West Midlands); Honer Kadr (Queen's Hospital, Romford, Essex); Robert Gerber (Conquest Hospital, St Leonards, East Sussex); Audrius Simaitis (Royal Cornwall Hospitals NHS Trust, Truro, Cornwall); Handrean Soran (St Marys Hospital, Manchester, Greater Manchester); Anthony Mathur (London Chest Hospital, London); Adrian Brodison, Mohammad Ayaz and Muhammad Cheema (University Hospitals of Morecambe Bay NHS Foundation Trust, Cumbria, Cumbria); Richard Oliver and Simon Thackray (Castle Hill Hospital Hull And East Yorkshire Trust, Hull, East Yorkshire); Telal Mudawi, Gohar Rahman, and Ayyaz Sultan (Wrightington, Wigan and Leigh NHS Foundation Trust, Wigan, Greater Manchester); Timothy Reynolds (Queen's Hospital, Burton On Trent, Staffordshire); David Sharman and david Sprigings(Northampton General Hospital, Northampton, Northamptonshire); Rob Butler (University Hospital Of North Staffordshire, Stoke-On-Trent, Staffordshire); Peter Wilkinson (Ashford & St Peter's Hospitals NHS Foundation Trust, Chertsey); Gregory YH Lip (City Hospital, Birmingham, West Midlands); Julian Halcox, Sean Gallagher and Nicholas Ossei-Gerning(University Hospital Of Wales, Cardiff, South Glamorgan).

United States (2511 patients enrolled) Gil Vardi (St. Louis Heart and Vascular, St. Louis, Missouri); Duccio Baldari (Cardiology Partners Clinical Research Institute, Wellington, Florida); David Brabham (PharmaTex Research, Amarillo, Texas); Charles Treasure II (Cardiovascular Research of Knoxville, Knoxville); Charles Dahl (Aspen Clinical Research, Orem, Utah); Bruce Palmer (Wichita Falls Heart Clinic, Wichita Falls, Texas); Alan Wiseman (EMMC Northeast Cardiology Associates, Bangor); Abul Khan (The Iowa Clinic, West Des Moines, Iowa); Sanjeev Puri (Quad City Heart Center, Moline, Illinois); Ann Elizabeth Mohart (Mercy Health Research, Washington, Missouri); Carlos Ince (Maryland Cardiovascular Specialists, Baltimore, Maryland); Enrique Flores (Georgia Heart Specialists, LLC, Covington, Georgia); Scott Wright (Cardiovascular Associates of East Texas, Tyler, Texas); Shi-Chi Cheng (The Heart Center, PC, Huntsville, Alabama); Michael Rosenberg (Heart and Vascular Center/Holy Family Memorial, Manitowoc, Wisconsin); William Rogers Jr (University of Alabama at Birmingham Medical Center, Birmingham); Edward Kosinski (Connecticut Clinical Research, LLC, Bridgeport); Les Forgosh (HealthEast Medical Research Institute, St. Paul); Jonathan Waltman (Saint Joseph's Healthcare Research Center, Lexington, Kentucky); Misal Khan (Springfield Medical Center, Panama City); Mohammad Shoukfeh (Caprock Cardiac Center Research Institute, Lubbock); Georges Dagher and Patrick Cambier (Morton Plant Mease Health Care, Inc., Clearwater, Florida); Ira Lieber (TCR Institute, LLC, Kingwood, Texas); Priya Kumar (Advanced Research Associates, Greenwood, South Carolina); Cara East (Baylor University Medical Center, Dallas, Texas); Perry Krichmar and Mian Hasan (Research Physicians Network Alliance, Pembroke Pines, Florida); Lindsey White (Eastern Carolina Cardiovascular Associates, Elizabeth City, North Carolina); Thomas Knickelbine (Minneapolis Heart Institute Foundation, Minneapolis, Minnesota); Thomas Haldis (Sanford-Fargo, Fargo, North Dakota); Eve Gillespie and Thomas Amidon (Glacier View Cardiology PC, Kalispell, Montana); David Suh (Atlanta Heart Specialists LLC, Tucker); Imran Arif and mouhamad Abdallah (University of Cincinnati Hospital, Cincinnati); Faiq Akhter (Apex Medical Research, AMR Inc., Springfield, Ohio); Eric Carlson (Eastern Cardiology, P.A., Greenville, North Carolina); Michael D'Urso (Black Hills Cardiovascular Research, Rapid City, South Dakota); Fadi El-Ahdab (The Cardiovascular Group Centra, Lynchburg, Virginia); William Nelson and Katie Moriarty (Cardiology Research, St. Paul, Minnesota); Barry Harris (Integrative Research Associates Inc, Fort Lauderdale, Florida); Steven Cohen (The Cardiology Center, Delray Beach); Luther Carter and Daniel Doty (Cardiology Consultants, Pensacola); Kenneth Sabatino (Clearwater Cardiovascular Consultants, Safety Harbor, Florida); Tariq Haddad (Virginia Heart, Leesburg, Virginia); Amir Malik (Plaza Medical Center of Fort Worth, Fort Worth); Sunder Rao (Butler Medical Providers, Butler); Angel Mulkay (Mulkay Cardiology Consultants PC, Hackensack, New Jersey); Ion Jovin (McGuire V.A. Medical Center, Richmond, Virginia); Kim Klancke (Daytona Heart Group, Daytona Beach); Vinay Malhotra (MultiCare Institute for Research and Innovation, Puyallup); Sai K Devarapalli (IU Health Ball Memorial Hospital Physicians, Inc., Muncie, Indiana); Michael Koren (Jacksonville Center for Clinical Research, Jacksonville); Harish Chandna (Victoria Heart and Vascular Center, Victoria, Texas); George Dodds III, Tauqir Goraya and James Bengston (Michigan Heart, PC, Ypsilanti, Michigan); Matthew Janik (PMG Research of Wilmington, Wilmington, North Carolina); Joseph Moran (Piedmont Healthcare - Research, Statesville, North Carolina); Andrew Sumner (Lehigh Valley Hospital/Lehigh Valley Health Network, Allentown, Pennsylvania); John Kobayashi (Beacon Medical Group, South Bend, Indiana); William Davis (Advanced Cardiovascular, LLC, Auburn, Alabama); Shahram Yazdani (Inova

Cardiology Ambulatory Research(ICARE-1), Manassas, Virginia); John Pasquini (Novant Health Heart and Vascular Institute, Charlotte, North Carolina); Maitreya Thakkar (PMG Research of Rocky Mount, LLC, Rocky Mount, North Carolina); Amarnath Vedere (Cardiology Partners Clinical Research Institute, Palm Beach Gardens); Wayne Leimbach (Oklahoma Heart Institute, Tulsa); James Rider and Sarah fenton (Cardiology Associates of Bellin Health, Green Bay); Narendra Singh (Atlanta Heart Specialists, Cumming); Anil V Shah (Coastal Multi-Specialty Research, Santa Ana, California); Patrick M. Moriarty (Clinical Pharmacology – University of Kansas Medical Center, Kansas City, Kansas); Denise Janosik (Saint John's Mercy Cardiovascular Research, St. Louis, Missouri); Carl Pepine (University of Florida, Gainesville, Florida); Brett Berman (Brett J. Berman, MD, APC, Chula Vista Cardiac Center, California); Joseph Gelormini (Trinity Medical WNY, PC, Buffalo, New York); Christopher Daniels and Kerensky Richard (John D. Archbold Memorial Hospital, Thomasville, Georgia); Friederike Keating (The University of Vermont - Fletcher Allen Health Care, Burlington, Vermont); Nicholas I Kondo (Saratoga Cardiology Associates, Saratoga Springs, New York); Sanjay Shetty and Howard Levite (AtlantiCare Regional Medical Center, Pomona); Winfried Waider (Long Beach Memorial Medical Center, Long Beach, California); Theodore Takata (Texas Health Research and Education Institute, Fort Worth, Texas); Mazen Abu-Fadel (University of Oklahoma Health Sciences Center, Oklahoma City); Vipul Shah (Carolina Heart Specialists, LLC, Lancaster, South Carolina); Rahul Aggarwal (Tenet Florida Physician Services, Jupiter); Mark Izzo (St. Vincent Consultants in Cardiovascular LLC, Erie, Pennsylvania); Anil Kumar (Kumar Medical Corporation, Lancaster, California); Brack Hattler and Rose Do (Denver V.A. Medical Center, Denver); Chad Link (Sparrow Clinical Research Institute, Lansing); Anna Bortnick (Montefiore Medical Center, Bronx, New York); George Kinzfogl III (Metrowest Medical Center, Framingham, Massachusetts); Arnold Ghitis (Heart and Health Institute Westside, Plantation, Florida); John Larry (Ohio State University Hospital East, Columbus, Ohio); Edward Teufel (Main Medical Partners Maine Health Cardiology, Scarborough, Maine); Peter Kuhlman (Saint Vincent's Cardiology, Jacksonville); Brent Mclaurin (AnMed Health Medical Center, Anderson); Wenwu Zhang (Cardiovascular Solutions, LLC, Shreveport, Louisiana); Stephen Thew (Heart Clinics Northwest, Spokane, Washington); Jalal Abbas (Clinical Research Institute of Arizona, Surprise); Matthew White, Othman Islam, Matthew White and Sumeet Subherwal (Raleigh Cardiology Associates, P.A., Raleigh); Nandkishore Ranadive and Babak Vakili (Orlando Heart Specialists, Altamonte Springs); Christian Gring (NC Heart and Vascular Research, Clayton, North Carolina); David Henderson (Cardiology Research Associates, Daytona Beach); Timothy Schuchard (CentraCare Heart & Vas Center at St. Cloud Hospital, St. Cloud, Minnesota); Naim Farhat (North Ohio Research, Ltd., Elyria, Ohio); Geoffrey Kline (U of N Texas Health Science Ctr-Infectious Disease, Fort Worth, Texas); Sharan Mahal (Advanced Heart Care, LLC, Bridgewater, New Jersey); Jack Whitaker (Wellmont CVA Heart Institute, Greenville, Tennessee); Shawn Speirs (Eastern Idaho Regional Medical Center, Idaho Falls); Rolf Andersen (Lancaster Heart Foundation, Lancaster, Pennsylvania); Nizar Daboul (Advanced Medical Research, Maumee); Phillip Horwitz and Firas Zahr (University of Iowa, Iowa City); George Ponce (Spectrum Clinical Research Institute Inc, Moreno Valley, California); Zubair Jafar (Hudson Valley Heart Center, Poughkeepsie); Joseph Mcgarvey Jr (Doylestown Cardiology Associates-VIAA, Doylestown, Pennsylvania); Vipul Panchal (Norton Cardiovascular Associates, Louisville, Kentucky); Stephen Voyce (Geisinger Cardiology-Scranton, Scranton, Pennsylvania); Thomas Blok (Advanced Cardiac Healthcare, Kalamazoo); William Sheldon (North Ohio Research Center - Sandusky, Sandusky, Ohio); Masoud M Azizad (Valley Clinical Trials, Inc., Northridge); Carsten Schmalfuss (Malcom Randall V.A. Medical Center, Gainesville); Mark Picone and Robert Pederson (Austin Heart, PLLC, Austin, Texas); William Herzog Jr and Keith Friedman (CV Specialists of Central Maryland/Johns Hopkins University, Columbia, Maryland); Jason Lindsey (Saint Luke's Hospital, Kansas City, Missouri); Rosemary Nowins, Eichenlaub Timothy and Parilak Leonard (CIMA Medical Resarch Inc, Las Vegas, Nevada); Norman Lepor (Westside Medical Associates of Los Angeles, Beverly Hills); Mahfouz El Shahawy (Cardiovascular Center of Sarasota, Sarasota, Florida); Howard Weintraub (New York University Medical Center, New York); Anand Irimpen and Alvaro Alonso (Tulane University Medical Center, Dept. of Medicine, New Orleans); Wade May and Daniels Christopher (Cardiovascular Institute of the South, Lafayette, Louisiana); Thomas Galski (Virtua the Cardiology Group, Moorestown, New Jersey); Alan Chu (Amarillo Heart Clinical Research Institute, Inc., Amarillo, Texas); Freny Mody and Ebrahimi Ramin (V.A. Greater Los Angeles Health Care System at West LA, Los Angeles, California); Zachary Hodes and Joseph Rossi (The Care Group LLC, Indianapolis, Indiana); Gregory Rose (Wake Heart Research, Raleigh, North Carolina); James Fairlamb (Missouri Cardiovascular Specialists, Columbia, Missouri); Charles Lambert Jr (Florida Hospital Pepin Heart Institute, Tampa, Florida); Ajit Raisinghani

(University of California at San Diego, San Diego, California); Antonio Abbate and George Vetrovec (Virginia Commonwealth University Medical Center, Richmond); Marilyn King (Clearwater CV and Interventional Consultants- Bardmoor, Largo, Florida); Charles Carey (Saint Anthony's Medical Center, St. Louis, Missouri); Jaime Gerber (Yale New Haven Hospital Heart and Vascular Research, North Haven, Connecticut); Liwa Younis (Gateway Cardiovascular Research, St. Louis, Missouri); Hyeun (Tom) Park (Lakeland Cardiology, Mountain Lakes, New Jersey); Mladen Vidovich (University of Illinois at Chicago, Chicago, Illinois); Thomas Knutson (Prevea Clinic, Inc., Green Bay); Dennis Friedman (Cardiac Associates, PC, Rockville, Maryland); Fred Chaleff (Infinity Clinical Research LLC, Sunrise, Florida); Arthur Loussararian (St. Jude Hospital Yorba Linda DBA St. Joseph Heritage Health, Mission Viejo, California); Phillip Rozeman (Clinical Trials of America, Minden, Louisiana); Carey Kimmelstiel and Jeffrey Kuvin (Tufts-New England Medical Center, Boston); Kevin Silver (Summa Health, Akron, Ohio); Malcolm Foster (Turkey Creek Medical Center, Knoxville, Tennessee); Glen Tonnessen and Andrey Espinoza (Hunterdon Cardiovascular Associates, Flemington, New Jersey); Mohamadali Amlani (Mid-Michigan Cardiology Associates, Flint, Michigan); Andreas Wali (Holy Spirit Cardiology, Camp Hill, Pennsylvania); Christopher Malozzi, Geert T Jong and Clara Massey (University of South Alabama Medical Center, Mobile, Alabama); Keattiyoat Wattanakit (Heartcare Midwest, Peoria); Philip J. O'Donnell (Selma Medical Associates, Winchester); Dinesh Singal (Cardio Metabolic Institute, Somerset); Naseem Jaffrani (Alexandria Cardiology Clinic, Alexandria); Sridhar Banuru (The Heart Group, Newburgh); Daniel Fisher (Capitol Interventional Cardiology, Carmichael, California); Mark Xenakis (Cardiovascular Associates of Virginia, Midlothian, Virginia); Neal Perlmutter (Overlake Medical Clinics, Bellevue, Washington); Ravi Bhagwat (Cardiovascular Research of Northwest Indiana, Munster, Indiana); James Strader Jr and Ronald Blonder (Colorado Health Medical Group CV and Thoracic Care, Colorado Springs, Colorado); Ayim Akyea-Djamson (Metropolitan Cardiovascular Consultants, Beltsville, Maryland); Ajay Labroo (Advanced CardioVascular Consultants, Rock Island); Kwan Lee (University of Arizona Medical Center, Tucson, Arizona); H. John Marais (Beaver Medical Clinic, Banning, California); Edmund Claxton, Jr, Robert Weiss and Rohr Kathryn (Maine Research Associates, Auburn); Martin Berk (CV Research Institute of Dallas, Inc., Dallas, Texas); Peter Rossi (Pasco Cardiology Center, Hudson); Parag Joshi and Amit Khera (UT Southwestern Medical Center, Dallas); Ajit S Khaira (Bala and Khaira Medical Research Center, Fresno); Greg Kumkumian (Maryland Heart, PC, Bethesda, Maryland); Steven Lupovitch (Northwest Heart Clinical Research, Arlington Heights, Illinois); Joshua Purow (Holy Cross Hospital Inc, Fort Lauderdale, Florida); Stephen Welka (Aurora Memorial Hospital Burlington, Burlington, Wisconsin); David Hoffman (St. Elizabeth Health Center, Youngstown, Ohio); Stuart Fischer (Los Alamitos Cardiovascular, Los Alamitos); Eugene Soroka (Saviers Medical Group, Port Hueneme, California); Donald Eagerton (Carolina Health Specialists, Myrtle Beach, South Carolina); Samir Pancholy (Northeast Clinical Trials Group, Scranton); Michael Ray and Norman Erenrich (Cardiology Associates of Palm Beach, West Palm Beach); Michael Farrar (Northland Cardiology, North Kansas City); Stewart Pollock (Harrisonburg Medical Association, Harrisonburg, Virginia); William J French (Harbor University of California Los Angeles Medical Center, Torrance, California); Steve Diamantis and Douglas Guy (Methodist Physicians Clinic – Heart Consultants, Omaha); Lawrence Gimple (University of Virginia Health System, Charlottesville, Virginia); Mark Neustel (St. Mary's/Duluth Clinic, Duluth, Minnesota); Steven Schwartz (Foundation Cardiology, Nashua, New Hampshire); Edward Pereira and Seals Albert (East Coast Institute for Research, Jacksonville); Douglas Spriggs (Clearwater Cardiovascular Consultants, Clearwater); Janet Strain and Suneet Mittal (Valley Health System, Ridgewood, New Jersey); Anthony Vo (Long Beach V.A. Healthcare System, Long Beach, California); Majed Chane (California Heart Specialists, Huntington Beach); Jason Hall (Medicore Cardiology, Bridgewater, New Jersey); Nampalli Vijay (Aurora Denver Cardiology Associates, PC, Denver); Kapildeo Lotun (University of Arizona Sarver Heart Center, Tucson, Arizona); F. Martin Lester (IMC Diagnostic and Medical Clinic, Mobile, Alabama); Ahed Nahhas (Toledo Clinic, Toledo); Theodore Pope (Midwest Heart and Vascular Specialists, Overland Park, Kansas); Paul Nager (Midwest Heart & Vascular Specialists, Independence, Missouri); Rakesh Vohra and Mukesh Sharma (Parkway Cardiology Associates, PC, Oak Ridge); Riyaz Bashir (Temple University Hospital, Philadelphia); Hinan Ahmed (The University of Texas Health Science Center at San Antonio, San Antonio, Texas); Michael Berlowitz (Tampa General Hospital, Tampa, Florida); Robert Fishberg (Associates in Cardiovascular Disease, LLC, Springfield, New Jersey); Robert Barrucco (Virtua Cardiology Group, Voorhees, New Jersey); Eric Yang (University of California at Los Angeles, Los Angeles, California); Michael Radin (Radin Cardiovascular Medical Group, Inc, Newport Beach); Daniel Sporn and Dwight Stapleton (Guthrie Clinic, Ltd., Sayre, Pennsylvania); Steven Eisenberg

(Cardiovascular Specialists, PC, Atlanta, Georgia); Joel Landzberg (Westwood Cardiology, Westwood, New Jersey); Martin Mcgough (Academic Cardiology Associates, Rochester Hills, Michigan); Samir Turk (Trinity Medical Group, Minot); Michael Schwartz (DuPage Medical Group Cardiology, Winfield, Illinois); P. Sandy Sundram (Advanced Heart Group, Harvey); Diwakar Jain (Westchester Medical Center, Valhalla); Mark Zainea (McLaren Macomb, Mount Clemens, Michigan); Carlos Bayron (Interventional Cardiac Consultants, Trinity, Florida); Ronald Karlsberg and Suhail Dohad (Cardiovascular Research Foundation of Southern California, Beverly Hill, California); Henry Lui (Research Associates of Jackson, Jackson); William Keen (Kaiser Permanente Viewridge Medical Offices, San Diego, California); Donald Westerhausen Jr. (Midwest Cardiovascular Research, Elkhart, Indiana); Sandeep Khurana (Healthy Heart Cardiology, Grandville); Himanshu Agarwal (Allegent Health Heart and Vascular Specialsts, Omaha, Nebraska); Jessica Birchem (Mercy Medical Research Institute, Springfield); William Penny Jr (V.A. San Diego Health Care System, San Diego, California); Mark Chang (Wellmont CVA Heart Institute, Johnson City); Sherrill Murphy and John Henry (Heart Consultants PC, Omaha, Nebraska); Michael Ray (Cardiology Associates of Palm Beach, Atlantis, Florida); Branislav Schifferdecker (Oklahoma Heart Hospital, Oklahoma City, Oklahoma City);John M Gilbert (Saint Jude Heritage Medical Group, Fullerton, California); Gopal Chalavarya (Florida Cardiology Group, Hudson, Florida); Charles Eaton (Memorial Hospital of Rhode Island, Pawtucket, Rhode Island); John F Schmedtje Jr (Roanoke Heart Institute, Roanoke); Stuart Christenson and Imran Dotani (McFarland Clinic PC, Ames, Iowa); Douglas Denham (Clinical Trials of Texas, Inc., San Antonio); Alexander Macdonell (University Cardiology Associates, Augusta, Georgia); Paul Gibson (Cardiology Research Associates, Saint Louis); Aref Rahman (V.A. Pittsburgh Healthcare System, Pittsburgh); Tammam Al Joundi and Nizar Assi (Gateway Cardiovascular Research Center, Jerseyville, Illinois); Gary Conrad (Huntington Memorial Hospital, Pasadena, California); Purushotham Kotha (Purushotham and Akther Kotha MD, Inc, La Mesa, California); Michael Love (Erlanger Health System, Chattanooga, Tennessee); Gregory Giesler (Huntington Hospital, Pasadena, California); Howard Rubenstein and Dawood Gamil (Port City Clinical Research, Saraland, Alabama); Laura Akright (Northeast Clinical Research of San Antonio, Schertz, Texas); Branislav Schifferdecker (Oklahoma Heart Hospital, Oklahoma City, OK); Justine Krawczyk and Joanne Cobler (Buffalo Medical Group, Williamsville, New York); Terry Wells (Angelina Medical Research LLC, Lufkin, TX); James Welker (Anne Arundel Health System, Annapolis, MD); Robert Foster (Birmingham Heart Clinic, PC, Birmingham, AL); Richard Gilmore (Lake Charles Medical and Surgical Clinic, Lake Charles, LA); Jay Anderson (Nebraska Medicine - Internal Medical Associates,
Grand Island, NE); Douglas Jacoby (Pennsylvania Presbyterian Medical Center, Philadelphia, PA); Bill Harris (Pikeville Medical Center, Pikeville, KY); Geraldine Gardner (Providence Everett Medical Center, Everett, WA); Ramprasad Dandillaya (Ram Dandillaya, M.D., Inc, Beverly Hills, CA); Kishor Vora (Research Integrity, Owensboro, KY); John Kostis (Rutgers University - Robert Wood Johnson Medical School, New Brunswick, NJ); John Hunter (Santa Rosa Sebastopol Cardiology Group, Santa Rosa, CA); David Laxson (University of Minnesota Heart Care, Edina, MN); Eric Ball (Walla Walla Clinic, Walla Walla, WA); Jay Anderson (Internal Medical Association, Grand Island, Nebraska); David Laxson (Minnesota Heart Clinic, Edina, Minnesota); John Kostis (UMDNJ - Robert Wood Johnson Medical Center, New Brunswick, New Jersey); Robert Foster (Birmingham Heart Clinic, Birmingham, Alabama); Richard Gilmore (Lake Charles Medical and Surgical Clinic, Lake Charles, Louisiana); Terry Wells (TAD Clinical Research, Lufkin,Texas); John Hunter (Santa Rosa Cardiology Medical Group, Santa Rosa, California); Kishor Vora (Research Integrity, Owensboro, Kentucky); Eric Ball (Walla Walla Clinic, Walla Walla, Washington); Bill Harris (Pikeville Medical Center, Pikeville, Kentucky); Geraldine Gardner (Providence Everett Medical Center, Everett, Washington); Douglas Jacoby (Pennsylvania Presbyterian Medical Center, Philadelphia, Pennsylvania); James Welker (Anne Arundel Health System, Annapolis, Maryland); Ramprasad Dandillaya (Ram Dandilaya, Inc., Beverly Hills, California).

Academic Research Organizations and Contract Research Organizations

Brazilian Clinical Research Institute, São Paulo, Brazil
Renato Lopes, Flavia Egydio, Anelise Kawakami, Janaina Oliveira.
Canadian VIGOUR Centre, University of Alberta, Edmonton, Canada
Shaun G. Goodman, Julianna Wozniak.
Covance, Marlow, Buckinghamshire, UK
Alexander Matthews, Caroline Ratky, Janine Valiris.

Duke Clinical Research Institute, Durham, NC, USA
Lisa Berdan, Anita Hepditch, Kirby Quintero, Matthew T. Roe, Tyrus Rorick, Melissa Westbrook.
Estudios Clínicos Latino America, Rosario, Santa Fe, Argentina
Rafael Diaz, Andrea Pascual, Carla Rovito.
French Alliance for Cardiovascular Trials (FACT), an F-CRIN network, Paris, France
Nicolas Danchin, Madeleine Bezault, Elodie Drouet, Tabassome Simon.
Green Lane Coordinating Centre, Kingsland, Auckland, New Zealand
Harvey D. White, Caroline Alsweiler.
Leuven Klinisch Coördinatiecentrum, Leuven, Belgium
Peter Sinnaeve, Anne Luyten.
South Australian Health & Medical Research Institute
Phil Aylward, Julie Butters, Liddy Griffith, Michelle Shaw.
Uppsala kliniska forskningscentrum, Uppsala, Sweden
Emil Hagstrom, Lena Grunberg.

Independent Academic Statisticians: SUNY Downstate School of Public Health
Michael Szarek, Shahidul Islam.

Sponsors
Medical Operations
Marie-France Brégeault, Nathalie Bougon, Douglas Faustino, Sylvie Fontecave, Judith Murphy, Jean-Francois Tamby, Melanie Verrier **(Sanofi, France).**

Trial Operations*
Veronique Agnetti, Dorthe Andersen, Emmy Badreddine, Mhamed Bekkouche, Cecile Bouancheau, Imane Brigui, Maddy Brocklehurst, Joseph Cianciarulo, Dawn Devaul, Szilvia Domokos, Cecile Gache, Caroline Gobillot, Severine Guillou, Jan Healy, Megan Heath, Gayatri Jaiwal, Carine Javierre, Julien Labeirie, Myriam Monier, Ulises Morales, Asmaa Mrabti, Bicky Mthombeni, Betim Okan, Lucile Smith, Jennifer Sheller, Sebastien Sopena, Valerie Pellan (*Sanofi, Paris, France*).

**Excluding North American countries, which were managed by Duke Clinical Research Institute (see above).*

Pharmacovigilance
Fadela Benbernou, Nafissa Bengrait, Maud Lamoureux, Katarina Kralova, Michel Scemama **(Sanofi, Paris, France).**

Statisticians
Raphael Bejuit, Anthony Coulange, Christelle Berthou, Jérôme Repincay, Christelle Lorenzato, Alexis Etienne, Valerie Gouet, Guillaume Lecorps, Virginie Loizeau, Mickael Normand, Anne Ourliac, Christelle Rondel (**Sanofi, Paris, France).**

Investigational Product Management
Antony Adamo, Pascale Beltran, Pauline Barraud, Helene-Dubois-Gache, Benjamin Halle, Lamia Metwally, Maxime Mourgues, Marc Sotty, Marion Vincendet **(Sanofi, Paris, France)**.

Clinical Documentation
Raluca Cotruta, Zhu Chengyue, Dominique Fournie-Lloret, Christine Morrello, Aurelie Perthuis, Patrick Picault, Isabelle Zobouyan **(Sanofi, France)**.

Diabetes Expert Review Committee
Helen M. Colhoun, University of Edinburgh, Edinburgh, Scotland, UK.
Michael A. Dempsey, Endocrine & Metabolic Consultants, Rockville, MD, USA.
Mark A. McClanahan, Diabetes and Thyroid Associates, PC, Fredericksburg, USA.

Table I Standardized mean differences of factors used in propensity score matching

Month 4 LDL-C in alirocumab group	<25 mg/dL		25-50 mg/dL		>50 mg/dL	
	All Eligible Patients (n=12601)	Matched Patients (n=6714)	All Eligible Patients (n=12936)	Matched Patients (n=7392)	All Eligible Patients (n=11441)	Matched Patients (n=4394)
Age	n/a	n/a	n/a	n/a	-0.038	0.017
Female sex	-0.109	-0.014	n/a	n/a	0.171	-0.019
Western Europe	0.045	-0.005	-0.095	-0.004	0.106	0.001
Eastern Europe	0.065	0.008	0.034	-0.008	-0.154	0.032
North America	0.046	0.016	-0.018	-0.005	-0.038	0.013
South America	-0.081	-0.015	0.014	-0.002	0.114	0.013
Asia	-0.131	-0.007	0.089	0.020	0.081	-0.057
Heart failure	0.064	0.003	0.060	-0.037	n/a	n/a
Intensive statin	0.060	-0.005	-0.041	0.006	n/a	n/a
Revascularization for index ACS	n/a	n/a	n/a	n/a	0.103	-0.031
Body mass index	n/a	n/a	n/a	n/a	0.069	-0.009
Systolic blood pressure	0.009	0.001	0.042	-0.004	n/a	n/a
LDL-C	-0.374	<0.001	n/a	n/a	0.447	0.002
Lp(a)	-0.256	<0.001	0.112	0.013	0.119	-0.012
≥4 doses of study medication	-0.242	0.008	-0.140	0.006	0.418	0.024

All eligible patients include patients in the specified achieved LDL-C stratum of the alirocumab group and all analysis-eligible patients from the placebo group. Matched patients include patients in the specified achieved LDL-C stratum of the alirocumab group and propensity score-matched patients from the placebo group.

n/a = not applicable, because not used in propensity score matching for given achieved LDL-C subgroup

Table II Protocol-specified adjustments of alirocumab dose

**Type of protocol-specified adjustment of alirocumab dose after month 4**	**Number of Patients**
Switch to placebo	717
Up-titration, down-titration, and switch to placebo	5
Up-titration and down-titration	21
Up-titration	79

Figure I Consort Diagram. From Schwartz GG, Steg PG, Szarek M, et al. Alirocumab and cardiovascular outcomes after acute coronary syndrome. *N Engl J Med* 379(22):2097-2107.12 Copyright © (2018) Massachusetts Medical Society. Reprinted with permission.

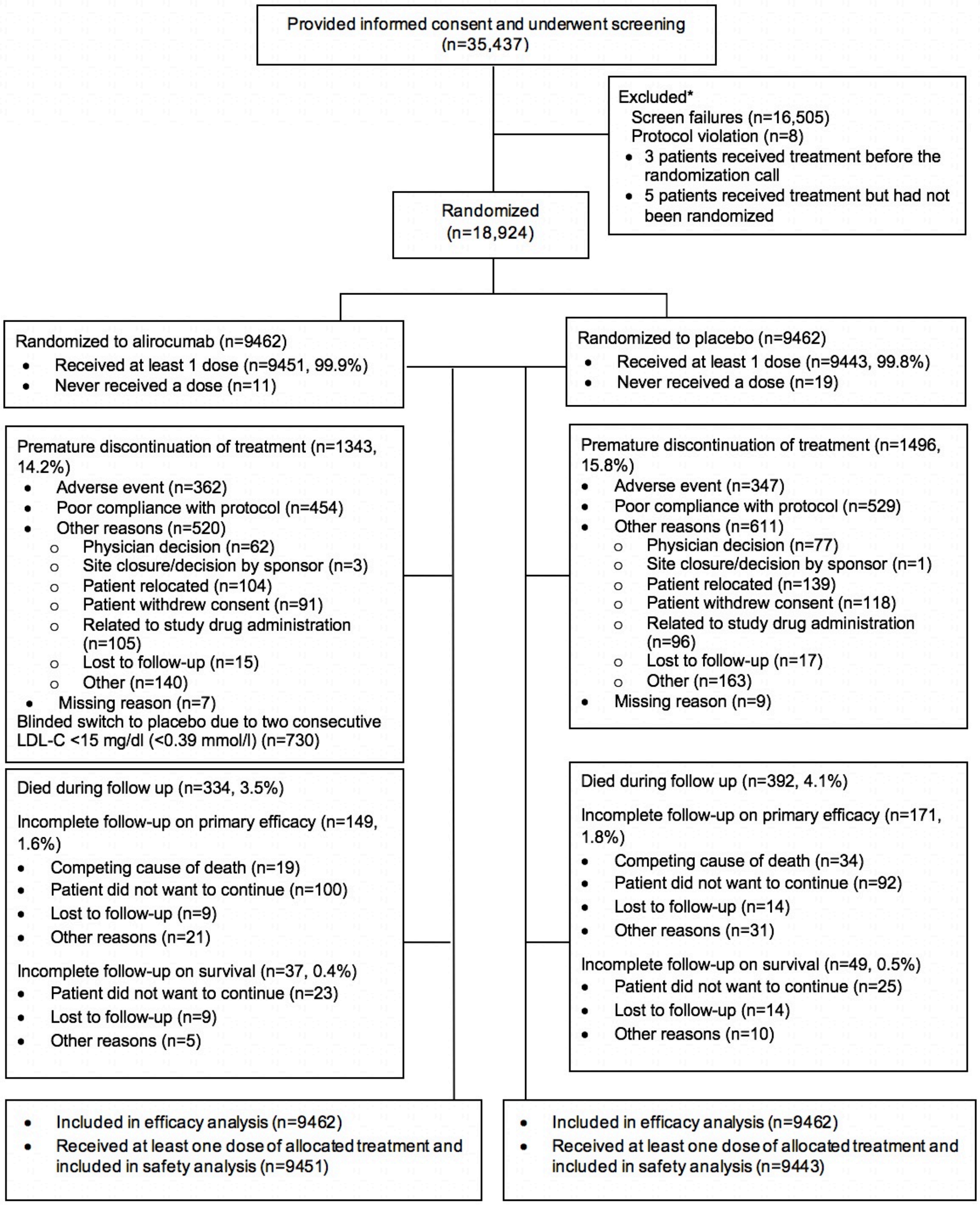

*The most common reasons for screen failures during the run-in period were related to lipid criteria (34.1% of patients) or withdrawal of patient consent (6.1% of patients).

Figure II Kaplan-Meier plots of cumulative MACE in strata of achieved LDL-C in the alirocumab group and in propensity score-matched patients from the placebo group.

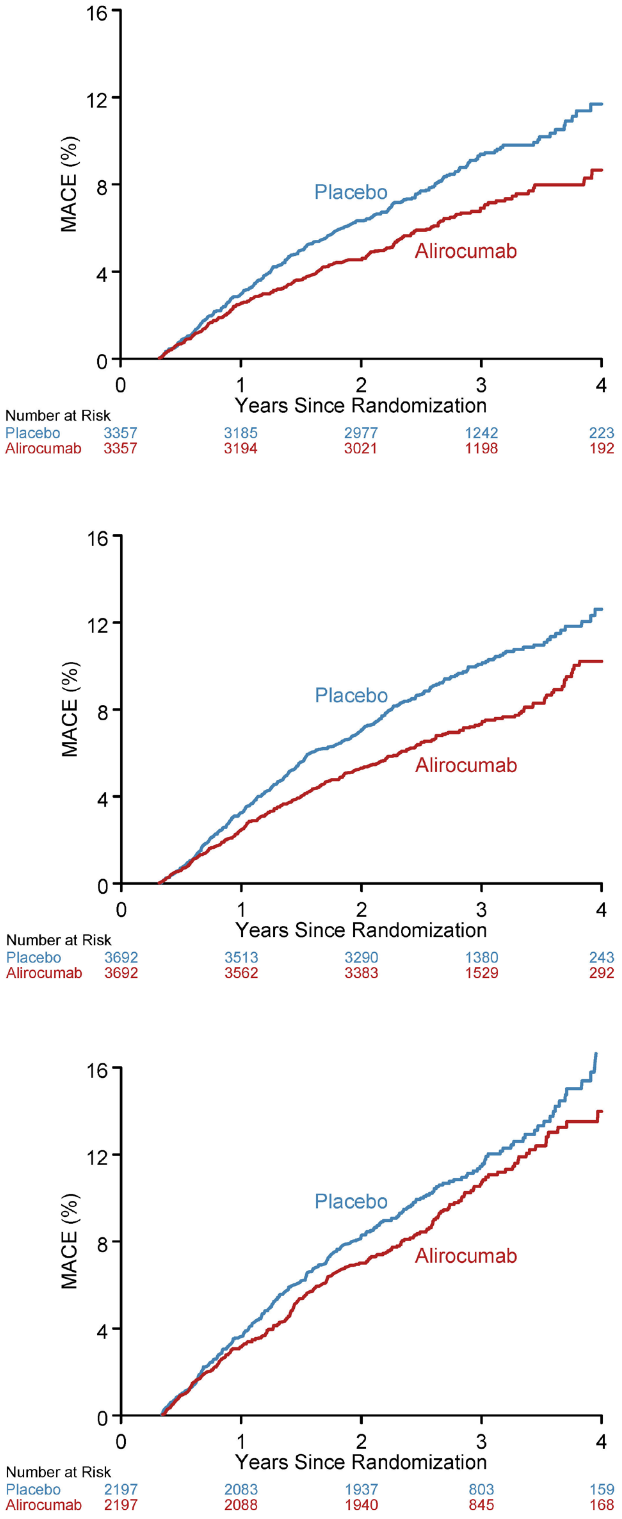

Achieved LDL-C at month 4 in the alirocumab group	Treatment HR (95% CI), alirocumab/propensity score-matched placebo
<25 mg/dL	0.74 (0.62–0.89)
25-50 mg/dL	0.74 (0.64–0.87)
>50 mg/dL	0.87 (0.73–1.04)
